# Use of Biological Feedback as a Health Behavior Change Technique in Adults: Scoping Review

**DOI:** 10.2196/44359

**Published:** 2023-09-25

**Authors:** Kelli M Richardson, Michelle R Jospe, Ahlam A Saleh, Thanatcha Nadia Clarke, Arianna R Bedoya, Nick Behrens, Kari Marano, Lacey Cigan, Yue Liao, Eric R Scott, Jessica S Guo, April Aguinaga, Susan M Schembre

**Affiliations:** 1 School of Nutritional Sciences and Wellness College of Agriculture, Life and Environmental Sciences University of Arizona Tucson, AZ United States; 2 Department of Oncology Lombardi Comprehensive Cancer Center Georgetown University Washington, DC United States; 3 Arizona Health Sciences Library University of Arizona Tucson, AZ United States; 4 Department of Physiology College of Medicine University of Arizona Tucson, AZ United States; 5 Department of Internal Medicine College of Medicine – Phoenix University of Arizona Tucson, AZ United States; 6 Department of Ecology and Evolutionary Biology College of Science University of Arizona Tucson, AZ United States; 7 College of Nursing University of Arizona Tucson, AZ United States; 8 Department of Kinesiology College of Nursing and Health Innovation University of Texas at Arlington Arlington, TX United States; 9 Communications & Cyber Technologies Arizona Experiment Station University of Arizona Tucson, AZ United States

**Keywords:** monitoring, physiologic, biomarkers, feedback, psychological, health behavior, health promotion, biological, adults, biosensing, technology, support, intervention, electronic database, cardiovascular disease, obesity, device

## Abstract

**Background:**

Recent advancements in personal biosensing technology support the shift from standardized to personalized health interventions, whereby biological data are used to motivate health behavior change. However, the implementation of interventions using biological feedback as a behavior change technique has not been comprehensively explored.

**Objective:**

The purpose of this review was to (1) map the domains of research where biological feedback has been used as a behavior change technique and (2) describe how it is implemented in behavior change interventions for adults.

**Methods:**

A comprehensive systematic search strategy was used to query 5 electronic databases (Ovid MEDLINE, Elsevier Embase, Cochrane Central Register of Controlled Trials, EBSCOhost PsycINFO, and ProQuest Dissertations & Theses Global) in June 2021. Eligible studies were primary analyses of randomized controlled trials (RCTs) in adults that incorporated biological feedback as a behavior change technique. DistillerSR was used to manage the literature search and review.

**Results:**

After removing 49,500 duplicates, 50,287 articles were screened and 767 articles were included. The earliest RCT was published in 1972 with a notable increase in publications after 2000. Biological feedback was most used in RCTs aimed at preventing or managing diabetes (n=233, 30.4%), cardiovascular disease (n=175, 22.8%), and obesity (n=115, 15%). Feedback was often given on multiple biomarkers and targeted multiple health behaviors. The most common biomarkers used were anthropometric measures (n=297, 38.7%), blood pressure (n=238, 31%), and glucose (n=227, 29.6%). The most targeted behaviors were diet (n=472, 61.5%), physical activity (n=417, 54.4%), and smoking reduction (n=154, 20.1%). The frequency and type of communication by which biological feedback was provided varied by the method of biomarker measurement. Of the 493 (64.3%) studies where participants self-measured their biomarker, 476 (96.6%) received feedback multiple times over the intervention and 468 (94.9%) received feedback through a biosensing device.

**Conclusions:**

Biological feedback is increasingly being used to motivate behavior change, particularly where relevant biomarkers can be readily assessed. Yet, the methods by which biological feedback is operationalized in intervention research varied, and its effectiveness remains unclear. This scoping review serves as the foundation for developing a guiding framework for effectively implementing biological feedback as a behavior change technique.

**Trial Registration:**

Open Science Framework Registries; https://doi.org/10.17605/OSF.IO/YP5WAd

**International Registered Report Identifier (IRRID):**

RR2-10.2196/32579

## Introduction

Many of the chronic diseases that make up the leading causes of death in the United States today, such as cardiovascular disease (CVD), type 2 diabetes, and many types of cancer, can be prevented or managed through personal behavior changes [1,2]. Adherence to recommended lifestyle behaviors, including a healthy diet, adequate physical activity, moderate alcohol consumption, and smoking abstinence, is known to reduce the risk of mortality and prolong life expectancy [3,4]. However, adopting and maintaining health behavior changes remain a significant challenge [5]. To improve behavior change and health-related outcomes, recent clinical research has shifted its focus from standardized one-size-fits-all health interventions to personalized health interventions [6,7]. Personalized (or precision) health interventions are those that are tailored to an individual’s characteristics such as their genotype, or behavioral or physiological phenotypes [6].

Recent advancements in personal sensing technologies and mobile health now offer new ways to approach personalized health [8,9]. Examples of technology that can facilitate the collection of biological data include connected body weight scales, connected glucose monitors (glucometers and continuous glucose monitors), and ambulatory blood pressure cuffs. Many of these tools have complementary software or share collected data with external software companies that aim to provide meaningful insights meant to motivate relevant health behavior changes for the users. However, the effectiveness of these tools (with and without additional feedback) has not been well characterized. It remains generally unclear how to effectively implement biological feedback within health behavior change interventions. A comprehensive review of intervention research that has provided feedback about one’s biological data to motivate health behavior change (ie, biological feedback) is essential to developing a guiding framework for implementing personalized health behavior change interventions that effectively motivate people to adopt and maintain health behavior changes.

Operationally, biological feedback is defined as providing an individual with their biological data to motivate a health behavior change [10]. One form of biological feedback is the behavior change technique, biofeedback. As defined by Michie et al [11], biofeedback is to “provide feedback about the body (eg, physiological or biochemical state) using an external monitoring device as part of a behavior change strategy.” Examples of external monitoring devices that can provide biofeedback include continuous glucose monitors and heart rate monitors. Notably, both biofeedback (as defined by Michie) and biological feedback differ from the traditional mind-body technique of “biofeedback,” which provides feedback on one’s autonomic nervous system to treat related conditions (eg, urinary incontinence) [12,13]. The purpose of both Michie’s biofeedback and the biological feedback herein is to motivate behavior change, which does not align with the purpose of the mind-body technique of biofeedback. Additionally, while Michie’s biofeedback focuses on biological feedback communicated through an external monitoring device, biological feedback herein encompasses all methods of sharing biological data, which may or may not have been collected using an external monitoring device. By including any method of communication, the evolution of this intervention approach prior to the existence of external monitoring devices can be examined. Given the broad scope of biological feedback and its potential within the uprising field of precision health, a comprehensive examination of its implementation within the literature is needed.

To our knowledge, the last review on biological feedback as a behavioral intervention was conducted in 2002 [14]. This review consisted of 8 randomized trials testing the effect of biological feedback on behavior change. The 3 behaviors targeted among the 8 interventions were diet, physical activity, and smoking reduction; and the biological data (herein also described as biomarkers) provided as feedback included carbon monoxide levels, genetics, pulmonary function, cholesterol levels, and an index of physical fitness. The effects of biological feedback on behavior change were mixed; however, a relationship between the intensity of biological feedback and the study outcome was observed. Studies resulting in significant behavior change (or intent to change behavior) were those that provided feedback on multiple biomarkers or on a single biomarker on more than one occasion [14]. While this review provided preliminary evidence of the efficacy of biological feedback as a behavior change technique, more work in this area needs to be done to help researchers better implement biological feedback within health behavior change interventions. As a first step to harnessing the potential of biological feedback, an updated, comprehensive review of biological feedback was needed.

The goal of this scoping review was to comprehensively examine the current and historical use of biological feedback as a behavior change strategy used in health behavior change interventions conducted in adults. The objectives were to (1) map the literature to examine which domains of research incorporated biological feedback as a health behavior change strategy and (2) describe the implementation characteristics of interventions that used biological feedback. Specifically, the questions answered by this review include the following: (1) In which domains or fields of research has biological feedback been implemented as a health behavior change technique in adults? (2) What health behaviors and health-related outcomes were targeted by interventions that incorporated biological feedback? (3) Which biological data were participants provided feedback about? (4) How are the biological data being collected, and in what frequency and by what method of communication are biological feedback being provided?

## Methods

### Overview

This scoping review was guided by the Joanna Briggs Institute Reviewer Manual [15] and follows the PRISMA-ScR (Preferred Reporting Items for Systematic Reviews and Meta-Analyses Extension for Scoping Reviews) checklist (Multimedia Appendix 1) [16]. It was registered in the Open Science Framework database [10]. A scoping review protocol with a full description of the methods used in this review has been published elsewhere [10].

### Search Strategy

A search strategy including more than 200 terms was initially developed in Ovid MEDLINE in June 2021 to encompass both broad terms to describe biological feedback (eg, personalized feedback and personal health monitoring) and terms specific to fields of research in which biological feedback could be used to promote health behavior change (eg, genetic counseling and blood glucose self-monitoring; Multimedia Appendix 2). Because there is yet to be consistent terminology for defining biological feedback, we worked closely with a research librarian to develop a search strategy that would capture intervention studies that integrated three key components of using biological feedback as a health behavior change technique: (1) biological data (including body weight, risk assessment, and genetic markers), (2) feedback (including health communication, self-testing, and wearable electronic devices), and (3) behavior change (including health behavior, healthy lifestyle, and risk reduction behavior). The search strategy was then designed to identify studies that included all 3 of these components. This search strategy was adapted to conform to each of the following electronic databases: Elsevier Embase, Cochrane Central Register of Controlled Trials, EBSCOhost PsycINFO, and ProQuest Dissertations & Theses Global. We used DistillerSR (Evidence Partners) systematic review and literature review software to manage the review. Bibliographies of relevant review articles that were returned by this search were reviewed and manually added to DistillerSR. Duplicate studies were identified and discarded first using EndNote (Clarivate Analytics) and then using the deduplicating function in DistillerSR. No restrictions were set on the year of publication or publication language.

### Study Screening and Selection

Given the size of this scoping review, 2 levels of title and abstract screening were performed in lieu of full-text screening, both of which were performed using DistillerSR. In phase I, 7 trained reviewers conducted independent screening of titles and abstracts of studies for initial eligibility. Inclusion criteria at this phase were based on study design (published primary analysis of a randomized controlled trial [RCT]) and study population (humans who were 18 years or older). An artificial intelligence function in DistillerSR was used for quality assurance to identify potentially erroneous exclusions made during this phase. An additional reviewer then re-examined the abstracts identified by artificial intelligence to determine if they met eligibility criteria. Phase II, which was also performed using the title and abstract in lieu of full-text screening, included a confirmation of the studies passing phase I, as well as the inclusion criterion of an intervention involving biological feedback being used to promote health behavior change. Additionally in phase II, if an abstract noted the use of self-monitoring, self-management, or a risk assessment (terms commonly associated with biological feedback) but did not note a biological data component, the full text for that study was retrieved and reviewed to confirm eligibility. Justification for using title and abstract screening in phase II has been provided in the scoping review protocol [10]. Briefly, we piloted this approach, whereby the title and abstract and the full text of 34 studies were independently reviewed for inclusion [10]. Agreement between studies excluded based on title and abstract review and full-text review was 96%, confirming the accuracy of this modified approach. For additional quality assurance, we used double data entry for all screening performed in phase II. Data entry conflicts were reviewed between the 2 reviewers who disagreed and were resolved by consensus. If the 2 reviewers could not come to an agreement, the conflict was resolved by a third trained reviewer.

### Data Extraction

A data extraction form was designed in DistillerSR and iteratively piloted by 3 trained reviewers. Two reviewers then performed double data extraction of the full text for the included studies. In addition to bibliographic information, the following data related to the characteristics of RCTs that implement biological feedback and the operational characteristics of implementing biological feedback were extracted: (1) bibliographic information (title, authors, and year of publication), (2) domain and area of research (eg, substance use and diabetes), (3) targeted health behavior, (4) targeted health-related outcome, (5) the biological data on which feedback was provided, (6) whether the biological data were used to calculate a composite score or value (eg, Framingham Risk Score and lung age), (7) how the biological data were assessed (eg, self-measured or measured in a clinical setting) (8) frequency of biological feedback (once or more than once), and (9) type of communication method by which biological feedback was provided (measurement device only; 1-way communication such as feedback via an app, mail, email, or 1-way text message; 2-way discussion such as an in-person discussion, video chat, phone call, or 2-way messaging).

Additionally, we extracted data about the theories, models, or frameworks cited by the included studies and whether it was used to guide intervention development. This information is included in Multimedia Appendix 3.

For purposes of feasibility, data specific to the way the biological data were collected and communicated were extracted at the study level (as opposed to the biomarker level). For example, if a study provided feedback on multiple biomarkers that were communicated through different methods, both methods were extracted.

Studies that passed the title and abstract screening phases but were found ineligible at the full-text data extraction phase were subsequently excluded from the review. Data entry conflicts were reviewed between the 2 reviewers and resolved. Extracted data were downloaded from DistillerSR for data cleaning using OpenRefine (formerly Google Refine), a free, open-source program used for cleaning large data sets [17].

## Results

### Search Results

**Figure 1:**
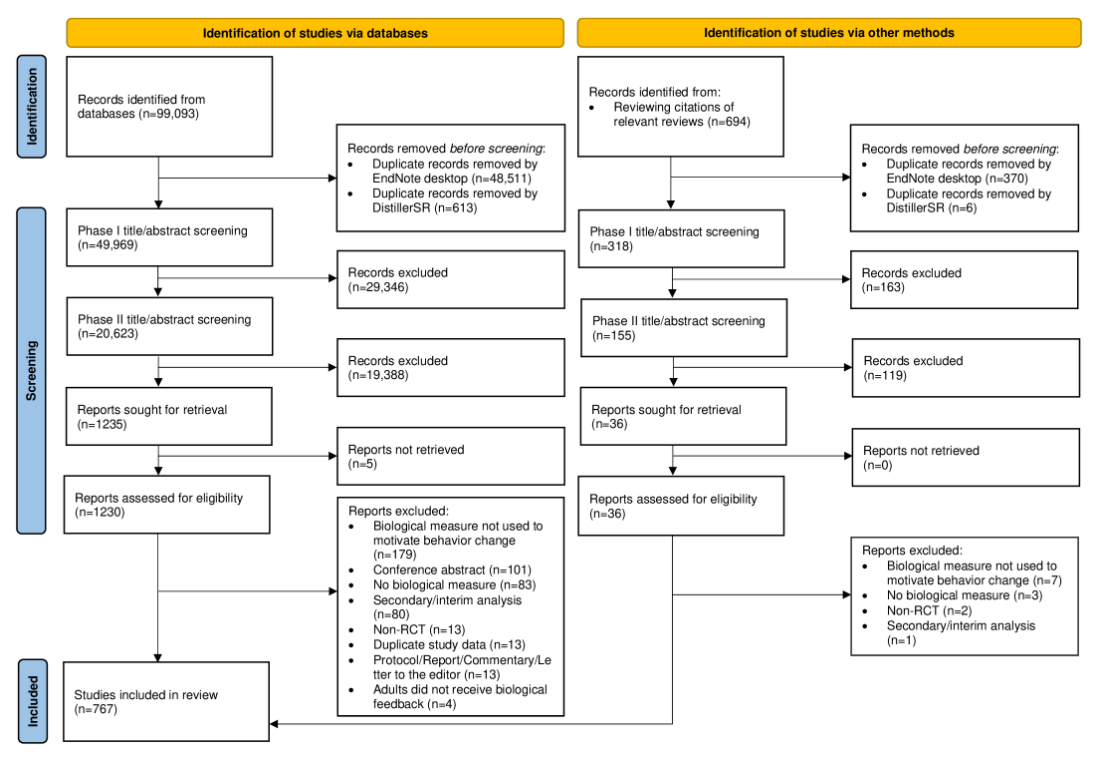
PRISMA (Preferred Reporting Items for Systematic Reviews and Meta-Analyses) flow diagram of included studies. RCT: randomized controlled trial.

A total of 99,093 studies were returned by our search strategy, and an additional 694 studies were identified through the review of bibliographies of relevant reviews; 49,500 duplicate studies were identified and removed. In total, 50,287 studies were screened in phase I and 20,778 passed onto phase II screening; of which, 1271 studies passed phase II screening, and 1266 full-text studies were successfully located for data extraction. During data extraction, an additional 499 studies were excluded for reasons outlined in the PRISMA diagram depicted in Figure 1, resulting in 767 studies being included in the scoping review. The reference list of the included studies can be located in our Zenodo repository [18] and in Multimedia Appendix 4.

### Characteristics of RCTs That Implemented Biological Feedback

**Figure 2:**
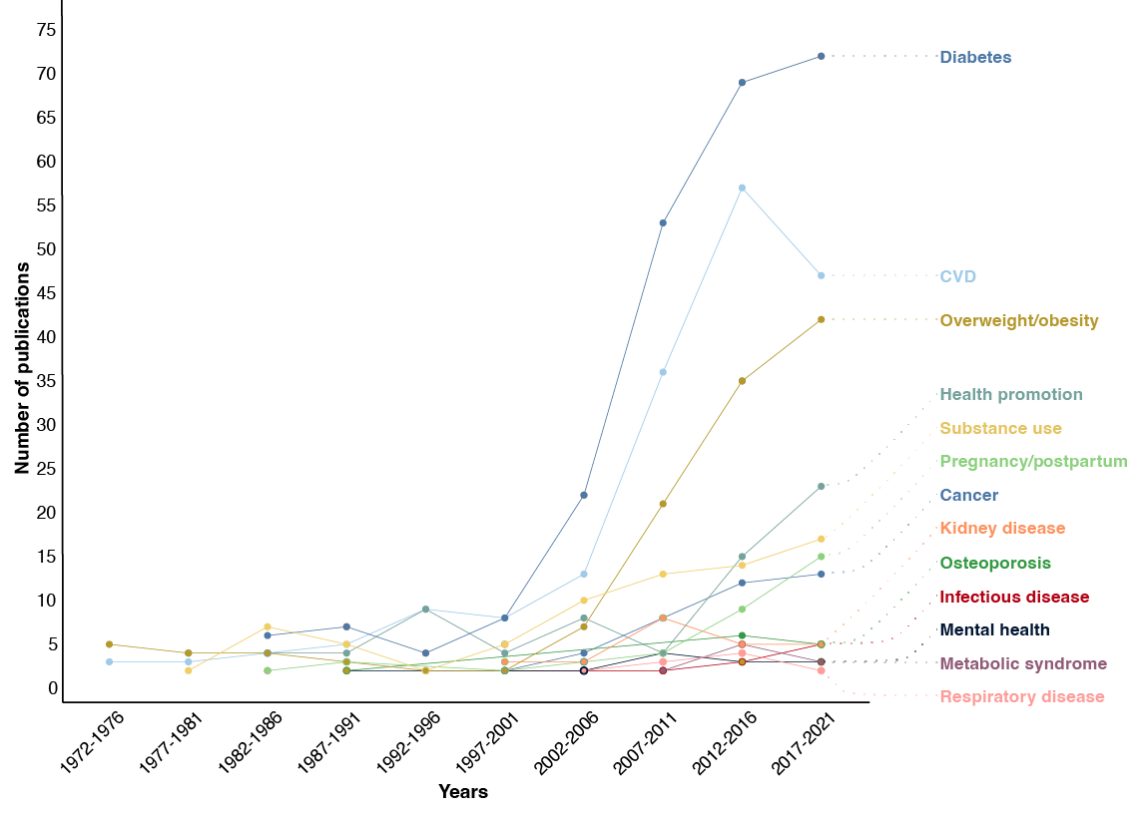
The use of biological feedback in randomized controlled trials from 1972 to 2021 (N=767). CVD: cardiovascular disease.

Five factors that were extracted to describe the characteristics of RCTs that implemented biological feedback as a health behavior change strategy were domain, targeted health-related outcome, biological data, whether the biological data were a component of a composite score, and targeted behavior. As depicted in Figure 2, the first RCTs to implement biological feedback as a behavior change technique were observed in 1972 in the domain of obesity and in 1975 in the domain of CVD. While the use of biological feedback in health behavior change interventions expanded to other domains of research (including substance use, diabetes, and pregnancy or postpartum) prior to 2000, the publication rate only notably increased after the year 2000. As of June 2021, the domains of research that most frequently used biological feedback were diabetes (n=233, 30.4%), CVD (n=175, 22.8%), and obesity (n=115, 15%). A complete table of domain frequency and first year of publication is presented in Multimedia Appendix 5.

The health-related outcomes most frequently targeted by RCTs that incorporated biological feedback were related to the most cited domains of research, including glycemic control (n=194, 25.3%), CVD management (n=162, 21.1%), and weight management (n=161, 21%) (Multimedia Appendix 6). The biomarkers in which feedback was most often provided were anthropometric measures such as weight, BMI, and body composition (n=297, 38.7%), blood pressure (n=238, 31%), and glucose (n=227, 29.6%) (Multimedia Appendix 7).

**Figure 3:**
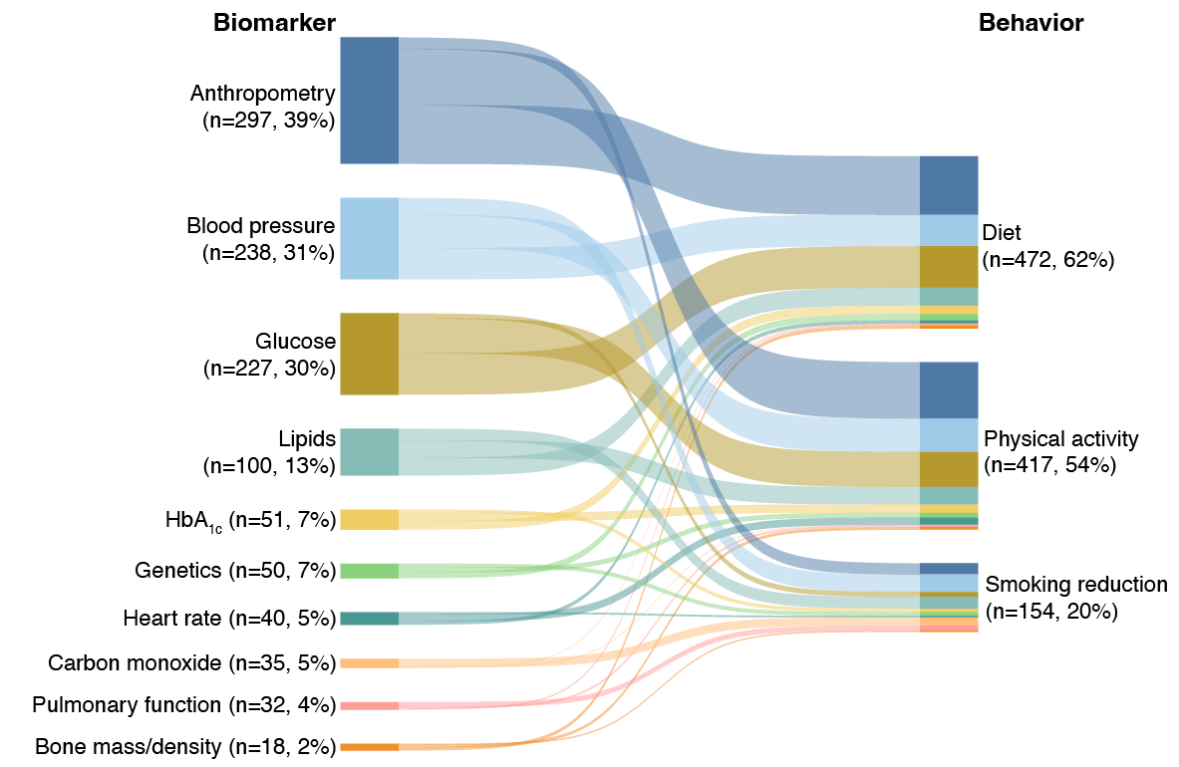
Most frequently assessed biomarkers and targeted behaviors (N=767). The thickness of each node (eg, “Anthropometry”) represents the number of studies that incorporated the given biomarker or behavior. The thickness of the links between the biomarkers and behaviors (eg, “Anthropometry” to “Diet”) represents the number of studies for which the given biomarker was used to promote the linked behavior. The total number of studies does not add up to 767 (100%) because only the top 10 biomarkers and top 3 behaviors are displayed. Additionally, some studies provided feedback on multiple biomarkers and targeted multiple behaviors. HbA1c: glycated hemoglobin.

In 223 articles (29.1%), feedback on more than 1 biomarker was provided. One or more biomarkers were used to compute a risk score in 97 (12.6%) of the included studies. As shown in Figure 3, feedback on the collected biomarkers was typically used to motivate changes in diet (n=472, 61.5%) and physical activity (n=417, 54.4%), and to motivate smoking reduction (n=154, 20.1%). However, in 90 studies (12%), the behavior that the biological feedback was targeting was unclear (eg, “lifestyle changes” were targeted, but the specific behaviors were not stated explicitly). A complete list of targeted health behaviors can be found in Multimedia Appendix 8.

### Operational Characteristics of Implementing Biological Feedback

**Figure 4:**
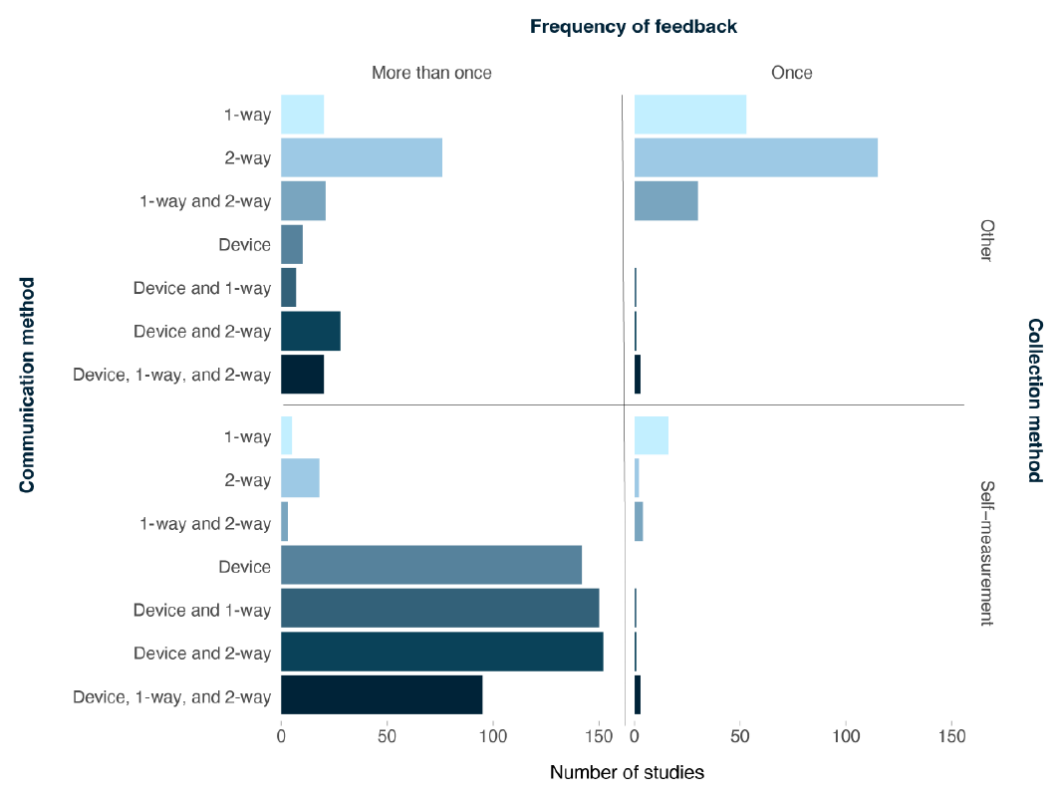
Type of communication by collection method and frequency of delivery (N=767). Biological feedback was communicated to participants through (1) the device itself, such as through a continuous glucose monitor or heart rate monitor, (2) 1-way communication, such as through an app, email, mail, or 1-way text message, (3) 2-way communication, such as an in-person discussion or 2-way messaging platform, or through a combination of these feedback modalities. Feedback on the biological data was provided either (1) once, such as a singular genetic test, or (2) more than once, such as multiple glucose tests. The biological data provided as feedback was either collected via (1) self-measurement, such as through a body weight scale or (2) other, such as a health care provider collecting a laboratory sample.

Three factors that were extracted to describe the operational characteristics of implementing biological feedback as a behavior change strategy were the biomarker collection method, the frequency, and the type of biological feedback communication. Most studies had participants self-measure their biological data (n=493, 64.3%) and biological feedback was often provided more than once (n=567, 73.9%). Notably, the frequency and type of communication were determined by how the biological data were assessed. As depicted in Figure 4, of the 493 (64.3%) studies that implemented biological feedback using self-measured biological data, they were more likely to provide feedback more than once (n=476, 96.6%) and through the measurement device either alone or in combination with 1-way or 2-way discussion (n=468, 94.9%). When the biomarker was not self-measured (n=332, 43.3%), the most common way it was communicated to participants was through a 2-way discussion (n=157, 47.3%).

Given the quantity of data retrieved from 767 studies, a comprehensive digital interactive visualization tool was developed to share our findings [19]. The data presented in the digital interactive visualization represent the characteristics of each of the studies included in this review (with searchable Digital Object Identifier, PubMed Identifier, or URLs), which can be filtered and exported as a downloadable CSV file.

## Discussion

### Principal Results

To our knowledge, this is the first scoping review to comprehensively map the domains of research using biological feedback as a health behavior change strategy and to characterize the procedures by which biological feedback has been implemented within behavior change interventions conducted in adults. The findings of this scoping review of more than 750 RCTs highlight an increasing use of biological feedback as a health behavior change strategy that rose sharply after the turn of the century, particularly in the domains of diabetes, CVD, and obesity research. A majority of the included studies provided feedback about biological data that can be easily measured, including anthropometric measures (weight, BMI, and body composition), blood pressure, and glucose that targeted changes in diet, physical activity, and smoking reduction. Method of measurement also determined the frequency and type of communication by which biological feedback was provided. While additional research is needed to determine the best approaches for incorporating biological feedback into health behavior interventions, this scoping review serves as a foundation for optimizing its effectiveness as a health behavior change technique.

### Comparison With Prior Work

Although RCTs incorporating biological feedback dated back to 1972, it was minimally used as a health behavior change technique until the year 2000 when an increase in its use was observed. This may be related to the beginning of the “personalized medicine” era, which was first introduced in 1999 to describe the individualization of pharmacotherapy for patients with cancer and has since influenced most other areas of our health care system [20], including research on diabetes, CVD, and obesity as noted in this review. While the concept of “personalized medicine” has broadened to include terms such as “precision medicine” and “precision/personalized health interventions,” the collective number of publications in this area has consistently risen over the past 2 decades, aligning with a similar observation in the publication rate of RCTs that incorporate biological feedback [20]. Consistent with this and previous reports [14], we found that interventions that incorporate biological feedback mainly target dietary, physical activity, and smoking behaviors. Likewise, in a 2022 review of precision health interventions, Mauch et al [7] noted that physical activity, dietary intake, and smoking are the most heavily targeted behaviors. This burgeoning area of research and clinical practice has received great recognition in recent years, particularly by the US government, which launched a Precision Medicine Initiative in 2015 aimed to enable a new era of individualized care in medicine through research, technology, and policy. As such, great advancements in the precision or personalized approach to health are expected in the coming decades.

Supporting this increase in the implementation of precision medicine interventions, there has also been a consistent increase in the use of biosensors and wearable sensor technology in research [21,22]. Unlike biomarkers such as lipids and HbA_1c_, which are most often measured in a clinical setting, the most frequently used biomarkers in biological feedback RCTs (ie, weight, blood pressure, and glucose) are more commonly self-measured with commercially available devices. Body weight scales, blood pressure monitors, and glucose monitors have been accessible for personal use since the 1920s, 1930s, and 1970s, respectively [23-25] and have since enabled passive and continuous data collection (eg, continuous glucose monitors) and function as smart devices (eg, connected body weight scales and smart blood pressure trackers) [26,27]. The increasing accessibility of personal biosensors is a great advantage afforded to personalized health interventions as well as a notable convenience to users as compared to clinic visits [28]. Furthermore, during the COVID-19 pandemic, there was an urgent demand for remote data collection due to quarantine restrictions that heightened the need for self-measured biological data [29,30]. Continued advancements in technology are likely to expand the types of biological data that can be self-measured, thereby creating new opportunities for integrating biological feedback into personalized health behavior interventions.

We also concluded that the biological data collection method (ie, self vs other) determined the frequency and type of communication by which biological feedback was provided, such that biological data that were self-measured were more likely to be offered multiple times over the intervention period and through a combination of communication methods that included the measurement device (eg, glucometers). While we were unable to draw conclusions about the effects of frequency and type of communication on biological feedback as a behavior change strategy from this scoping review, a previous review concluded that feedback that was continuously (or repeatedly) available, personalized, and actionable was prominent in intervention studies with significant behavior change [14]. A new review of recent precision health interventions also showed that a majority of studies provided feedback on multiple occasions and used self-reported or self-measured data, and feedback was delivered through the measurement device alone or in combination with 1- or 2-way communication [7]. Nonetheless, while the method of biological data collection, frequency, and type of communication of feedback may be related, there are instances where providing feedback on multiple occasions or through a device is not expected (eg, genotyping). Future research will be needed to determine how frequency and type of feedback over the duration of the intervention impact the effectiveness of biological feedback on health behavior change.

### Strengths and Limitations

Despite being the first scoping review to comprehensively summarize the use of biological feedback in behavioral interventions, there are several limitations. When developing our search strategy, we found there was inconsistent terminology used to describe biological feedback (eg, glucose monitoring is a form of biological feedback, but the study may not mention the term “biological feedback”). Therefore, not all biological feedback studies may have been captured by our search strategy. Nevertheless, this lack of consistency in terminology is not uncommon [31]. In light of this, a strength of this review is that a comprehensive list of over 200 search terms was developed to capture many possible forms of biological feedback in addition to the general terms relating to biological feedback. Another limitation is that the only type of study design that was included was primary RCTs. The primary reason for this decision was the feasibility of completing the review in a timely manner. While this approach is a strength in that it improves the quality of the 767 included studies, it did limit the breadth of the research being published in this area. However, due to the number of studies returned and the breadth of our search strategy, these limitations should not have restricted our ability to answer our research questions. Similarly, we limited our inclusion criteria to focus primarily on adults, which prevents the results from being generalized to younger populations. Another potential limitation is that we were unable to conduct full-text reviews of the large number of studies included after a single round of screening abstracts and titles. While this may have led to some erroneous exclusion of articles, it is unlikely given the success of our pilot for conducting a second round of screening abstracts [10] and our use of DistillerSR’s artificial intelligence feature for identifying accidentally excluded studies. Finally, when extracting the data, we extracted it on a study-level basis rather than on a biomarker-level basis. Therefore, when summarizing the data, we could not make conclusions about how specific biological feedback was delivered. Nevertheless, a strength of the review is that the comprehensive digital interactive visualization allows researchers interested in a specific biomarker to filter the data and view relevant studies to guide their decisions in delivering biological data as feedback.

### Conclusions

Biological feedback is increasingly being used as a health behavior change technique across multiple domains of research and health behaviors. What is additionally evident from this review is that, with increasing and broadening use, the methods by which biological feedback is implemented into health behavior interventions (ie, biological data collection and type and frequency of communication) are varied. Notably, however, developing a guiding framework to implement effective health behavior interventions that incorporate biological feedback will be challenging given the diversity of biological data being used, the multitude of behaviors those data are linked to, and the fact that most often, health behavior interventions use multiple behavior change techniques. Despite this challenge, this scoping review (and the accompanying digital visualization and data sharing tool) represents significant progress toward optimizing personalized (or precision) health interventions that incorporate biological feedback.

## Appendix

Multimedia Appendix 1 PRISMA-ScR checklist.

Multimedia Appendix 2 The search strategy.

Multimedia Appendix 3 Behavior change theories, models, and frameworks cited in biological feedback literature and the domains of research in which they were cited.

Multimedia Appendix 4 References of articles included in the review.

Multimedia Appendix 5 Domains of research that have used biological feedback to promote health behavior change in adults (N=767).

Multimedia Appendix 6 Outcomes targeted by biological feedback interventions in adults (N=767).

Multimedia Appendix 7 Biomarkers on which biological feedback has been provided (N=767).

Multimedia Appendix 8 Behaviors targeted by biological feedback interventions (N=767).

## Abbreviations

CVD: cardiovascular disease
HbA_1c_: glycated hemoglobin
PRISMA-ScR: Preferred Reporting Items for Systematic Reviews and Meta-Analyses Extension for Scoping Reviews
RCT: randomized controlled trial

## Data Availability

The data set supporting the conclusions of this review are available in the Biological Feedback Scoping Review Zenodo Repository [18]. The data can also be downloaded through our digital interactive visualization [19].
