# Supplementary material for: Use of Biological Feedback as a Health Behavior Change Technique in Adults: Scoping Review
Source: J Med Internet Res. 2023 Sep 25;25:e44359. doi: 10.2196/44359 (PMC10562972; doi:10.2196/44359)
Supplement: Multimedia Appendix 2 [file jmir_v25i1e44359_app2.docx]

Search Documentation:

Ovid MEDLINE(R) and Epub Ahead of Print, In-Process, In-Data-Review & Other Non-Indexed Citations, Daily and Versions(R) <1946 to June 01, 2021>

Date searched: 6/02/21

| # | Searches |
| --- | --- |
| 1 | biological phenomena/ |
| 2 | biomarkers/ |
| 3 | physiological phenomena/ |
| 4 | monitoring, physiologic/ |
| 5 | biological monitoring/ |
| 6 | body weight/ |
| 7 | risk assessment/ |
| 8 | basal metabolism/ |
| 9 | blood glucose/ |
| 10 | blood pressure/ |
| 11 | exp body composition/ |
| 12 | "body weights and measures"/ |
| 13 | exp "body fat distribution"/ |
| 14 | "body mass index"/ |
| 15 | "waist-hip ratio"/ |
| 16 | exp body size/ |
| 17 | exp blood cells/ |
| 18 | blood.fs. |
| 19 | urine.fs. |
| 20 | cholesterol/ |
| 21 | gastrointestinal microbiome/ |
| 22 | genetic predisposition to disease/ |
| 23 | genetic profile/ |
| 24 | genetic markers/ |
| 25 | genetic carrier screening/ |
| 26 | genetic testing/ |
| 27 | health status indicators/ |
| 28 | health status/ |
| 29 | heart rate/ |
| 30 | exp muscle strength/ |
| 31 | nutritional status/ |
| 32 | pulse/ |
| 33 | respiratory rate/ |
| 34 | vital signs/ |
| 35 | breath tests/ |
| 36 | glycated hemoglobin a/ |
| 37 | anthropometry/ |
| 38 | blood chemical analysis/ |
| 39 | health impact assessment/ |
| 40 | precision medicine/ |
| 41 | diagnostic tests, routine/ |
| 42 | exp respiratory function tests/ |
| 43 | exp spirometry/ |
| 44 | exp oximetry/ |
| 45 | blood pressure determination/ |
| 46 | "direct-to-consumer screening and testing"/ |
| 47 | exp disease susceptibility/ |
| 48 | exp exercise test/ |
| 49 | heart rate determination/ |
| 50 | hematologic tests/ |
| 51 | exp blood cell counts/ |
| 52 | physical examination/ |
| 53 | carbon monoxide/ |
| 54 | nutritional physiological phenomena/ |
| 55 | nutrition assessment/ |
| 56 | galvanic skin response/ |
| 57 | urinalysis/ |
| 58 | ethanol/ |
| 59 | "blood alcohol content"/ |
| 60 | exp "Diagnostic Imaging"/ |
| 61 | "diagnostic imaging".fs. |
| 62 | electrooculography/ |
| 63 | exp "Electrocardiography"/ |
| 64 | exp "Plethysmography"/ |
| 65 | cotinine/ |
| 66 | metabolic equivalent/ |
| 67 | ketones/ |
| 68 | ketone bodies/ |
| 69 | bone density/ |
| 70 | "absorptiometry, photon"/ |
| 71 | electric impedance/ |
| 72 | (biological adj3 (data or information or metric* or marker* or measure* or indicator* or risk*)).tw. |
| 73 | biomarker*.tw. |
| 74 | (blood adj3 analyte*).tw. |
| 75 | metabolite*.tw. |
| 76 | ("gut microbiome" or "gut microflora" or "gut bacteria" or "gut microbiota").tw. |
| 77 | nutrigenetic*.tw. |
| 78 | epigenetic*.tw. |
| 79 | "risk indicator*".tw. |
| 80 | "risk appraisal*".tw. |
| 81 | "health hazard appraisal*".tw. |
| 82 | (modifiable adj3 "risk factor*").tw. |
| 83 | ("health status" adj3 indicator*).tw. |
| 84 | glucose.tw. |
| 85 | (HgA1c or HbA1c or "h?emoglobin A1c").tw. |
| 86 | ("glycated h?emoglobin" or "glycosylated h?emoglobin").tw. |
| 87 | anthropom*.tw. |
| 88 | "blood pressure*".tw. |
| 89 | weight.tw. |
| 90 | BMI.tw. |
| 91 | "body mass index".tw. |
| 92 | "body measurement*".tw. |
| 93 | "body composition".tw. |
| 94 | "waist hip".tw. |
| 95 | "waist circumference".tw. |
| 96 | "carbon monoxide".tw. |
| 97 | ("genetic susceptibility" or "genetic predisposition").tw. |
| 98 | (genetic adj1 (risk* or test* or screen*)).tw. |
| 99 | (blood adj3 test*).tw. |
| 100 | ("lung function" adj3 test*).tw. |
| 101 | "exercise test".tw. |
| 102 | "muscle strength".tw. |
| 103 | "personali#ed risk".tw. |
| 104 | "heart rate".tw. |
| 105 | "lipid profile".tw. |
| 106 | cholesterol.tw. |
| 107 | "liver enzyme*".tw. |
| 108 | "galvanic skin response".tw. |
| 109 | "skin conductance response".tw. |
| 110 | (electrodermal adj1 (activity or response)).tw. |
| 111 | urinalysis.tw. |
| 112 | (ultrasound or ultrasonogr*).tw. |
| 113 | "computed tomogr*".tw. |
| 114 | electro#ardiogra*.tw. |
| 115 | (ekg or ecg).tw. |
| 116 | plethysmography.tw. |
| 117 | cotinine.tw. |
| 118 | spirometry.tw. |
| 119 | "breath test*".tw. |
| 120 | "risk assessment".tw. |
| 121 | ("precision nutrition" or "precision medicine").tw. |
| 122 | electrooculography.tw. |
| 123 | (ethanol or "blood alcohol").tw. |
| 124 | "pulmonary function test*".tw. |
| 125 | ketone*.tw. |
| 126 | ("beta-hydroxybutyrate" or "3-hydroxybutyric acid").tw. |
| 127 | ("bone density" or "dexa scan" or "dxa scan" or "dual energy x-ray absorptiometry").tw. |
| 128 | bod?pod.tw. |
| 129 | "underwater weigh*".tw. |
| 130 | "hydrostatic weigh*".tw. |
| 131 | hydrodensitometry.tw. |
| 132 | or/1-131 |
| 133 | feedback/ |
| 134 | feedback, psychological/ |
| 135 | communication/ |
| 136 | health communication/ |
| 137 | health education/ |
| 138 | patient education as topic/ |
| 139 | cues/ |
| 140 | genetic counseling/ |
| 141 | counseling/ |
| 142 | motivational interviewing/ |
| 143 | exp "correspondence as topic"/ |
| 144 | "behavior therapy"/ |
| 145 | therapy.fs. and lifestyle/ |
| 146 | wearable electronic devices/ |
| 147 | exp monitoring, ambulatory/ |
| 148 | exp self-testing/ |
| 149 | telephone/ |
| 150 | exp cell-phone/ |
| 151 | feedback*.tw. |
| 152 | ((communicat* or provide*) adj3 (results or information or risk*)).tw. |
| 153 | "cue* to action".tw. |
| 154 | ((tailored or personali#ed or individuali#ed) adj5 (communicat* or information)).tw. |
| 155 | (receiv* adj3 (results or information)).tw. |
| 156 | ((knowledge or knowing) adj3 result*).tw. |
| 157 | counsel*.tw. |
| 158 | coach*.tw. |
| 159 | advice.tw. |
| 160 | "motivational interviewing".tw. |
| 161 | (behavio?r* adj1 (therap* or intervention*)).tw. |
| 162 | ((tailored or personali#ed or individuali#ed) adj3 educat*).tw. |
| 163 | (education* adj3 (module or support or session)).tw. |
| 164 | (educate adj3 (individual* or patient*)).tw. |
| 165 | (received adj3 education*).tw. |
| 166 | ((patient or health) adj1 education).tw. |
| 167 | wearable*.tw. |
| 168 | biosensor*.tw. |
| 169 | "sensor technolog*".tw. |
| 170 | (device adj3 (monitor* or track*)).tw. |
| 171 | "smart device*".tw. |
| 172 | (self adj3 (monitor* or test*)).tw. |
| 173 | "ambulatory monitor*".tw. |
| 174 | ("personal* health" adj3 monitor*).tw. |
| 175 | messag*.tw. |
| 176 | (telephone* or cellphone* or cell-phone* or smartphone* or smart-phone* or "mobile phone*").tw. |
| 177 | ((month* or week*) adj3 support).tw. |
| 178 | ((interpreted or reported or informed) adj3 (measurement* or result* or data or level*)).tw. |
| 179 | or/133-178 |
| 180 | 132 and 179 |
| 181 | "blood glucose self-monitoring"/ |
| 182 | "blood pressure monitors"/ or "Blood Pressure Monitoring, Ambulatory"/ |
| 183 | ((home or self or continuous or ambulatory) adj3 ("blood pressure monitor*" or "glucose monitor*")).tw. |
| 184 | "flash glucose monitor*".tw. |
| 185 | ("hr monitor*" or "heart rate monitor*").tw. |
| 186 | "physiological feedback".tw. |
| 187 | "biological feedback".tw. |
| 188 | biofeedback.tw. |
| 189 | self-weigh*.tw. |
| 190 | "daily weigh*".tw. |
| 191 | (connected adj1 (glucometer* or scale*)).tw. |
| 192 | "smart scale*".tw. |
| 193 | (BIA adj3 scale*).tw. |
| 194 | (("bio?electric* impedance" adj3 (scale* or analysis)) or (bio?impedance adj3 (scale* or analysis))).tw. |
| 195 | or/181-194 |
| 196 | 180 or 195 |
| 197 | behavior/ |
| 198 | exp health behavior/ |
| 199 | behavior control/ |
| 200 | behavioral medicine/ |
| 201 | behavioral research/ |
| 202 | feeding behavior/ |
| 203 | health, knowledge, attitudes, practice/ |
| 204 | exp healthy lifestyle/ |
| 205 | exp health promotion/ |
| 206 | exp motivation/ |
| 207 | risk reduction behavior/ |
| 208 | self-efficacy/ |
| 209 | self-care/ |
| 210 | self-management/ |
| 211 | awareness/ |
| 212 | exp inhibition, psychological/ |
| 213 | "Treatment Adherence and Compliance"/ |
| 214 | Patient Compliance/ |
| 215 | patient participation/ |
| 216 | public health/ |
| 217 | public health practice/ |
| 218 | preventive medicine/ |
| 219 | prevention & control.fs. |
| 220 | preventive health services/ |
| 221 | exp primary prevention/ |
| 222 | secondary prevention/ |
| 223 | tertiary prevention/ |
| 224 | smoking prevention/ |
| 225 | harm reduction/ |
| 226 | treatment outcome/ and (lifestyle/ or psychology.fs.) |
| 227 | ((behavio?r* or lifestyle) adj3 (chang* or modif* or promot*)).tw. |
| 228 | "health behavio?r*".tw. |
| 229 | "healthy lifestyle".tw. |
| 230 | (self adj3 (care or management or efficacy)).tw. |
| 231 | awareness.tw. |
| 232 | ((risk or harm or "sedentary behavio?r") adj3 reduc*).tw. |
| 233 | "weight loss".tw. |
| 234 | "weight control".tw. |
| 235 | (smok* adj3 (behavio?r* or cessation or quit*)).tw. |
| 236 | "self regulat*".tw. |
| 237 | (motivated or motivation).tw. |
| 238 | (adherence or compliance).tw. |
| 239 | (prevention or preventive).tw. |
| 240 | "health promotion".tw. |
| 241 | (improv* adj3 (activit* or eating or diet* or health or fitness)).tw. |
| 242 | ((exercise or "physical activity" or diet* or eating or weight) adj3 (behavio?r* or chang* or maint* or motivat* or promot* or modif*)).tw. |
| 243 | "public health".tw. |
| 244 | or/197-243 |
| 245 | 196 and 244 |
| 246 | limit 245 to medline |
| 247 | 245 not 246 |
| 248 | randomized controlled trial.pt. |
| 249 | controlled clinical trial.pt. |
| 250 | randomi#ed.ab. |
| 251 | clinical trials as topic.sh. |
| 252 | randomly.ab. |
| 253 | trial.ti. |
| 254 | 248 or 249 or 250 or 251 or 252 or 253 |
| 255 | exp animals/ not humans.sh. |
| 256 | 254 not 255 |
| 257 | 246 and 256 |
| 258 | random*.tw. |
| 259 | trial.tw. |
| 260 | 258 or 259 |
| 261 | 247 and 260 |
| 262 | 257 or 261 |

Embase.com Embase
Date searched: 6/03/21

| No. | Query |
| --- | --- |
| #230 | #229 AND [embase]/lim |
| #229 | #227 AND #228 |
| #228 | 'crossover procedure':de OR 'double-blind procedure':de OR 'randomized controlled trial':de OR 'single-blind procedure':de OR random*:de,ab,ti OR factorial*:de,ab,ti OR crossover*:de,ab,ti OR ((cross NEXT/1 over*):de,ab,ti) OR placebo*:de,ab,ti OR ((doubl* NEAR/1 blind*):de,ab,ti) OR ((singl* NEAR/1 blind*):de,ab,ti) OR assign*:de,ab,ti OR allocat*:de,ab,ti OR volunteer*:de,ab,ti |
| #227 | #183 AND #226 |
| #226 | #184 OR #185 OR #186 OR #187 OR #188 OR #189 OR #190 OR #191 OR #192 OR #193 OR #194 OR #195 OR #196 OR #197 OR #198 OR #199 OR #200 OR #201 OR #202 OR #203 OR #204 OR #205 OR #206 OR #207 OR #208 OR #209 OR #210 OR #211 OR #212 OR #213 OR #214 OR #215 OR #216 OR #217 OR #218 OR #219 OR #220 OR #221 OR #222 OR #223 OR #224 OR #225 |
| #225 | 'public health':ti,ab |
| #224 | ((exercise OR 'physical activity' OR diet* OR eating OR weight) NEAR/3 (change OR behavior* OR behaviour* OR modif* OR maint* OR motivat* OR promot*)):ti,ab |
| #223 | (improv* NEAR/3 (activit* OR eating OR diet* OR health OR fitness)):ti,ab |
| #222 | 'health promotion':ti,ab |
| #221 | prevention:ti,ab OR preventive:ti,ab |
| #220 | adherence:ti,ab OR compliance:ti,ab |
| #219 | motivated:ti,ab OR motivation:ti,ab |
| #218 | 'self regulat*':ti,ab |
| #217 | (smok* NEAR/3 (behavior* OR behaviour* OR cessation OR quit*)):ti,ab |
| #216 | 'weight control':ti,ab |
| #215 | 'weight loss':ti,ab |
| #214 | ((risk OR harm OR 'sedentary behavior' OR 'sedentary behaviour') NEAR/3 reduc*):ti,ab |
| #213 | awareness:ti,ab |
| #212 | (self NEAR/3 (care OR management OR efficacy)):ti,ab |
| #211 | 'healthy lifestyle*':ti,ab |
| #210 | 'health behavior*':ti,ab OR 'health behaviour*':ti,ab |
| #209 | ((behavior* OR behaviour* OR lifestyle) NEAR/3 (change* OR modif* OR promot*)):ti,ab |
| #208 | 'treatment outcome'/de AND (psychology:de OR 'lifestyle'/de) |
| #207 | 'smoking prevention'/de |
| #206 | 'tertiary prevention'/de |
| #205 | 'secondary prevention'/de |
| #204 | 'primary prevention'/de |
| #203 | 'prevention'/de |
| #202 | 'preventive health service'/de |
| #201 | prevention:lnk OR 'prevention and control'/de |
| #200 | 'preventive medicine'/de |
| #199 | 'public health'/de |
| #198 | 'patient participation'/de |
| #197 | 'patient compliance'/exp |
| #196 | 'inhibition (psychology)'/exp |
| #195 | 'awareness'/de |
| #194 | 'self care'/de |
| #193 | 'risk reduction'/de |
| #192 | 'motivation'/exp |
| #191 | 'health promotion'/de |
| #190 | 'lifestyle modification'/de |
| #189 | 'healthy lifestyle'/de |
| #188 | 'feeding behavior'/de |
| #187 | 'behavior change'/de |
| #186 | 'behavior control'/de |
| #185 | 'health behavior'/exp |
| #184 | 'behavior'/de |
| #183 | #167 OR #182 |
| #182 | #168 OR #169 OR #170 OR #171 OR #172 OR #173 OR #174 OR #175 OR #176 OR #177 OR #178 OR #179 OR #180 OR #181 |
| #181 | ((('bioelectric* impedance' OR 'bio electric* impedance') NEAR/3 (scale* OR analysis)):ti,ab) OR (((bioimpedance OR 'bio impedance') NEAR/3 (scale* OR analysis)):ti,ab) |
| #180 | (bia NEAR/3 scale*):ti,ab |
| #179 | 'smart scale*':ti,ab |
| #178 | (connected NEAR/1 (glucometer* OR scale*)):ti,ab |
| #177 | 'daily weigh*':ti,ab |
| #176 | 'self weigh*':ti,ab |
| #175 | biofeedback:ti,ab |
| #174 | 'biological feedback':ti,ab |
| #173 | 'physiological feedback':ti,ab |
| #172 | 'hr monitor*':ti,ab OR 'heart rate monitor*':ti,ab |
| #171 | 'flash glucose monitor*':ti,ab |
| #170 | ((home OR self OR continuous OR ambulatory) NEAR/3 ('blood pressure monitor*' OR 'glucose monitor*')):ti,ab |
| #169 | 'blood pressure monitor'/de OR 'non invasive blood pressure monitor'/de |
| #168 | 'blood glucose monitoring'/de |
| #167 | #123 AND #166 |
| #166 | #124 OR #125 OR #126 OR #127 OR #128 OR #129 OR #130 OR #131 OR #132 OR #133 OR #134 OR #135 OR #136 OR #137 OR #138 OR #139 OR #140 OR #141 OR #142 OR #143 OR #144 OR #145 OR #146 OR #147 OR #148 OR #149 OR #150 OR #151 OR #152 OR #153 OR #154 OR #155 OR #156 OR #157 OR #158 OR #159 OR #160 OR #161 OR #162 OR #163 OR #164 OR #165 |
| #165 | ((interpreted OR reported OR informed) NEAR/3 (measurement* OR result* OR data OR level*)):ti,ab |
| #164 | ((month* OR week*) NEAR/3 support):ti,ab |
| #163 | telephone*:ti,ab OR cellphone*:ti,ab OR 'cell phone':ti,ab OR 'cell phones':ti,ab OR smartphone*:ti,ab OR 'smart phone':ti,ab OR 'smart phones':ti,ab OR 'mobile phone':ti,ab OR 'mobile phones':ti,ab |
| #162 | messag*:ti,ab |
| #161 | (('personal health' OR 'personalized health' OR 'personalised health') NEAR/3 monitor*):ti,ab |
| #160 | 'ambulatory monitor':ti,ab OR 'ambulatory monitors':ti,ab |
| #159 | (self NEAR/3 (monitor* OR test*)):ti,ab |
| #158 | 'smart device':ti,ab OR 'smart devices':ti,ab |
| #157 | (device NEAR/3 (monitor* OR track*)):ti,ab |
| #156 | 'sensor technology':ti,ab OR 'sensor technologies':ti,ab |
| #155 | biosensor*:ti,ab |
| #154 | wearable*:ti,ab |
| #153 | ((patient OR health) NEAR/1 education):ti,ab |
| #152 | (received NEAR/3 education*):ti,ab |
| #151 | (educate NEAR/3 (individual* OR patient*)):ti,ab |
| #150 | (education* NEAR/3 (module OR support OR session)):ti,ab |
| #149 | ((tailored OR personalized OR personalised OR individualized OR individualised) NEAR/3 educat*):ti,ab |
| #148 | ((behavior OR behaviour) NEAR/1 (therap* OR intervention*)):ti,ab |
| #147 | 'motivational interviewing':ti,ab |
| #146 | advice:ti,ab |
| #145 | coach*:ti,ab |
| #144 | counsel*:ti,ab |
| #143 | ((knowledge OR knowing) NEAR/3 result*):ti,ab |
| #142 | (receiv* NEAR/3 (results OR information)):ti,ab |
| #141 | ((tailored OR personalized OR personalised OR individualized OR individualised) NEAR/5 (communicat* OR information)):ti,ab |
| #140 | 'cue to action':ti,ab OR 'cues to action':ti,ab |
| #139 | ((communicat* OR provide*) NEAR/3 (results OR information OR risk*)):ti,ab |
| #138 | feedback*:ti,ab |
| #137 | 'mobile phone'/exp |
| #136 | 'telephone'/de |
| #135 | 'self-testing'/de |
| #134 | 'ambulatory monitoring'/de |
| #133 | 'wearable computer'/exp |
| #132 | 'therapy':lnk AND 'lifestyle'/de |
| #131 | 'behavior therapy'/de |
| #130 | 'motivational interviewing'/de |
| #129 | 'counseling'/de |
| #128 | 'genetic counseling'/de |
| #127 | 'patient education'/de OR 'diabetes education'/de OR 'nutrition education'/de |
| #126 | 'health education'/de |
| #125 | 'interpersonal communication'/de |
| #124 | 'feedback system'/exp |
| #123 | #1 OR #2 OR #3 OR #4 OR #5 OR #6 OR #7 OR #8 OR #9 OR #10 OR #11 OR #12 OR #13 OR #14 OR #15 OR #16 OR #17 OR #18 OR #19 OR #20 OR #21 OR #22 OR #23 OR #24 OR #25 OR #26 OR #27 OR #28 OR #29 OR #30 OR #31 OR #32 OR #33 OR #34 OR #35 OR #36 OR #37OR #38 OR #39 OR #40 OR #41 OR #42 OR #43 OR #44 OR #45 OR #46 OR #47 OR #48 OR #49 OR #50 OR #51 OR #52 OR #53 OR #54 OR #55 OR #56 OR #57 OR #58 OR #59 OR #60 OR #61 OR #62 OR #63 OR #64 OR #65 OR #66 OR #67 OR #68 OR #69 OR #70 OR #71 OR #72OR #73 OR #74 OR #75 OR #76 OR #77 OR #78 OR #79 OR #80 OR #81 OR #82 OR #83 OR #84 OR #85 OR #86 OR #87 OR #88 OR #89 OR #90 OR #91 OR #92 OR #93 OR #94 OR #95 OR #96 OR #97 OR #98 OR #99 OR #100 OR #101 OR #102 OR #103 OR #104 OR #105 OR #106OR #107 OR #108 OR #109 OR #110 OR #111 OR #112 OR #113 OR #114 OR #115 OR #116 OR #117 OR #118 OR #119 OR #120 OR #121 OR #122 |
| #122 | 'hydrostatic weigh*':ti,ab OR hydrodensitometry:ti,ab |
| #121 | 'underwater weigh*':ti,ab |
| #120 | bodpod:ti,ab OR 'bod pod':ti,ab |
| #119 | 'dual energy x-ray absorptiometry':ti,ab |
| #118 | 'bone density':ti,ab OR 'dexa scan':ti,ab OR 'dxa scan':ti,ab |
| #117 | 'beta-hydroxybutyrate':ti,ab OR '3-hydroxybutyric acid':ti,ab |
| #116 | ketone*:ti,ab |
| #115 | 'pulmonary function test*':ti,ab |
| #114 | ethanol:ti,ab OR 'blood alcohol':ti,ab |
| #113 | electrooculography:ti,ab |
| #112 | 'precision nutrition':ti,ab OR 'precision medicine':ti,ab |
| #111 | 'breath test*':ti,ab |
| #110 | spirometry:ti,ab |
| #109 | cotinine:ti,ab |
| #108 | plethysmography:ti,ab |
| #107 | ekg:ti,ab OR ecg:ti,ab |
| #106 | electrocardiogra*:ti,ab OR electrokardiogra*:ti,ab |
| #105 | 'computed tomogr*':ti,ab |
| #104 | ultrasound:ti,ab OR ultrasonogr*:ti,ab |
| #103 | urinalysis:ti,ab |
| #102 | (electrodermal NEAR/1 (activity OR response)):ti,ab |
| #101 | 'skin conductance response':ti,ab |
| #100 | 'galvanic skin response':ti,ab |
| #99 | cholesterol:ti,ab |
| #98 | 'liver enzyme*':ti,ab |
| #97 | 'lipid profile':ti,ab |
| #96 | 'heart rate':ti,ab |
| #95 | 'personalized risk':ti,ab OR 'personalised risk':ti,ab |
| #94 | 'muscle strength':ti,ab |
| #93 | 'exercise test':ti,ab |
| #92 | ('lung function' NEAR/3 test*):ti,ab |
| #91 | (blood NEAR/3 test*):ti,ab |
| #90 | (genetic NEAR/1 (risk* OR test* OR screen*)):ti,ab |
| #89 | 'genetic susceptibility':ti,ab OR 'genetic predisposition':ti,ab |
| #88 | 'carbon monoxide':ti,ab |
| #87 | 'waist circumference':ti,ab |
| #86 | 'waist hip':ti,ab |
| #85 | 'body composition':ti,ab |
| #84 | 'body measurement*':ti,ab OR anthropom*:ti,ab |
| #83 | 'body mass index':ti,ab |
| #82 | bmi:ti,ab |
| #81 | weight:ti,ab |
| #80 | 'blood pressure*':ti,ab |
| #79 | 'glycated hemoglobin':ti,ab OR 'glycated haemoglobin' OR 'glycosylated hemoglobin':ti,ab OR 'glycosylated haemoglobin':ti,ab |
| #78 | hga1c:ti,ab OR hba1c:ti,ab OR 'hemoglobin a1c':ti,ab OR 'haemoglobin a1c':ti,ab |
| #77 | glucose:ti,ab |
| #76 | ('health status' NEAR/3 indicator*):ti,ab |
| #75 | (modifiable NEAR/3 'risk factor*'):ti,ab |
| #74 | 'health hazard appraisal*':ti,ab |
| #73 | 'risk appraisal*':ti,ab |
| #72 | 'risk assessment':ti,ab |
| #71 | 'risk indicator*':ti,ab |
| #70 | epigenetic*:ti,ab |
| #69 | nutrigenetic*:ti,ab |
| #68 | 'gut microbiome':ti,ab OR 'gut microflora':ti,ab OR 'gut bacteria':ti,ab OR 'gut microbiota':ti,ab |
| #67 | metabolite*:ti,ab |
| #66 | (blood NEAR/3 analyte*):ti,ab |
| #65 | biomarker*:ti,ab |
| #64 | (biological NEAR/3 (data OR information OR metric* OR marker* OR measure* OR indicator* OR risk*)):ti,ab |
| #63 | 'dual energy x ray absorptiometry'/de |
| #62 | 'bone density'/de |
| #61 | 'ketone body'/de |
| #60 | 'ketone'/de |
| #59 | 'metabolic equivalent'/de |
| #58 | 'cotinine'/de |
| #57 | 'plethysmography'/exp |
| #56 | 'electrocardiography'/exp |
| #55 | 'electrooculography'/de |
| #54 | 'ultrasound'/de |
| #53 | 'computer assisted tomography'/de |
| #52 | 'diagnostic imaging'/de |
| #51 | 'alcohol blood level'/de |
| #50 | 'urinalysis'/de |
| #49 | 'electrodermal response'/de |
| #48 | 'nutritional assessment'/de |
| #47 | 'carbon monoxide blood level'/de |
| #46 | 'physical examination'/de |
| #45 | 'blood cell count'/exp |
| #44 | 'blood analysis'/de |
| #43 | 'blood examination'/de |
| #42 | 'heart rate measurement'/de |
| #41 | 'exercise test'/exp |
| #40 | 'disease predisposition'/de |
| #39 | 'blood pressure measurement'/de |
| #38 | 'oximetry'/exp |
| #37 | 'lung function test'/exp |
| #36 | 'diagnostic test'/de |
| #35 | 'personalized medicine'/de |
| #34 | 'blood chemistry'/de |
| #33 | 'anthropometry'/de |
| #32 | 'glycosylated hemoglobin'/exp |
| #31 | 'breath analysis'/exp |
| #30 | 'vital sign'/de |
| #29 | 'breathing rate'/de |
| #28 | 'pulse rate'/de |
| #27 | 'nutritional status'/de |
| #26 | 'muscle strength'/de |
| #25 | 'heart rate'/de |
| #24 | 'chronic disease indicator'/de |
| #23 | 'health status'/de |
| #22 | 'health status indicator'/de |
| #21 | 'genetic screening'/de |
| #20 | 'genetic marker'/de |
| #19 | 'genetic profile'/de |
| #18 | 'genetic susceptibility'/de |
| #17 | 'genetic predisposition'/de |
| #16 | 'intestine flora'/exp |
| #15 | 'cholesterol'/de |
| #14 | 'blood cell'/exp |
| #13 | 'body size'/de |
| #12 | 'waist hip ratio'/de |
| #11 | 'body mass'/de |
| #10 | 'morphometry'/de |
| #9 | 'body composition'/exp |
| #8 | 'blood pressure'/de |
| #7 | 'glucose blood level'/de |
| #6 | 'basal metabolic rate'/de |
| #5 | 'risk assessment'/de |
| #4 | 'body weight'/de |
| #3 | 'biologic monitoring'/de |
| #2 | 'physiologic monitoring'/de |
| #1 | 'biological marker'/de |

[Cochrane Central Register of Controlled Trials](https://www-cochranelibrary-com.ezproxy3.library.arizona.edu/)Issue 5 of 12, May 2021

Date Searched: 6/03/21

Exported PubMed/Embase/CINAHL results (to exclude clinical trial register results)

ID Search

#1 MeSH descriptor: [Biological Phenomena] this term only

#2 MeSH descriptor: [Biomarkers] this term only

#3 MeSH descriptor: [Physiological Phenomena] this term only

#4 MeSH descriptor: [Monitoring, Physiologic] this term only

#5 MeSH descriptor: [Biological Monitoring] this term only

#6 MeSH descriptor: [Body Weight] this term only

#7 MeSH descriptor: [Risk Assessment] this term only

#8 MeSH descriptor: [Basal Metabolism] this term only

#9 MeSH descriptor: [Blood Glucose] this term only

#10 MeSH descriptor: [Blood Pressure] this term only

#11 MeSH descriptor: [Body Composition] explode all trees

#12 MeSH descriptor: [Body Weights and Measures] this term only

#13 MeSH descriptor: [Body Fat Distribution] explode all trees

#14 MeSH descriptor: [Body Mass Index] this term only

#15 MeSH descriptor: [Waist-Hip Ratio] this term only

#16 MeSH descriptor: [Body Size] explode all trees

#17 MeSH descriptor: [Blood Cells] explode all trees

#18 MeSH descriptor: [] explode all trees and with qualifier(s): [blood - BL]

#19 MeSH descriptor: [] explode all trees and with qualifier(s): [urine - UR]

#20 MeSH descriptor: [Cholesterol] this term only

#21 MeSH descriptor: [Gastrointestinal Microbiome] this term only

#22 MeSH descriptor: [Genetic Predisposition to Disease] this term only

#23 MeSH descriptor: [Genetic Profile] this term only

#24 MeSH descriptor: [Genetic Markers] this term only

#25 MeSH descriptor: [Genetic Carrier Screening] this term only

#26 MeSH descriptor: [Genetic Testing] this term only

#27 MeSH descriptor: [Health Status Indicators] this term only

#28 MeSH descriptor: [Health Status] this term only

#29 MeSH descriptor: [Heart Rate] this term only

#30 MeSH descriptor: [Muscle Strength] explode all trees

#31 MeSH descriptor: [Nutritional Status] this term only

#32 MeSH descriptor: [Pulse] this term only

#33 MeSH descriptor: [Respiratory Rate] this term only

#34 MeSH descriptor: [Vital Signs] this term only

#35 MeSH descriptor: [Breath Tests] this term only

#36 MeSH descriptor: [Glycated Hemoglobin A] this term only

#37 MeSH descriptor: [Anthropometry] this term only

#38 MeSH descriptor: [Blood Chemical Analysis] this term only

#39 MeSH descriptor: [Health Impact Assessment] this term only

#40 MeSH descriptor: [Precision Medicine] this term only

#41 MeSH descriptor: [Diagnostic Tests, Routine] this term only

#42 MeSH descriptor: [Respiratory Function Tests] explode all trees

#43 MeSH descriptor: [Spirometry] explode all trees

#44 MeSH descriptor: [Oximetry] explode all trees

#45 MeSH descriptor: [Blood Pressure Determination] this term only

#46 MeSH descriptor: [Direct-To-Consumer Screening and Testing] this term only

#47 MeSH descriptor: [Disease Susceptibility] explode all trees

#48 MeSH descriptor: [Exercise Test] explode all trees

#49 MeSH descriptor: [Heart Rate Determination] this term only

#50 MeSH descriptor: [Hematologic Tests] this term only

#51 MeSH descriptor: [Blood Cell Count] explode all trees

#52 MeSH descriptor: [Physical Examination] this term only

#53 MeSH descriptor: [Carbon Monoxide] this term only

#54 MeSH descriptor: [Nutritional Physiological Phenomena] this term only

#55 MeSH descriptor: [Nutrition Assessment] this term only

#56 MeSH descriptor: [Galvanic Skin Response] this term only

#57 MeSH descriptor: [Urinalysis] this term only

#58 MeSH descriptor: [Ethanol] this term only

#59 MeSH descriptor: [Blood Alcohol Content] this term only

#60 MeSH descriptor: [Diagnostic Imaging] explode all trees

#61 MeSH descriptor: [] explode all trees and with qualifier(s): [diagnostic imaging - DG]

#62 MeSH descriptor: [Electrooculography] this term only

#63 MeSH descriptor: [Electrocardiography] explode all trees

#64 MeSH descriptor: [Plethysmography] explode all trees

#65 MeSH descriptor: [Cotinine] this term only

#66 MeSH descriptor: [Metabolic Equivalent] this term only

#67 MeSH descriptor: [Ketones] this term only

#68 MeSH descriptor: [Ketone Bodies] this term only

#69 MeSH descriptor: [Bone Density] this term only

#70 MeSH descriptor: [Absorptiometry, Photon] this term only

#71 MeSH descriptor: [Electric Impedance] this term only

#72 (biological NEAR/3 (data OR information OR metric* OR marker* OR measure* OR indicator* OR risk*)):ti,ab

#73 biomarker*:ti,ab

#74 (blood NEAR/3 analyte*):ti,ab

#75 metabolite*:ti,ab

#76 ("gut microbiome" OR "gut microflora" OR "gut bacteria" OR "gut microbiota"):ti,ab

#77 nutrigenetic*:ti,ab

#78 epigenetic*:ti,ab

#79 "risk indicator":ti,ab OR "risk indicators":ti,ab

#80 "risk appraisal":ti,ab or "risk appraisals":ti,ab

#81 "health hazard appraisal":ti,ab OR "health hazard appraisals":ti,ab

#82 (modifiable NEAR/3 "risk factor"):ti,ab OR (modifiable NEAR/3 "risk factors"):ti,ab

#83 ('health status' NEAR/3 indicator*):ti,ab

#84 glucose:ti,ab

#85 hga1c:ti,ab OR hba1c:ti,ab OR "hemoglobin A1c":ti,ab OR "haemoglobin A1c":ti,ab

#86 "glycated hemoglobin":ti,ab OR "glycated haemoglobin" OR 'glycosylated hemoglobin':ti,ab OR 'glycosylated haemoglobin':ti,ab

#87 anthropom*:ti,ab

#88 'blood pressure':ti,ab or "blood pressures":ti,ab

#89 weight:ti,ab

#90 bmi:ti,ab

#91 "body mass index":ti,ab

#92 "body measurement":ti,ab OR "body measurements":ti,ab

#93 "body composition":ti,ab

#94 'waist hip':ti,ab

#95 'waist circumference':ti,ab

#96 'carbon monoxide':ti,ab

#97 'genetic susceptibility':ti,ab OR "genetic predisposition":ti,ab

#98 (genetic NEAR/1 (risk* OR test* OR screen*)):ti,ab

#99 (blood NEAR/3 test*):ti,ab

#100 ("lung function" NEAR/3 test*):ti,ab

#101 "exercise test":ti,ab

#102 "muscle strength":ti,ab

#103 'personalized risk':ti,ab OR 'personalised risk':ti,ab

#104 'heart rate':ti,ab

#105 'lipid profile':ti,ab

#106 'liver enzyme':ti,ab OR "liver enzymes":ti,ab

#107 cholesterol:ti,ab

#108 'galvanic skin response':ti,ab

#109 'skin conductance response':ti,ab

#110 (electrodermal NEAR/1 (activity OR response)):ti,ab

#111 urinalysis:ti,ab

#112 (ultrasound OR ultrasonogr*):ti,ab

#113 "computed tomography":ti,ab OR "computed tomograph":ti,ab

#114 electrocardiogra*:ti,ab OR electrokardiogra*:ti,ab

#115 (EKG or ECG):ti,ab

#116 plethysmography:ti,ab

#117 cotinine:ti,ab

#118 spirometry:ti,ab

#119 ("breath test" or "breath tests"):ti,ab

#120 "risk assessment":ti,ab

#121 ("precision nutrition" OR "precision medicine"):ti,ab

#122 electrooculography:ti,ab

#123 (ethnaol OR "blood alcohol"):ti,ab

#124 ("pulmonary function test" OR "pulmonary function tests"):ti,ab

#125 ketone*:ti,ab

#126 ("beta-hydroxybutyrate" or "3-hydroxybutyric acid"):ti,ab

#127 ("bone density" OR "dexa scan" OR "dxa scan"):ti,ab

#128 "dual energy x-ray absorptiometry":ti,ab

#129 bodpod:ti,ab or bod-pod:ti,ab

#130 ("underwater weight" OR "underwater weighing"):ti,ab

#131 ("hydrostatic weight" OR "hydrostatic weighing"):ti,ab

#132 hydrodensitometry:ti,ab

#133 {OR #1-#132}

#134 MeSH descriptor: [Feedback] this term only

#135 MeSH descriptor: [Feedback, Psychological] this term only

#136 MeSH descriptor: [Communication] this term only

#137 MeSH descriptor: [Health Communication] this term only

#138 MeSH descriptor: [Health Education] this term only

#139 MeSH descriptor: [Patient Education as Topic] this term only

#140 MeSH descriptor: [Cues] this term only

#141 MeSH descriptor: [Genetic Counseling] this term only

#142 MeSH descriptor: [Counseling] this term only

#143 MeSH descriptor: [Motivational Interviewing] this term only

#144 MeSH descriptor: [Correspondence as Topic] explode all trees

#145 MeSH descriptor: [Behavior Therapy] this term only

#146 [mh ^Lifestyle] AND [mh /TH]

#147 MeSH descriptor: [Wearable Electronic Devices] this term only

#148 MeSH descriptor: [Monitoring, Ambulatory] explode all trees

#149 MeSH descriptor: [Self-Testing] explode all trees

#150 MeSH descriptor: [Telephone] this term only

#151 MeSH descriptor: [Cell Phone] explode all trees

#152 feedback*:ti,ab

#153 ((communicat* OR provide*) NEAR/3 (results OR information OR risk*)):ti,ab

#154 'cue to action':ti,ab or "cues to action":ti,ab

#155 ((tailored OR personalized OR personalised OR individualized OR individualised) NEAR/5 (communicat* OR information)):ti,ab

#156 (receiv* NEAR/3 (results OR information)):ti,ab

#157 ((knowledge or knowing) NEAR/3 result*):ti,ab

#158 counsel*:ti,ab

#159 coach*:ti,ab

#160 advice:ti,ab

#161 "motivational interviewing":ti,ab

#162 ((behavior or behaviour) NEAR/1 (therap* OR intervention*)):ti,ab

#163 ((tailored OR personalized OR personalised OR individualized OR individualised) NEAR/3 educat*):ti,ab

#164 (education* NEAR/3 (module OR support OR session)):ti,ab

#165 (educate NEAR/3 (individual* OR patient*)):ti,ab

#166 (received NEAR/3 education*):ti,ab

#167 ((patient or health) NEAR/1 education):ti,ab

#168 wearable*:ti,ab

#169 biosensor*:ti,ab

#170 ("sensor technology" OR "sensor technologies"):ti,ab

#171 (device NEAR/3 (monitor* OR track*)):ti,ab

#172 ("smart device" OR "smart devices"):ti,ab

#173 (self NEAR/3 (monitor* OR test*)):ti,ab

#174 ("ambulatory monitor" OR "ambulatory monitors"):ti,ab

#175 (("personal health" OR personalized health" OR personalised health") NEAR/3 monitor*):ti,ab

#176 messag*:ti,ab

#177 (telephone* or cellphone* or cell-phone or cell-phones or smartphone* or smart-phone or smart-phones or "mobile phone" or "mobile phones"):ti,ab

#178 ((month* or week*) NEAR/3 support):ti,ab

#179 ((interpreted or reported or informed) NEAR/3 (measurement* or result* or data or level*)):ti,ab

#180 {OR #134-#179}

#181 #133 AND #180

#182 MeSH descriptor: [Blood Glucose Self-Monitoring] this term only

#183 MeSH descriptor: [Blood Pressure Monitoring, Ambulatory] this term only

#184 MeSH descriptor: [Blood Pressure Monitors] this term only

#185 ((home OR self OR continuous OR ambulatory) NEAR/3 (("blood pressure" NEXT monitor*) OR (glucose NEXT monitor*))):ti,ab

#186 ("flash glucose monitor" OR "flash glucose monitors"):ti,ab

#187 ("hr monitor" OR "hr monitors" OR "heart rate monitor" OR "heart rate monitors"):ti,ab

#188 "physiological feedback":ti,ab

#189 "biological feedback":ti,ab

#190 biofeedback:ti,ab

#191 (self-weigh or self-weighing):ti,ab

#192 ("daily weight" OR "daily weights" OR "daily weighing"):ti,ab

#193 (connected NEAR/1 (glucometer* OR scale*)):ti,ab

#194 ("smart scale" OR "smart scales"):ti,ab

#195 (BIA NEAR/3 scale*):ti,ab

#196 ((“bioelectric impedance” OR “bioelectrical impedance” OR “bio electric impedance” OR “bio electrical impedance”) NEAR/3 (scale* OR analysis)):ti,ab OR ((bioimpedance OR “bio impedance”) NEAR/3 (scale* OR analysis)):ti,ab

#197 {OR #182-#196}

#198 #181 OR #197

#199 MeSH descriptor: [Behavior] this term only

#200 MeSH descriptor: [Health Behavior] explode all trees

#201 MeSH descriptor: [Behavior Control] this term only

#202 MeSH descriptor: [Behavioral Medicine] this term only

#203 MeSH descriptor: [Behavioral Research] this term only

#204 MeSH descriptor: [Feeding Behavior] this term only

#205 MeSH descriptor: [Health Knowledge, Attitudes, Practice] this term only

#206 MeSH descriptor: [Healthy Lifestyle] explode all trees

#207 MeSH descriptor: [Health Promotion] explode all trees

#208 MeSH descriptor: [Motivation] explode all trees

#209 MeSH descriptor: [Risk Reduction Behavior] this term only

#210 MeSH descriptor: [Self Efficacy] this term only

#211 MeSH descriptor: [Self Care] this term only

#212 MeSH descriptor: [Self-Management] this term only

#213 MeSH descriptor: [Awareness] this term only

#214 MeSH descriptor: [Inhibition, Psychological] explode all trees

#215 MeSH descriptor: [Treatment Adherence and Compliance] this term only

#216 MeSH descriptor: [Patient Compliance] this term only

#217 MeSH descriptor: [Patient Participation] this term only

#218 MeSH descriptor: [Public Health] this term only

#219 MeSH descriptor: [Public Health Practice] this term only

#220 MeSH descriptor: [Preventive Medicine] this term only

#221 MeSH descriptor: [] explode all trees and with qualifier(s): [prevention & control - PC]

#222 MeSH descriptor: [Preventive Health Services] this term only

#223 MeSH descriptor: [Primary Prevention] explode all trees

#224 MeSH descriptor: [Secondary Prevention] this term only

#225 MeSH descriptor: [Tertiary Prevention] this term only

#226 MeSH descriptor: [Smoking Prevention] this term only

#227 MeSH descriptor: [Harm Reduction] this term only

#228 [mh ^"Treatment Outcome"] AND ([mh /PX] OR [mh ^Lifestyle])

#229 ((behavior* OR behaviour* OR lifestyle) NEAR/3 (change* OR modif* or promot*)):ti,ab

#230 (health NEXT (behavior* OR behaviour*)):ti,ab

#231 ("healthy lifestyle" or "healthy lifestyles"):ti,ab

#232 (self NEAR/3 (care OR management or efficacy)):ti,ab

#233 awareness:ti,ab

#234 ((risk or harm or "sedentary behavior" OR "sedentary behaviour") NEAR/3 reduc*):ti,ab

#235 "weight loss":ti,ab

#236 "weight control":ti,ab

#237 (smok* NEAR/3 (behavior* OR behaviour* OR cessation OR quit*)):ti,ab

#238 (self NEXT regulat*):ti,ab

#239 motivated:ti,ab or motivation:ti,ab

#240 adherence:ti,ab or compliance:ti,ab

#241 prevention:ti,ab OR preventive:ti,ab

#242 "health promotion":ti,ab

#243 (improv* NEAR/3 (activit* OR eating OR diet* OR health OR fitness)):ti,ab

#244 ((exercise OR "physical activity" or diet* or eating or weight) NEAR/3 (change OR behavior* OR behaviour* OR modif* or maint* or motivat* or promot*)):ti,ab

#245 "public health":ti,ab

#246 {OR #199-#245}

#247 #198 AND #246 in Trials

EBSCOhost PsycINFO
Date Searched: 6/4/21

| # | Query |
| --- | --- |
| S190 | S185 AND S189 |
| S189 | S186 OR S187 OR S188 |
| S188 | TI trial OR AB trial |
| S187 | TI random* OR AB random* |
| S186 | (DE "Randomized Controlled Trials") OR (DE "Clinical Trials") |
| S185 | S146 AND S184 |
| S184 | S147 OR S148 OR S149 OR S150 OR S151 OR S152 OR S153 OR S154 OR S155 OR S156 OR S157 OR S158 OR S159 OR S160 OR S161 OR S162 OR S163 OR S164 OR S165 OR S166 OR S167 OR S168 OR S169 OR S170 OR S171 OR S172 OR S173 OR S174 OR S175 OR S176 OR S177 OR S178 OR S179 OR S180 OR S181 OR S182 OR S183 |
| S183 | TI "public health" OR AB "public health" |
| S182 | TI ((exercise OR "physical activity" OR diet* OR eating OR weight) N3 (behavio#r* OR chang* OR maint* OR motivat* OR promot* OR modif*)) OR AB ((exercise OR "physical activity" OR diet* OR eating OR weight) N3 (behavio#r* OR chang* OR maint* OR motivat* OR promot* OR modif*)) |
| S181 | TI ( improv* N3 (activit* OR eating OR diet* OR health OR fitness) ) OR AB ( improv* N3 (activit* OR eating OR diet* OR health OR fitness) ) |
| S180 | TI ( prevention or preventive ) OR AB ( prevention or preventive ) |
| S179 | TI ( adherence or compliance ) OR AB ( adherence or compliance ) |
| S178 | TI (motivated OR motivation) OR AB (motivated OR motivation) |
| S177 | TI "self regulat*" OR AB "self regulat*" |
| S176 | TI (smok* N3 (behavio#r* OR cessation or quit*)) OR AB (smok* N3 (behavio#r* OR cessation or quit*)) |
| S175 | TI "weight control" OR AB "weight control" |
| S174 | TI "weight loss" OR AB "weight loss" |
| S173 | TI ((risk or harm or "sedentary behavio#r") N3 reduc*) OR AB ((risk or harm or "sedentary behavio#r") N3 reduc*) |
| S172 | TI awareness OR AB awareness |
| S171 | TI "healthy lifestyle" OR AB "healthy lifestyle" |
| S170 | TI "health behavio#r*" OR AB "health behavio#r*" |
| S169 | TI ( self N3 (care or management or efficacy) ) OR AB ( self N3 (care or management or efficacy) ) |
| S168 | TI ( (behavio#r* OR lifestyle) N3 (chang* or modif* or promot*) ) OR AB ( (behavio#r* OR lifestyle) N3 (chang* or modif* or promot*) ) |
| S167 | (DE "Treatment Outcomes") OR (DE "Health Outcomes") |
| S166 | DE "Harm Reduction" |
| S165 | DE "Substance Use Prevention" OR DE "Relapse Prevention" |
| S164 | DE "Prevention" |
| S163 | DE "Public Health Services" |
| S162 | (DE "Public Health") |
| S161 | DE "Compliance" |
| S160 | DE "Treatment Compliance" |
| S159 | (DE "Awareness") OR (DE "Health Awareness") |
| S158 | DE "Self-Management" |
| S157 | DE "Self-Care" |
| S156 | DE "Self-Efficacy" |
| S155 | DE "Health Behavior Measures" |
| S154 | DE "Motivation" OR DE "Goals" OR DE "Incentives" |
| S153 | DE "Health Promotion" |
| S152 | DE "Lifestyle Changes" |
| S151 | DE "Health Attitudes" |
| S150 | DE "Behavior Modification" |
| S149 | DE "Behavioral Medicine" |
| S148 | DE "Health Behavior" OR DE "Health Risk Behavior" OR DE "Preventive Health Behavior" |
| S147 | DE "Behavior" |
| S146 | S131 OR S145 |
| S145 | (S132 OR S133 OR S134 OR S135 OR S136 OR S137 OR S138 OR S139 OR S140 OR S141 OR S142 OR S143 OR S144) |
| S144 | TI ((bio#electric impedance" OR "bio#electrical impedance") N3 (scale* OR analysis)) OR AB ((bio#electric impedance" OR "bio#electrical impedance") N3 (scale* OR analysis)) OR TI (bio#impedance N3 (scale* OR analysis)) AB (bio#impedance N3 (scale* OR analysis)) |
| S143 | TI (BIA N3 scale*) OR AB (BIA N3 scale*) |
| S142 | TI "smart scale*"OR AB "smart scale*" |
| S141 | TI (connected N1 (glucometer* OR scale*)) OR AB (connected N1 (glucometer* OR scale*)) |
| S140 | TI "daily weigh*" OR AB "daily weigh*" |
| S139 | TI self-weigh* OR AB self-weigh* |
| S138 | TI ("hr monitor*" OR "heart rate monitor*") OR AB ("hr monitor*" OR "heart rate monitor*") |
| S137 | TI "flash glucose monitor*" OR AB "flash glucose monitor*" |
| S136 | TI biofeedback OR AB biofeedback |
| S135 | TI "biological feedback" OR AB "biological feedback" |
| S134 | TI "physiological feedback" OR AB "physiological feedback" |
| S133 | TI ( (home or self or continuous OR ambulatory) N3 ("blood pressure monitor*" OR "glucose monitor*") ) OR AB ( (home or self or continuous OR ambulatory) N3 ("blood pressure monitor*" OR "glucose monitor*") ) |
| S132 | DE "Biofeedback" |
| S131 | (S89 AND S130) |
| S130 | (S90 OR S91 OR S92 OR S93 OR S94 OR S95 OR S96 OR S97 OR S98 OR S99 OR S100 OR S101 OR S102 OR S103 OR S104 OR S105 OR S106 OR S107 OR S108 OR S109 OR S110 OR S111 OR S112 OR S113 OR S114 OR S115 OR S116 OR S117 OR S118 OR S119 OR S120 OR S121 OR S122 OR S123 OR S124 OR S125 OR S126 OR S127 OR S128 OR S129) |
| S129 | TI ((interpreted OR reported OR informed) N3 (measurement* OR result* OR data or level*)) OR AB ((interpreted OR reported OR informed) N3 (measurement* OR result* OR data or level*)) |
| S128 | TI ((month* or week*) N3 support) OR AB ((month* or week*) N3 support) |
| S127 | TI (telephone* OR cellphone* OR cell-phone* OR smartphone* OR smart-phone* OR "mobile phone*") OR AB (telephone* OR cellphone* OR cell-phone* OR smartphone* OR smart-phone* OR "mobile phone*") |
| S126 | TI messag* OR AB messag* |
| S125 | TI ("personal* health" N3 monitor*) OR AB ("personal* health" N3 monitor*) |
| S124 | TI "ambulatory monitor*" OR AB "ambulatory monitor*" |
| S123 | TI (self N3 (monitor* OR test*)) OR AB (self N3 (monitor* OR test*)) |
| S122 | TI "smart device*" OR AB "smart device*" |
| S121 | TI (device N3 (monitor* OR track*)) OR AB (device N3 (monitor* OR track*)) |
| S120 | TI "sensor technolog*" OR AB "sensor technolog*" |
| S119 | TI biosensor* OR AB biosensor* |
| S118 | TI wearable* OR AB wearable* |
| S117 | TI (behavio#r N1 (therap* OR intervention*)) OR AB (behavio#r N1 (therap* OR intervention*)) |
| S116 | TI "motivational interviewing" OR AB motivational interviewing" |
| S115 | TI advice OR AB advice |
| S114 | TI coach* OR AB coach* |
| S113 | TI ( (knowledge or knowing) N3 result* ) OR AB ( (knowledge or knowing) N3 result* ) |
| S112 | TI (received N3 education*) OR AB (received N3 education*) OR TI (educate N3 (individual* OR patient*)) OR AB (educate N3 (individual* OR patient*)) OR TI ((patient or health) N1 education) OR AB ((patient or health N1 education) |
| S111 | TI ( education* N3 (module OR support OR session) ) OR AB ( education* N3 (module OR support OR session) ) |
| S110 | TI ( (tailored OR personali?ed or individuali?ed) N3 educat* ) OR AB ( (tailored OR personali?ed or individuali?ed) N3 educat* ) |
| S109 | TI counsel* OR AB counsel* |
| S108 | TI ( receiv* N3 (results or information) ) OR AB ( receiv* N3 (results or information) ) |
| S107 | TI ( (tailored OR personali?ed or individuali?ed) N5 (communicat* OR information) ) OR AB ( (tailored OR personali?ed or individuali?ed) N5 (communicat* OR information) ) |
| S106 | TI ("cue to action" OR "cues to action") OR AB ("cue to action" OR "cues to action") |
| S105 | TI ( (communicat* OR provide*) N3 (results OR information OR risk*) ) OR AB ( (communicat* OR provide*) N3 (results OR information OR risk*) ) |
| S104 | TI feedback* OR AB feedback* |
| S103 | DE "Smartphones" OR DE "Smartphone Use" OR DE "Text Messaging" |
| S102 | DE "Hot Line Services" OR DE "Telephone Systems" OR DE "Mobile Phones" OR DE" Mobile Applications" |
| S101 | DE "Self-Monitoring" |
| S100 | DE "Wearable Devices" |
| S99 | DE "Behavior Therapy" |
| S98 | DE "Communication Systems" |
| S97 | DE "Motivational Interviewing" |
| S96 | DE "Counseling" |
| S95 | DE "Genetic Counseling" |
| S94 | DE "Cues" |
| S93 | DE "Client Education" |
| S92 | DE "Health Education" |
| S91 | DE "Communication" |
| S90 | DE "Feedback" OR DE "Knowledge of Results" |
| S89 | (S1 OR S2 OR S3 OR S4 OR S5 OR S6 OR S7 OR S8 OR S9 OR S10 OR S11 OR S12 OR S13 OR S14 OR S15 OR S16 OR S17 OR S18 OR S19 OR S20 OR S21 OR S22 OR S23 OR S24 OR S25 OR S26 OR S27 OR S28 OR S29 OR S30 OR S31 OR S32 OR S33 OR S34 OR S35 OR S36 OR S37 OR S38 OR S39 OR S40 OR S41 OR S42 OR S43 OR S44 OR S45 OR S46 OR S47 OR S48 OR S49 OR S50 OR S51 OR S52 OR S53 OR S54 OR S55 OR S56 OR S57 OR S58 OR S59 OR S60 OR S61 OR S62 OR S63 OR S64 OR S65 OR S66 OR S67 OR S68 OR S69 OR S70 OR S71 OR S72 OR S73 OR S74 OR S75 OR S76 OR S77 OR S78 OR S79 OR S80 OR S81 OR S82 OR S83 OR S84 OR S85 OR S86 OR S87 OR S88 ) |
| S88 | TI (hydrodensitometry) OR AB (hydrodensitometry) |
| S87 | TI ("hydrostatic weigh*") OR AB ("hydrostatic weigh*") |
| S86 | TI ("underwater weigh*") OR AB ("underwater weigh*") |
| S85 | TI (bod-pod OR bodpod) OR AB (bod-pod OR bodpod) |
| S84 | TI ("bone density" OR "dexa scan" OR "dxa scan" OR "dual energy x-ray absorptiometry") OR AB ("bone density" OR "dexa scan" OR "dxa scan" OR "dual energy x-ray absorptiometry") |
| S83 | TI ("beta-hydroxybutyrate" OR "3-hydroxybutyric acid") OR AB ("beta-hydroxybutyrate" OR "3-hydroxybutyric acid") |
| S82 | TI (ketone*) OR AB (ketone*) |
| S81 | TI ("pulmonary function test*") OR AB ("pulmonary function test*") |
| S80 | TI (ethanol or "blood alcohol") OR AB (ethanol or "blood alcohol") |
| S79 | TI (electrooculography) OR AB (electrooculography) |
| S78 | TI ("precision nutrition" OR "precision medicine") OR AB ("precision nutrition" OR "precision medicine") |
| S77 | TI ("risk assessment") OR AB ("risk assessment") OR TI ("risk appraisal*") OR AB ("risk appraisal*") OR TI ("health hazard appraisal*") OR AB ("health hazard appraisal*") |
| S76 | TI ("breath test*") OR AB ("breath test*") |
| S75 | TI (spirometry) OR AB (spirometry) |
| S74 | TI (cotinine) OR AB (cotinine) |
| S73 | TI (plethysmography) OR AB (plethysmography) |
| S72 | TI (ekg OR ecg) OR AB (ekg or ecg) |
| S71 | TI (electrocardiogra* OR electrokardiogra*) OR AB (electrocardiogra* OR electrokardiogra*) |
| S70 | TI ("computed tomogr*") OR AB (computed tomogr*") |
| S69 | TI (ultrasound OR ultrasonogr*) OR AB (ultrasound OR ultrasonogr*) |
| S68 | TI ( urinalysis) OR AB (urinalysis) |
| S67 | TI ( electrodermal N1 (activity or response) ) OR AB ( electrodermal N1 (activity or response) ) |
| S66 | TI "skin conductance response" OR AB "skin conductance response" |
| S65 | TI "galvanic skin response" OR AB "galvanic skin response" |
| S64 | TI "liver enzyme*" OR AB "liver enzyme*" |
| S63 | TI cholesterol OR AB cholesterol |
| S62 | TI "lipid profile" OR AB "lipid profile" |
| S61 | TI "heart rate" OR AB "heart rate" |
| S60 | TI "personali?ed risk" OR AB "personali?ed risk" |
| S59 | TI (blood N3 test*) OR AB (blood N3 test*) OR TI ("lung function" N3 test*) OR AB ("lung function" N3 test*) OR TI "exercise test*" OR AB "exercise test*" OR TI "muscle strength" OR AB "muscle strength" |
| S58 | TI ( (genetic N1 (risk* OR test* OR screen*)) ) OR AB ( (genetic N1 (risk* OR test* OR screen*)) ) |
| S57 | TI "genetic predisposition" OR AB "genetic predisposition" |
| S56 | TI "genetic susceptibility" OR AB "genetic susceptibility" |
| S55 | TI "carbon monoxide" OR AB "carbon monoxide" |
| S54 | TI "waist circumference" OR AB "waist circumference" |
| S53 | TI "waist hip" OR AB "waist hip" |
| S52 | TI "body composition" OR AB "body composition" |
| S51 | TI "body measurement*" OR AB "body measurement*" OR TI anthropom* OR AB anthropom* |
| S50 | TI "body mass index" OR AB "body mass index" |
| S49 | TI BMI OR AB BMI |
| S48 | TI weight OR AB weight |
| S47 | TI "blood pressure*" OR AB "blood pressure*" |
| S46 | TI "glycated h#emoglobin" OR AB "glycated h#emoglobin" OR TI "glycosylated h#emoglobin" OR AB "glycosylated h#emoglobin" |
| S45 | TI ( HgA1c OR HbA1c ) OR AB ( HgA1c OR HbA1c ) OR TI ( "h#emoglobin A1c" ) OR AB ( "h#emoglobin A1c" ) |
| S44 | TI glucose OR AB glucose |
| S43 | TI "health status" N3 indicator* OR AB "health status" N3 indicator* |
| S42 | TI modifiable N3 "risk factor*" OR AB modifiable N3 "risk factor*" |
| S41 | TI "risk indicator*" OR AB "risk indicator*" |
| S40 | TI epigenetic* OR AB epigenetic* |
| S39 | TI nutrigenetic* OR AB nutrigenetic* |
| S38 | TI "gut microbiome*" OR AB "gut microbiome*" OR TI "gut microflora" OR AB "gut microflora" OR TI "gut bacteria" OR AB "gut bacteria" OR TI "gut microbiota" OR AB "gut microbiota" |
| S37 | TI metabolite* OR AB metabolite* |
| S36 | TI blood N3 analyte* OR AB blood N3 analyte* |
| S35 | TI biomarker* OR AB biomarker* |
| S34 | ( TI (biological N3 (data or information or metric* OR marker* OR measure*OR indicator* OR risk*)) ) OR ( AB (biological N3 (data or information or metric* OR marker* OR measure* OR indicator* OR risk*)) ) |
| S33 | DE "Metabolic Rates" |
| S32 | DE "Plethysmography" OR DE "Electroplethysmography" |
| S31 | DE "Electrocardiography" |
| S30 | DE "Computer Assisted Diagnosis" OR DE "Tomography" |
| S29 | DE "Blood Alcohol Concentration" |
| S28 | DE "Urinalysis" |
| S27 | DE "Galvanic Skin Response" |
| S26 | DE "Carbon Monoxide" |
| S25 | DE "Physical Examination" |
| S24 | DE "Precision Medicine" |
| S23 | DE "Blood Plasma" |
| S22 | DE "Anthropometry" |
| S21 | DE "Respiration" |
| S20 | DE "Arterial Pulse" |
| S19 | DE "Nutrition" |
| S18 | DE "Physical Strength" OR DE "Muscle Tone" |
| S17 | DE "Heart Rate" OR DE "Heart Rate Variability" |
| S16 | DE "Health Status" |
| S15 | DE "Genetic Testing" |
| S14 | DE "Cholesterol" |
| S13 | DE "Blood Serum" |
| S12 | DE "Blood Cells" OR DE "Erythrocytes" OR DE "Leucocytes" |
| S11 | DE "Body Size" |
| S10 | DE "Body Mass Index" |
| S9 | DE "Body Fat" |
| S8 | DE "Blood Pressure" OR DE "Diastolic Pressure" OR DE "Systolic Pressure" |
| S7 | DE "Blood Sugar" |
| S6 | DE "Basal Metabolism" |
| S5 | DE "Risk Assessment" |
| S4 | DE "Body Weight" |
| S3 | DE "Physiological Processes" |
| S2 | DE "Biological Markers" |
| S1 | DE "Biological Processes" |

ProQuest Dissertations & Theses Global

Date Searched: 6/11/21

((((Exact("risk assessment" OR "physical examinations" OR "health risk assessment" OR "urine" OR "blood tests" OR "ethanol" OR "hemoglobin" OR "electrocardiography" OR "body composition" OR "cholesterol" OR "breath tests" OR "blood pressure" OR "glucose" OR "heart rate" OR "nutritional status" OR "vital signs" OR "diagnostic tests" OR "biomarkers" OR "body mass index" OR "bone density" OR "carbon monoxide" OR "weight" OR "blood" OR "muscle strength" OR "urinalysis" OR "diagnostics" OR "spirometry" OR "genetic testing" OR "precision medicine" OR "genetic markers" OR "medical imaging" OR "body fat") OR ti(hydrodensitometry) OR ab(hydrodensitometry) OR ti(((hydrostatic or underwater) p/0 weigh*)) OR ab(((hydrostatic or underwater) p/0 weigh*)) OR ti(bod-pod OR bodpod) OR ab(bod-pod OR bodpod) OR ti("bone density" OR "dexa scan" OR "dxa scan" OR "dual energy x-ray absorptiometry") OR ab("bone density" OR "dexa scan" OR "dxa scan" OR "dual energy x-ray absorptiometry") OR ti("pulmonary function test*") OR ab("pulmonary function test*") OR ti(ethanol or "blood alcohol") OR ab(ethanol or "blood alcohol") OR ti(electrooculography) OR ab(electrooculography) OR ti("precision nutrition" OR "precision medicine") OR ab("precision nutrition" OR "precision medicine") OR ti("risk assessment") OR ab("risk assessment") OR ti(("breath test" OR "breath testing" OR "breath tests")) OR ab(("breath test" OR "breath testing" OR "breath tests")) OR ti(spirometry) OR ab(spirometry) OR ti(cotinine) OR ab(cotinine) OR ti(plethysmography) OR ab(plethysmography) OR ti(ekg or ecg) OR ab(ekg or ecg) OR ti((electrocardiogra* OR electrokardiogra*) ) OR ab((electrocardiogra* OR electrokardiogra*) ) OR ti("computed tomogr*) OR ab("computed tomogr*) OR ti((ultrasound OR ultrasonogr*)) OR ab((ultrasound OR ultrasonogr*)) OR ti(urinalysis) OR ab(urinalysis) OR ti(electrodermal NEAR/1 (activity or response)) OR ab(electrodermal NEAR/1 (activity or response)) OR ti("skin conductance response") OR ab("skin conductance response") OR ti("galvanic skin response") OR ab("galvanic skin response") OR ti(("liver enzyme" OR "liver enzymes")) OR ab(("liver enzyme" OR "liver enzymes")) OR ti(cholesterol) OR ab(cholesterol) OR ti("lipid profile") OR ab("lipid profile") OR ti("heart rate") OR ab("heart rate") OR ti((personalised or personalized) p/0 risk) OR ab((personalised or personalized) p/0 risk) OR ti("genetic predisposition") OR ab("genetic predisposition") OR ti("genetic susceptibility") OR ab("genetic susceptibility") OR ti("carbon monoxide") OR ab("carbon monoxide") OR ti("waist circumference") OR ab("waist circumference") OR ti("waist hip") OR ab("waist hip") OR ti("body composition") OR ab("body composition") OR ti(("body measurement" OR "body measurements")) OR ab(("body measurement" OR "body measurements")) OR ti("body mass index") OR ab("body mass index") OR ti(BMI) OR ab(BMI) OR ti(weight) OR ab(weight) OR ti(blood p/0 pressure) OR ab(blood p/0 pressure) OR ti(glycated p/0 (hemoglobin or haemoglobin) OR glycosylated p/0 (hemoglobin or haemoglobin)) OR ab(glycated p/0 (hemoglobin or haemoglobin) OR glycosylated p/0 (hemoglobin or haemoglobin)) OR ti(HgA1c OR HbA1c OR (hemoglobin or haemoglobin) p/0 A1c) OR ab(HgA1c OR HbA1c OR (hemoglobin or haemoglobin) p/0 A1c) OR ti(glucose) OR ab(glucose) OR ti("health status" NEAR/3 indicator) OR ab( "health status" NEAR/3 indicator) OR ti( modifiable NEAR/3 risk p/0 factor ) OR ab( modifiable NEAR/3 risk p/0 factor ) OR ti(risk p/0 indicator) OR ab(risk p/0 indicator) OR ti(epigenetic*) OR ab(epigenetic*) OR ti(nutrigenetic*) OR ab(nutrigenetic*) OR TI(gut p/0 microbiome) OR AB(gut p/0 microbiome) OR TI("gut microflora") OR AB("gut microflora") OR TI("gut bacteria") OR AB("gut bacteria") OR TI("gut microbiota" )OR AB("gut microbiota") OR ti(metabolite) OR ab(metabolite) OR ti(blood NEAR/3 analyte) OR ab(blood NEAR/3 analyte) OR ti(biomarker) OR ab(biomarker) OR ti(biological NEAR/3 (data OR information OR metric OR marker OR measure OR indicator OR risk)) OR ab(biological NEAR/3 (data OR information OR metric OR marker OR measure OR indicator OR risk))) AND (Exact("feedback") OR Exact("interpersonal communication" OR "communications systems" OR "communication") OR Exact("health education" OR "public health education" OR "patient education") OR Exact("cues/cueing") OR Exact("genetic counseling" OR "dietetic counseling" OR "counseling psychology" OR "counseling" OR "counseling services" OR "counseling education") OR Exact("text messaging" OR "mobile phones" OR "telephones" OR "smartphones" OR "telephone hotlines") OR ti(feedback) OR ab(feedback) OR ti((communicat* OR provide) NEAR/3 (results OR information OR risk)) OR ab((communicat* OR provide) NEAR/3 (results OR information OR risk)) OR ti("cue to action" OR "cues to action") OR ab("cue to action" OR "cues to action") OR ti((tailored OR personalised or personalized OR individualised or individualized) NEAR/5 (communicat* OR information)) OR ab((tailored OR personalised or personalized OR individualised or individualized) NEAR/5 (communicat* OR information)) OR ti(receiv* NEAR/3 (results OR information)) OR ab(receiv* NEAR/3 (results OR information)) OR ti((knowledge OR knowing) NEAR/3 result*) OR ab((knowledge OR knowing) NEAR/3 result*) OR ti(counsel*) OR ab(counsel*) OR ti(coach*) OR ab(coach*) OR ti(advice) OR ab(advice) OR ti("motivational interviewing") OR ab("motivational interviewing") OR ti((behavior OR behaviour) NEAR/1 (therap* OR intervention)) OR ab((behavior OR behaviour) NEAR/1 (therap* OR intervention)) OR ti((tailored OR personalised or personalized OR individualised or individualized) NEAR/3 educat*) OR ab((tailored OR personalised or personalized OR individualised or individualized) NEAR/3 educat*) OR ti(education NEAR/3 (module OR support OR session)) OR ab(education NEAR/3 (module OR support OR session)) OR ti(educate NEAR/3 (individual OR patient)) OR ab(educate NEAR/3 (individual OR patient)) OR ti(received NEAR/3 education*) OR ab(received NEAR/3 education*) OR ti((patient OR health) NEAR/1 education) OR ab((patient OR health) NEAR/1 education) OR ti(wearable) OR ab(wearable) OR ti(biosensor) OR ab(biosensor) OR ti(("sensor technologies" OR "sensor technology")) OR ab(("sensor technologies" OR "sensor technology")) OR ti(device NEAR/3 (monitor* OR test*)) OR ab(device NEAR/3 (monitor* OR test*)) OR ti(("smart device" OR "smart devices")) OR ab(("smart device" OR "smart devices")) OR ti(self NEAR/3 (monitor* OR test*)) OR ab(self NEAR/3 (monitor* OR test*)) OR ti("ambulatory monitor*") OR ab("ambulatory monitor*") OR ti("personal* health" NEAR/3 monitor*) OR ab("personal* health" NEAR/3 monitor*) OR ti(messag*) OR ab(messag*) OR ti(telephone OR cellphone OR cell-phone OR smartphone OR smart-phone OR ("mobile phone" OR "mobile phones")) OR ab(telephone OR cellphone OR cell-phone OR smartphone OR smart-phone OR ("mobile phone" OR "mobile phones")) OR ti((month* OR week*) NEAR/3 support) OR ab((month* OR week*) NEAR/3 support) OR ti((interpreted OR reported OR informed) NEAR/3 (measurement OR result OR data OR level)) OR ab((interpreted OR reported OR informed) NEAR/3 (measurement OR result OR data OR level)))) OR (Exact("biofeedback" OR "glucose monitoring") OR ti((home OR self OR continuous OR ambulatory) NEAR/3 ((“blood pressure” or glucose) p/0 monitor*)) OR ab((home OR self OR continuous OR ambulatory) NEAR/3 ((“blood pressure” or glucose ) p/0 monitor*)) OR ti((“flash glucose” or hr or “heart rate”) p/0 monitor*”) OR ab((“flash glucose” or hr or “heart rate”) p/0 monitor*”) OR ti(“physiological feedback”) OR ab(“physiological feedback”) OR ti(“biological feedback”) OR ab(“biological feedback”) OR ti(biofeedback) OR ab(biofeedback) OR ti(“self weigh*”) OR ab(“self weigh*”) OR ti(“daily weigh*”) OR ab(“daily weigh*”) OR ti(connected NEAR/1 (glucometer OR scale)) OR ab(connected NEAR/1 (glucometer OR scale)) OR ti(“smart scale”) OR ab(“smart scale”) OR ti(BIA NEAR/3 scale) OR ab(BIA NEAR/3 scale) OR ti((“bioelectric impedance” OR “bioelectrical impedance” OR “bio-electric impedance” OR “bio-electrical impedance”) NEAR/3 (scale OR analysis)) OR ab((“bioelectric impedance” OR “bioelectrical impedance” OR “bio-electric impedance” OR “bio-electrical impedance”) NEAR/3 (scale OR analysis)) OR ti((bioimpedance OR bio-impedance) NEAR/3 (scale OR analysis)) OR ab((bioimpedance OR bio-impedance) NEAR/3 (scale OR analysis)))) AND (Exact("behavior" OR "health behavior" OR "behavior modification" OR "eating behavior" OR "patient compliance" OR "self awareness" OR "disease prevention" OR "compliance" OR "motivation" OR "preventive medicine" OR "health promotion" OR "public health" OR "public health health sciences" OR "prevention" OR "harm reduction") OR ti((behavior OR behaviour OR lifestyle) NEAR/3 (chang* OR modif* OR promot*)) OR ab ti((behavior OR behaviour OR lifestyle) NEAR/3 (chang* OR modif* OR promot*)) OR ti(self NEAR/3 (care OR management OR efficacy)) OR ab(self NEAR/3 (care OR management OR efficacy)) OR ti(health p/0 behavior OR health p/0 behaviour) OR ab(health p/0 behavior OR health p/0 behaviour) OR ti(“healthy lifestyle”) OR ab(“healthy lifestyle”) OR ti(awareness) OR ab(awareness) OR ti((risk OR harm OR “sedentary behavior” OR “sedentary behaviour”) NEAR/3 reduc*) OR ab((risk OR harm OR “sedentary behavior” OR “sedentary behaviour”) NEAR/3 reduc*) OR ti(“weight loss”) OR ab(“weight loss”) OR ti(“weight control”) OR ab(“weight control”) OR ti(smok* NEAR/3 (behavior OR behaviour OR cessation OR quit*)) OR ab(smok* NEAR/3 (behavior OR behaviour OR cessation OR quit*)) OR ti(("self regulate" OR "self regulated" OR "self regulating" OR "self regulation" OR "self regulatory")) OR ab(("self regulate" OR "self regulated" OR "self regulating" OR "self regulation" OR "self regulatory")) OR ti(motivated OR motivation) OR ab(motivated OR motivation) OR ti(adherence OR compliance) OR ab(adherence OR compliance) OR ti(prevention OR preventive) OR ab(prevention OR preventive) OR ti(improv* NEAR/3 (activit* OR eating OR diet* OR health OR fitness)) OR AB(improv* NEAR/3 (activit* OR eating OR diet* OR health OR fitness)) OR ti((exercise OR “physical activity” OR diet* OR eating OR weight) NEAR/3 (behavior OR behaviour OR chang* OR maint* OR motivat* OR promot* OR modif*)) OR ab((exercise OR “physical activity” OR diet* OR eating OR weight) NEAR/3 (behavior OR behaviour OR chang* OR maint* OR motivat* OR promot* OR modif*)) OR ti(“public health”) OR ab(“public health”))) AND (ti(random*) OR ab(random*) OR ti(trial) OR ab(trial))
