# Supplementary material for: Use of Biological Feedback as a Health Behavior Change Technique in Adults: Scoping Review
Source: J Med Internet Res. 2023 Sep 25;25:e44359. doi: 10.2196/44359 (PMC10562972; doi:10.2196/44359)
Supplement: Multimedia Appendix 3 [file jmir_v25i1e44359_app3.docx]

**Multimedia Appendix 3: Behavior change theories, models, and frameworks cited in biological feedback literature and the domains of research in which they were cited.** Multimedia appendix 3 displays all theories, models, and frameworks cited 2 or more times, and in which domain of research they were cited. While a majority of studies did not mention a behavior change theory, model, or framework (n = 462, 60.2%), the remaining 305 articles (39.8%) cited 102 total theoretical frameworks. Notably, in 105 of the 305 articles (34.4%), it was unclear if the theoretical framework(s) cited were used to guide the design of the biological feedback intervention [data not shown].

|  | Diabetes | CVD | Obesity | Substance Use | Health Promotion | Cancer | Osteoporosis | Pregnancy/ | Kidney Disease | Infectious Disease | Respiratory Disease | Mental Health | Metabolic Syndrome | Other | **Total** |
| --- | --- | --- | --- | --- | --- | --- | --- | --- | --- | --- | --- | --- | --- | --- | --- |
|  |  |  |  |  |  |  |  | Postpartum |  |  |  |  |  |  |  |
| Transtheoretical Model | 13 | 19 | 8 | 22 | 9 | 5 | 1 | 1 | 2 | 2 |  | 1 | 1 | 2 | 86 |
| Social Cognitive Theory | 12 | 9 | 22 | 6 | 4 | 1 | 1 | 5 |  |  | 1 |  |  | 1 | 62 |
| Self-Regulation Theory | 7 | 5 | 22 | 3 | 4 | 2 |  | 2 | 1 | 1 |  |  |  | 1 | 48 |
| Health Belief Model | 8 | 13 | 1 | 6 | 4 | 5 | 4 |  | 2 | 1 |  |  |  |  | 44 |
| Self-Efficacy Theory | 12 | 10 | 4 | 2 | 2 |  | 3 |  | 1 |  | 2 |  |  | 1 | 37 |
| Theory of Planned Behavior | 6 | 10 | 2 | 3 | 3 | 4 | 1 | 1 |  |  |  | 1 |  | 1 | 32 |
| Chronic Care Model | 12 | 11 |  |  |  |  |  |  | 1 |  |  |  |  |  | 24 |
| Social Learning Theory | 7 | 5 | 3 | 2 | 4 |  | 2 | 1 |  |  |  |  |  |  | 15 |
| Self-Determination Theory | 4 | 4 | 6 |  |  |  | 1 |  |  |  |  |  |  |  | 15 |
| Protection Motivation Theory | 3 | 2 |  | 4 | 2 | 1 | 1 |  |  |  |  |  |  | 1 | 14 |
| Common Sense Model | 6 | 3 |  |  | 1 |  |  |  | 1 | 1 |  |  |  |  | 12 |
| Control Theory | 1 | 1 | 6 |  |  |  |  |  |  |  |  |  |  |  | 8 |
| Precaution Adoption Model |  |  |  | 3 |  | 2 | 1 |  |  |  |  |  |  |  | 6 |
| IMB Model | 3 |  |  |  | 1 |  |  |  |  | 1 |  |  |  |  | 5 |
| Extended Parallel Process Model |  |  |  |  | 1 | 3 |  |  |  |  |  |  |  |  | 4 |
| Goal Setting Theory | 2 | 1 | 1 |  |  |  |  |  |  |  |  |  |  |  | 4 |
| Problem-Solving Theory | 2 |  | 2 |  |  |  |  |  |  |  |  |  |  |  | 4 |
| CALO-RE Taxonomy |  |  | 3 |  |  |  |  |  |  |  |  |  |  |  | 3 |
| Precede-Proceed Model | 1 | 2 |  |  |  |  |  |  |  |  |  |  |  |  | 3 |
| Prospect Theory | 2 |  | 1 |  |  |  |  |  |  |  |  |  |  |  | 3 |
| Theory of Cognitive Dissonance |  | 1 | 1 | 1 |  |  |  |  |  |  |  |  |  |  | 3 |
| 5A Model | 1 |  |  | 1 |  |  |  |  |  |  |  |  |  |  | 2 |
| Empowerment Philosophy | 2 |  |  |  |  |  |  |  |  |  |  |  |  |  | 2 |
| Expectancy Theory |  |  |  | 1 | 1 |  |  |  |  |  |  |  |  |  | 2 |
| Health Action Process Approach Model | 1 |  | 1 |  |  |  |  |  |  |  |  |  |  |  | 2 |
| Model of Action Phases |  | 2 |  |  |  |  |  |  |  |  |  |  |  |  | 2 |
| Normalization Process Theory |  | 1 |  |  |  |  |  | 1 |  |  |  |  |  |  | 2 |
| Patient Activation Theory |  | 1 |  |  |  |  | 1 |  |  |  |  |  |  |  | 2 |
| RE-AIM Framework | 2 |  |  |  |  |  |  |  |  |  |  |  |  |  | 2 |
| Reference Group Theory | 1 |  |  | 1 |  |  |  |  |  |  |  |  |  |  | 2 |
| Relapse Prevention Theory |  |  | 2 |  |  |  |  |  |  |  |  |  |  |  | 2 |
| Social Comparison Theory |  |  | 1 |  |  | 1 |  |  |  |  |  |  |  |  | 2 |
| Social Ecological Model | 1 |  | 1 |  |  |  |  |  |  |  |  |  |  |  | 2 |
| Social Support Theory |  | 2 |  |  |  |  |  |  |  |  |  |  |  |  | 2 |
| Supportive Accountability Model |  |  | 2 |  |  |  |  |  |  |  |  |  |  |  | 2 |
| Theory of Habit Formation |  |  | 1 |  | 1 |  |  |  |  |  |  |  |  |  | 2 |
| **TOTAL** | 109 | 102 | 90 | 55 | 37 | 24 | 16 | 11 | 8 | 6 | 3 | 2 | 1 | 7 | 471 |
