# Supplementary material for: Use of Biological Feedback as a Health Behavior Change Technique in Adults: Scoping Review
Source: J Med Internet Res. 2023 Sep 25;25:e44359. doi: 10.2196/44359 (PMC10562972; doi:10.2196/44359)
Supplement: Multimedia Appendix 4 [file jmir_v25i1e44359_app4.docx]

**References of Articles Included in the Review:**

1. Adachi Y, Sato C, Yamatsu K, Ito S, Adachi K, Yamagami T. A randomized controlled trial on the long-term effects of a 1-month behavioral weight control program assisted by computer tailored advice. Behav Res Ther. 2007 Mar;45(3):459-70. PMID: 16713991.

2. Addley K, Boyd S, Kerr R, McQuillan P, Houdmont J, McCrory M. The impact of two workplace-based health risk appraisal interventions on employee lifestyle parameters, mental health and work ability: results of a randomized controlled trial. Health Education Research. 2014 Apr;29(2):247-58. PMID: 24399261. doi: https://dx.doi.org/10.1093/her/cyt113.

3. Aekplakorn W, Suriyawongpaisal P, Tansirisithikul R, Sakulpipat T, Charoensuk P. Effectiveness of Self-Monitoring Blood Pressure in Primary Care: A Randomized Controlled Trial. J. 2016 Apr;7(2):58-64. PMID: 26574566. doi: https://dx.doi.org/10.1177/2150131915614069.

4. Agarwal P, Mukerji G, Desveaux L, Ivers NM, Bhattacharyya O, Hensel JM, et al. Mobile App for Improved Self-Management of Type 2 Diabetes: Multicenter Pragmatic Randomized Controlled Trial. JMIR Mhealth Uhealth. 2019 01 10;7(1):e10321. PMID: 30632972. doi: https://dx.doi.org/10.2196/10321.

5. Ahn WK, Lebowitz MS. An experiment assessing effects of personalized feedback about genetic susceptibility to obesity on attitudes towards diet and exercise. Appetite. 2018 Jan 01;120:23-31. PMID: 28837820. doi: https://dx.doi.org/10.1016/j.appet.2017.08.021.

6. Ahring KK, Ahring JP, Joyce C, Farid NR. Telephone modem access improves diabetes control in those with insulin-requiring diabetes. Diabetes Care. 1992 Aug;15(8):971-5. PMID: 1505329.

7. Aiello LP, Ayala AR, Antoszyk AN, Arnold-Bush B, Baker C, Bressler NM, et al. Assessing the Effect of Personalized Diabetes Risk Assessments During Ophthalmologic Visits on Glycemic Control: A Randomized Clinical Trial. JAMA Ophthalmol. 2015 Aug;133(8):888-96. PMID: 25996273. doi: https://dx.doi.org/10.1001/jamaophthalmol.2015.1312.

8. Ajjan RA, Jackson N, Thomson SA. Reduction in HbA1c using professional flash glucose monitoring in insulin-treated type 2 diabetes patients managed in primary and secondary care settings: A pilot, multicentre, randomised controlled trial. Diab Vasc Dis Res. 2019 07;16(4):385-95. PMID: 31271312. doi: https://dx.doi.org/10.1177/1479164119827456.

9. Al-Ofi EA, Mosli HH, Ghamri KA, Ghazali SM. Management of postprandial hyperglycaemia and weight gain in women with gestational diabetes mellitus using a novel telemonitoring system. J Int Med Res. 2019 Feb;47(2):754-64. PMID: 30442052. doi: https://dx.doi.org/10.1177/0300060518809872.

10. Alanzi T, Alanazi NR, Istepanian R, Philip N. Evaluation of the effectiveness of mobile diabetes management system with social networking and cognitive behavioural therapy (CBT) for T2D. Mhealth. 2018;4:35. PMID: 30221168. doi: https://dx.doi.org/10.21037/mhealth.2018.06.05.

11. Alemi F, Higley P. Reaction to "talking" computers assessing health risks. Med Care. 1995 Mar;33(3):227-33. PMID: 7861825.

12. Alencar M, Johnson K, Gray V, Mullur R, Gutierrez E, Dionico P. Telehealth-Based Health Coaching Increases m-Health Device Adherence and Rate of Weight Loss in Obese Participants. Telemed J E Health. 2020 03;26(3):365-8. PMID: 30994410. doi: https://dx.doi.org/10.1089/tmj.2019.0017.

13. Alencar MK, Johnson K, Mullur R, Gray V, Gutierrez E, Korosteleva O. The efficacy of a telemedicine-based weight loss program with video conference health coaching support. J Telemed Telecare. 2019 Apr;25(3):151-7. PMID: 29199544. doi: https://dx.doi.org/10.1177/1357633X17745471.

14. Alessi SM, Petry NM. Smoking reductions and increased self-efficacy in a randomized controlled trial of smoking abstinence-contingent incentives in residential substance abuse treatment patients. Nicotine Tob Res. 2014 Nov;16(11):1436-45. PMID: 24935755. doi: https://dx.doi.org/10.1093/ntr/ntu095.

15. Alfadhli E, Osman E, Basri T. Use of a real time continuous glucose monitoring system as an educational tool for patients with gestational diabetes. Diabetol Metab Syndr. 2016;8:48. PMID: 27468313. doi: https://dx.doi.org/10.1186/s13098-016-0161-5.

16. Alick CL, Samuel-Hodge C, Ward D, Ammerman A, Rini C, Tate DF. Together Eating & Activity Matters (TEAM): results of a pilot randomized-clinical trial of a spousal support weight loss intervention for Black men. Obes. 2018 02;4(1):62-75. PMID: 29479466. doi: https://dx.doi.org/10.1002/osp4.142.

17. Allen JK, Stephens J, Dennison Himmelfarb CR, Stewart KJ, Hauck S. Randomized controlled pilot study testing use of smartphone technology for obesity treatment. J Obes. 2013;2013:151597. PMID: 24392223. doi: https://dx.doi.org/10.1155/2013/151597.

18. Allen N, Whittemore R, Melkus G. A continuous glucose monitoring and problem-solving intervention to change physical activity behavior in women with type 2 diabetes: a pilot study. Diabetes Technol Ther. 2011 Nov;13(11):1091-9. PMID: 21919735. doi: https://dx.doi.org/10.1089/dia.2011.0088.

19. Allen NA, Fain JA, Braun B, Chipkin SR. Continuous glucose monitoring counseling improves physical activity behaviors of individuals with type 2 diabetes: A randomized clinical trial. Diabetes Res Clin Pract. 2008 Jun;80(3):371-9. PMID: 18304674. doi: https://dx.doi.org/10.1016/j.diabres.2008.01.006.

20. Allen JK, Blumenthal RS, Margolis S, Young DR, Miller ER, rd K. Nurse case management of hypercholesterolemia in patients with coronary heart disease: results of a randomized clinical trial. Am Heart J. 2002 Oct;144(4):678-86. PMID: 12360165.

21. Allen BT, DeLong ER, Feussner JR. Impact of glucose self-monitoring on non-insulin-treated patients with type II diabetes mellitus. Randomized controlled trial comparing blood and urine testing. Diabetes Care. 1990 Oct;13(10):1044-50. PMID: 2170088.

22. Almeida VA, Littlejohn P, Cop I, Brown E, Afroze R, Davison KM. Comparison of nutrigenomics technology interface tools for consumers and health professionals: A sequential explanatory mixed methods investigation. Journal of Medical Internet Research. 2019;21(6). PMID: 2019-43494-001.

23. Alotaibi MM, Istepanian R, Philip N. A mobile diabetes management and educational system for type-2 diabetics in Saudi Arabia (SAED). Mhealth. 2016;2:33. PMID: 28293606. doi: https://dx.doi.org/10.21037/mhealth.2016.08.01.

24. Amante DJ, Harlan DM, Lemon SC, McManus DD, Olaitan OO, Pagoto SL, et al. Evaluation of a Diabetes Remote Monitoring Program Facilitated by Connected Glucose Meters for Patients With Poorly Controlled Type 2 Diabetes: Randomized Crossover Trial. JMIR Diabetes. 2021 Mar 11;6(1):e25574. PMID: 33704077. doi: https://dx.doi.org/10.2196/25574.

25. Anderson AS, Craigie AM, Caswell S, Treweek S, Stead M, Macleod M, et al. The impact of a bodyweight and physical activity intervention (BeWEL) initiated through a national colorectal cancer screening programme: randomised controlled trial. Bmj. 2014 Mar 07;348:g1823. PMID: 24609919. doi: https://dx.doi.org/10.1136/bmj.g1823.

26. Anderson RM, Funnell MM, Aikens JE, Krein SL, Fitzgerald JT, Nwankwo R, et al. Evaluating the Efficacy of an Empowerment-Based Self-Management Consultant Intervention: Results of a Two-Year Randomized Controlled Trial. Ther. 2009 Jun 01;1(1):3-11. PMID: 20076768.

27. Anderson RC. Worksite health promotion: The benefits of providing personal health status feedback to employees [Ph.D.]. Ann Arbor: Marquette University; 1988.

28. Anzaldo-Campos MC, Contreras S, Vargas-Ojeda A, Menchaca-Diaz R, Fortmann A, Philis-Tsimikas A. Dulce Wireless Tijuana: A Randomized Control Trial Evaluating the Impact of Project Dulce and Short-Term Mobile Technology on Glycemic Control in a Family Medicine Clinic in Northern Mexico. Diabetes Technol Ther. 2016 Apr;18(4):240-51. PMID: 26914371. doi: https://dx.doi.org/10.1089/dia.2015.0283.

29. Araujo Almeida V, Littlejohn P, Cop I, Brown E, Afroze R, Davison KM. Comparison of Nutrigenomics Technology Interface Tools for Consumers and Health Professionals: A Sequential Explanatory Mixed Methods Investigation. Journal of Medical Internet Research. 2019 06 28;21(6):e12580. PMID: 31254340. doi: https://dx.doi.org/10.2196/12580.

30. Arthur C, Di Corleto E, Ballard E, Kothari A. A randomized controlled trial of daily weighing in pregnancy to control gestational weight gain. BMC Pregnancy Childbirth. 2020 Apr 16;20(1):223. PMID: 32299371. doi: https://dx.doi.org/10.1186/s12884-020-02884-1.

31. Artinian NT, Flack JM, Nordstrom CK, Hockman EM, Washington OG, Jen KL, et al. Effects of nurse-managed telemonitoring on blood pressure at 12-month follow-up among urban African Americans. Nurs Res. 2007 Sep-Oct;56(5):312-22. PMID: 17846552.

32. Artinian NT, Harden JK, Kronenberg MW, Vander Wal JS, Daher E, Stephens Q, et al. Pilot study of a Web-based compliance monitoring device for patients with congestive heart failure. Heart Lung. 2003 Jul-Aug;32(4):226-33. PMID: 12891162.

33. Aston ER, Neiberg RH, Liguori A. Breath alcohol estimation training: behavioral effects and predictors of success. Alcohol Alcohol. 2013 Jul-Aug;48(4):396-401. PMID: 23695976. doi: https://dx.doi.org/10.1093/alcalc/agt047.

34. Athar MW, Record JD, Martire C, Hellmann DB, Ziegelstein RC. The Effect of a Personalized Approach to Patient Education on Heart Failure Self-Management. J. 2018 Nov 27;8(4):27. PMID: 30486472. doi: https://dx.doi.org/10.3390/jpm8040039.

35. Aubin M, Godin G, Vezina L, Maziade J, Desharnais R. Hypercholesterolemia screening. Does knowledge of blood cholesterol level affect dietary fat intake? Can Fam Physician. 1998 Jun;44:1289-97. PMID: 9640523.

36. Audrain J, Boyd NR, Roth J, Main D, Caporaso NE, Lerman C. Genetic susceptibility testing in smoking-cessation treatment: One-year outcomes of a randomized trial. Addictive Behaviors. 1997;22(6):741-51. PMID: 1998-10852-002. doi: 10.1016/S0306-4603(97)00060-9.

37. Aung MN, Yuasa M, Moolphate S, Lorga T, Yokokawa H, Fukuda H, et al. Effectiveness of a new multi-component smoking cessation service package for patients with hypertension and diabetes in northern Thailand: a randomized controlled trial (ESCAPE study). Subst Abuse Treat Prev Policy. 2019 02 22;14(1):10. PMID: 30795811. doi: https://dx.doi.org/10.1186/s13011-019-0197-2.

38. Austin DL. SELECTED NURSING INTERVENTIONS FOR NONCOMPLIANT HYPERTENSIVE PATIENTS [Ph.D.]. Ann Arbor: Texas Woman's University; 1986.

39. Averill F, Brown TG, Robertson RD, Tchomgang A, Berbiche D, Nadeau L, et al. Transdermal alcohol monitoring combined with contingency management for driving while impaired offenders: A pilot randomized controlled study. Traffic inj prev. 2018 07 04;19(5):455-61. PMID: 29543499. doi: https://dx.doi.org/10.1080/15389588.2018.1448079.

40. Ayres K, Conner M, Prestwich A, Hurling R, Cobain M, Lawton R, et al. Exploring the question‐behaviour effect: Randomized controlled trial of motivational and question‐behaviour interventions. British Journal of Health Psychology. 2013;18(1):31-44. PMID: 2013-01559-004. doi: 10.1111/j.2044-8287.2012.02075.x.

41. Ayres K, Conner M, Prestwich A, Hurling R, Cobain M, Lawton R, et al. Exploring the question-behaviour effect: randomized controlled trial of motivational and question-behaviour interventions. British Journal of Health Psychology. 2013 Feb;18(1):31-44. PMID: 22519696. doi: https://dx.doi.org/10.1111/j.2044-8287.2012.02075.x.

42. Babazono A, Kame C, Ishihara R, Yamamoto E, Hillman AL. Patient-motivated prevention of lifestyle-related disease in Japan: a randomized, controlled clinical trial. Disease management and health outcomes. 2007;15(2):119‐26. PMID: CN-00641540. doi: 10.2165/00115677-200715020-00007.

43. Baghianimoghadam MH, Rahaee Z, Morowatisharifabad MA, Sharifirad G, Andishmand A, Azadbakht L. Effects of education on self-monitoring of blood pressure based on BASNEF model in hypertensive patients. J. 2010 Mar;15(2):70-7. PMID: 21526062.

44. Bai Y, Wu X, Tsang RC, Yun R, Lu Y, Dean E, et al. A Randomised Controlled Trial to Evaluate the Administration of the Health Improvement Card as a Health Promotion Tool: A Physiotherapist-Led Community-Based Initiative. Int J Environ Res Public Health. 2020 11 02;17(21):02. PMID: 33147721. doi: https://dx.doi.org/10.3390/ijerph17218065.

45. Balzola F, Cullen G, Ho GT, Russell RK, Wehkamp J. Effect of communicating DNA based risk assessments for Crohn's disease on smoking cessation: Randomised controlled trial. Inflammatory Bowel Disease Monitor. 2012;13(2):74-5.

46. Barcelo A, Robles S, White F, Jadue L, Vega J. [An intervention to improve diabetes control in Chile]. Rev Panam Salud Publica. 2001 Nov;10(5):328-33. PMID: 11774805.

47. Barnabas RV, van Rooyen H, Tumwesigye E, Brantley J, Baeten JM, van Heerden A, et al. Uptake of antiretroviral therapy and male circumcision after community-based HIV testing and strategies for linkage to care versus standard clinic referral: a multisite, open-label, randomised controlled trial in South Africa and Uganda. Lancet HIV. 2016 05;3(5):e212-20. PMID: 27126488. doi: https://dx.doi.org/10.1016/S2352-3018(16)00020-5.

48. Barnfather KD, Cope GF, Chapple IL. Effect of incorporating a 10 minute point of care test for salivary nicotine metabolites into a general practice based smoking cessation programme: randomised controlled trial. Bmj. 2005 Oct 29;331(7523):999. PMID: 16210250.

49. Baron JS, Hirani S, Newman SP. A randomised, controlled trial of the effects of a mobile telehealth intervention on clinical and patient-reported outcomes in people with poorly controlled diabetes. J Telemed Telecare. 2017 Feb;23(2):207-16. PMID: 26880694. doi: https://dx.doi.org/10.1177/1357633X16631628.

50. Barrio P, Teixidor L, Ortega L, Lligoña A, Rico N, Bedini JL, et al. Filling the gap between lab and clinical impact: An open randomized diagnostic trial comparing urinary ethylglucuronide and ethanol in alcohol dependent outpatients. Drug and Alcohol Dependence. 2018;183:225-30. PMID: 2018-05035-035. doi: 10.1016/j.drugalcdep.2017.11.015.

51. Batalik L, Dosbaba F, Hartman M, Batalikova K, Spinar J. Benefits and effectiveness of using a wrist heart rate monitor as a telerehabilitation device in cardiac patients: A randomized controlled trial. Medicine (Baltimore). 2020 Mar;99(11):e19556. PMID: 32176113. doi: https://dx.doi.org/10.1097/MD.0000000000019556.

52. Baumann LJ, Keller ML. Responses to threat information. Image J Nurs Sch. 1991;23(1):13-8. PMID: 2022383.

53. Bender MS, Cooper BA, Park LG, Padash S, Arai S. A Feasible and Efficacious Mobile-Phone Based Lifestyle Intervention for Filipino Americans with Type 2 Diabetes: Randomized Controlled Trial. JMIR Diabetes. 2017 Dec 12;2(2):e30. PMID: 30291068. doi: https://dx.doi.org/10.2196/diabetes.8156.

54. Benhamou PY, Melki V, Boizel R, Perreal F, Quesada JL, Bessieres-Lacombe S, et al. One-year efficacy and safety of Web-based follow-up using cellular phone in type 1 diabetic patients under insulin pump therapy: the PumpNet study. Diabetes Metab. 2007 Jun;33(3):220-6. PMID: 17395516.

55. Benner JS, Erhardt L, Flammer M, Moller RA, Rajicic N, Changela K, et al. A novel programme to evaluate and communicate 10-year risk of CHD reduces predicted risk and improves patients' modifiable risk factor profile. Int J Clin Pract. 2008 Oct;62(10):1484-98. PMID: 18691228. doi: https://dx.doi.org/10.1111/j.1742-1241.2008.01872.x.

56. Bennett GG, Steinberg D, Askew S, Levine E, Foley P, Batch BC, et al. Effectiveness of an App and Provider Counseling for Obesity Treatment in Primary Care. American Journal of Preventive Medicine. 2018 12;55(6):777-86. PMID: 30361140. doi: https://dx.doi.org/10.1016/j.amepre.2018.07.005.

57. Bergenstal RM, Bode BW, Tamler R, Trence DL, Stenger P, Schachner HC, et al. Advanced meter features improve postprandial and paired self-monitoring of blood glucose in individuals with diabetes: results of the Actions with the CONTOUR Blood Glucose Meter and Behaviors in Frequent Testers (ACT) study. Diabetes Technol Ther. 2012 Oct;14(10):851-7. PMID: 23013200.

58. Bertz F, Pacanowski CR, Levitsky DA. Frequent self‐weighing with electronic graphic feedback to prevent age‐related weight gain in young adults. Obesity. 2015;23(10):2009-14. PMID: 2015-45184-013. doi: 10.1002/oby.21211.

59. Beyth RJ, Quinn L, Landefeld CS. A multicomponent intervention to prevent major bleeding complications in older patients receiving warfarin: A randomized, controlled trial. Annals of Internal Medicine. 2000;133(9):687-95. PMID: 11074901. doi: 10.7326/0003-4819-133-9-200011070-00010.

60. Binstock ML, Franklin KL. A comparison of compliance techniques on the control of high blood pressure. Am J Hypertens. 1988 Jul;1(3 Pt 3):192S-4S. PMID: 3415798.

61. Bittman B, Poornima I, Smith MA, Heidel RE. Gospel Music: A Catalyst for Retention, Engagement, and Positive Health Outcomes for African Americans in a Cardiovascular Prevention and Treatment Program. Adv Mind Body Med. 2020 Winter;34(1):8-16. PMID: 32277749.

62. Blasco A, Carmona M, Fernandez-Lozano I, Salvador CH, Pascual M, Sagredo PG, et al. Evaluation of a telemedicine service for the secondary prevention of coronary artery disease. J Mol Signal. 2012 Jan-Feb;32(1):25-31. PMID: 22113368. doi: https://dx.doi.org/10.1097/HCR.0b013e3182343aa7.

63. Block G, Azar KM, Romanelli RJ, Block TJ, Hopkins D, Carpenter HA, et al. Diabetes Prevention and Weight Loss with a Fully Automated Behavioral Intervention by Email, Web, and Mobile Phone: A Randomized Controlled Trial Among Persons with Prediabetes. Journal of Medical Internet Research. 2015 Oct 23;17(10):e240. PMID: 26499966. doi: https://dx.doi.org/10.2196/jmir.4897.

64. Bloss CS, Wineinger NE, Peters M, Boeldt DL, Ariniello L, Kim JY, et al. A prospective randomized trial examining health care utilization in individuals using multiple smartphone-enabled biosensors. Peerj. 2016;4:e1554. PMID: 26788432. doi: https://dx.doi.org/10.7717/peerj.1554.

65. Boaz M, Hellman K, Wainstein J. An automated telemedicine system improves patient-reported well-being. Diabetes Technol Ther. 2009 Mar;11(3):181-6. PMID: 19216685. doi: https://dx.doi.org/10.1089/dia.2008.0048.

66. Bollyky JB, Bravata D, Yang J, Williamson M, Schneider J. Remote Lifestyle Coaching Plus a Connected Glucose Meter with Certified Diabetes Educator Support Improves Glucose and Weight Loss for People with Type 2 Diabetes. J Diabetes Res. 2018;2018:3961730. PMID: 29888288. doi: https://dx.doi.org/10.1155/2018/3961730.

67. BonAd ASG, Kung AW, Rachman IA, Adam JM, Roeshadi D, Torralba T, et al. Impact of bone marker feedback on adherence to once monthly ibandronate for osteoporosis among Asian postmenopausal women. Int J Rheum Dis. 2009 Sep;12(3):216-24. PMID: 20374349. doi: https://dx.doi.org/10.1111/j.1756-185X.2009.01413.x.

68. Bonner C, Jansen J, Newell BR, Irwig L, Teixeira-Pinto A, Glasziou P, et al. Is the "heart Age" Concept Helpful or Harmful Compared to Absolute Cardiovascular Disease Risk? An Experimental Study. Medical Decision Making. 2015 Nov;35(8):967-78. PMID: 26251465. doi: 10.1177/0272989X15597224.

69. Bonomo K, De Salve A, Fiora E, Mularoni E, Massucco P, Poy P, et al. Evaluation of a simple policy for pre- and post-prandial blood glucose self-monitoring in people with type 2 diabetes not on insulin. Diabetes Res Clin Pract. 2010 Feb;87(2):246-51. PMID: 19954855. doi: https://dx.doi.org/10.1016/j.diabres.2009.10.021.

70. Borrelli B, McQuaid EL, Novak SP, Hammond SK, Becker B. Motivating Latino caregivers of children with asthma to quit smoking: a randomized trial. J Consult Clin Psychol. 2010 Feb;78(1):34-43. PMID: 20099948. doi: https://dx.doi.org/10.1037/a0016932.

71. Borrelli B, Novak S, Hecht J, Emmons K, Papandonatos G, Abrams D. Home health care nurses as a new channel for smoking cessation treatment: outcomes from project CARES (Community-nurse Assisted Research and Education on Smoking). Prev Med. 2005 Nov-Dec;41(5-6):815-21. PMID: 16182355.

72. Bosworth HB, Powers BJ, Olsen MK, McCant F, Grubber J, Smith V, et al. Home blood pressure management and improved blood pressure control: results from a randomized controlled trial. Arch Intern Med. 2011 Jul 11;171(13):1173-80. PMID: 21747013. doi: https://dx.doi.org/10.1001/archinternmed.2011.276.

73. Bosworth HB, Olsen MK, Grubber JM, Neary AM, Orr MM, Powers BJ, et al. Two self-management interventions to improve hypertension control: a randomized trial. Annals of Internal Medicine. 2009 Nov 17;151(10):687-95. PMID: 19920269. doi: https://dx.doi.org/10.7326/0003-4819-151-10-200911170-00148.

74. Boulware LE, Ephraim PL, Hill-Briggs F, Roter DL, Bone LR, Wolff JL, et al. Hypertension Self-management in Socially Disadvantaged African Americans: the Achieving Blood Pressure Control Together (ACT) Randomized Comparative Effectiveness Trial. Journal of General Internal Medicine. 2020 01;35(1):142-52. PMID: 31705466. doi: https://dx.doi.org/10.1007/s11606-019-05396-7.

75. Bove AA, Homko CJ, Santamore WP, Kashem M, Kerper M, Elliott DJ. Managing hypertension in urban underserved subjects using telemedicine--a clinical trial. Am Heart J. 2013 Apr;165(4):615-21. PMID: 23537980. doi: https://dx.doi.org/10.1016/j.ahj.2013.01.004.

76. Bove AA, Santamore WP, Homko C, Kashem A, Cross R, McConnell TR, et al. Reducing cardiovascular disease risk in medically underserved urban and rural communities. Am Heart J. 2011 Feb;161(2):351-9. PMID: 21315219. doi: https://dx.doi.org/10.1016/j.ahj.2010.11.008.

77. Bovet P, Perret F, Cornuz J, Quilindo J, Paccaud F. Improved smoking cessation in smokers given ultrasound photographs of their own atherosclerotic plaques. Prev Med. 2002 Feb;34(2):215-20. PMID: 11817917.

78. Brekke HK, Bertz F, Rasmussen KM, Bosaeus I, Ellegard L, Winkvist A. Diet and exercise interventions among overweight and obese lactating women: randomized trial of effects on cardiovascular risk factors. PLoS ONE. 2014;9(2):e88250. PMID: 24516621. doi: https://dx.doi.org/10.1371/journal.pone.0088250.

79. Brems C, Dewane SL, Johnson ME, Eldridge GD. Brief motivational interventions for HIV/STI risk reduction among individuals receiving alcohol detoxification. AIDS Educ Prev. 2009 Oct;21(5):397-414. PMID: 19842825. doi: https://dx.doi.org/10.1521/aeap.2009.21.5.397.

80. Brennan T, Spettell C, Villagra V, Ofili E, McMahill-Walraven C, Lowy EJ, et al. Disease management to promote blood pressure control among African Americans. Popul Health Manag. 2010 Apr;13(2):65-72. PMID: 20415618. doi: https://dx.doi.org/10.1089/pop.2009.0019.

81. Brindal E, Hendrie GA, Freyne J, Noakes M. A Mobile Phone App Designed to Support Weight Loss Maintenance and Well-Being (MotiMate): Randomized Controlled Trial. JMIR Mhealth Uhealth. 2019 09 04;7(9):e12882. PMID: 31486407. doi: https://dx.doi.org/10.2196/12882.

82. Brunette MF, Ferron JC, Drake RE, Devitt TS, Geiger PT, McHugo GJ, et al. Carbon monoxide feedback in a motivational decision support system for nicotine dependence among smokers with severe mental illnesses. Journal of Substance Abuse Treatment. 2013 Oct;45(4):319-24. PMID: 23706623. doi: https://dx.doi.org/10.1016/j.jsat.2013.04.005.

83. Buffels J, Degryse J, Decramer M, Heyrman J. Spirometry and smoking cessation advice in general practice: a randomised clinical trial. Respir Med. 2006 Nov;100(11):2012-7. PMID: 16580189.

84. Bujnowska-Fedak MM, Puchala E, Steciwko A. The impact of telehome care on health status and quality of life among patients with diabetes in a primary care setting in Poland. Telemed J E Health. 2011 Apr;17(3):153-63. PMID: 21375410. doi: https://dx.doi.org/10.1089/tmj.2010.0113.

85. Burkett PA, Southard DR, Herbert WG, Walberg J. Frequent cholesterol feedback as an aid in lowering cholesterol levels. Journal of Cardiopulmonary Rehabilitation. 1990;10(4):141-6. doi: 10.1097/00008483-199004000-00006.

86. Burling TA, Bigelow GE, Robinson JC, Mead AM. Smoking during pregnancy: Reduction via objective assessment and directive advice. Behavior Therapy. 1991;22(1):31-40.

87. Butryn ML, Martinelli MK, Crane NT, Godfrey K, Roberts SR, Zhang F, et al. Counselor Surveillance of Digital Self-Monitoring Data: A Pilot Randomized Controlled Trial. Obesity. 2020 12;28(12):2339-46. PMID: 33098278. doi: https://dx.doi.org/10.1002/oby.23015.

88. Butryn ML. A randomized trial of weight gain prevention interventions for young women: Effectiveness and influence on bulimic pathology: ProQuest Information & Learning; 2006.

89. Cabanas EAT. The impact of ethnicity and diet counseling on the outcomes of diabetes education [Ph.D.]. Ann Arbor: Texas Woman's University; 2001.

90. Cameron R, MacDonald MA, Schlegel RP, Young CI, Fisher SE, Killen JD, et al. Toward the development of self-help health behaviour change programs: weight loss by correspondence. Can J Public Health. 1990 Jul-Aug;81(4):275-9. PMID: 2207950.

91. Carnahan JE, Nugent CA. The effects of self-monitoring by patients on the control of hypertension. Am J Med Sci. 1975 Jan-Feb;269(1):69-73. PMID: 1130437.

92. Carrasco MP, Salvador CH, Sagredo PG, Marquez-Montes J, Gonzalez de Mingo MA, Fragua JA, et al. Impact of patient-general practitioner short-messages-based interaction on the control of hypertension in a follow-up service for low-to-medium risk hypertensive patients: a randomized controlled trial. IEEE Trans Inf Technol Biomed. 2008 Nov;12(6):780-91. PMID: 19000959. doi: https://dx.doi.org/10.1109/TITB.2008.926429.

93. Cavusoglu Y, Zoghi M, Eren M, Bozcali E, Kozdag G, Senturk T, et al. Post-discharge heart failure monitoring program in Turkey: Hit-PoinT. Anatol J Cardiol. 2017 Feb;17(2):107-12. PMID: 27488754. doi: https://dx.doi.org/10.14744/AnatolJCardiol.2016.6812.

94. Celis-Morales C, Livingstone KM, Marsaux CF, Macready AL, Fallaize R, O'Donovan CB, et al. Effect of personalized nutrition on health-related behaviour change: evidence from the Food4Me European randomized controlled trial. Int J Epidemiol. 2017 04 01;46(2):578-88. PMID: 27524815. doi: https://dx.doi.org/10.1093/ije/dyw186.

95. Celum CL, Gill K, Morton JF, Stein G, Myers L, Thomas KK, et al. Incentives conditioned on tenofovir levels to support PrEP adherence among young South African women: a randomized trial. J Int AIDS Soc. 2020 11;23(11):e25636. PMID: 33247553. doi: https://dx.doi.org/10.1002/jia2.25636.

96. Cerón JD, López DM, Urbano L, Álvarez-Rosero RE, Muñoz-Benítez S. Information and communication technology-based strategies for the reduction of cardiovascular risk factors in the active working population. Revista Colombiana de Cardiologia. 2018;25(1):92-100. doi: 10.1016/j.rccar.2017.08.018.

97. Cesare WF. EFFECTS OF EXERCISE ON DIETARY INDUCED WEIGHT LOSS [Educat.D.]. Ann Arbor: Boston University; 1985.

98. Chandler J, Sox L, Kellam K, Feder L, Nemeth L, Treiber F. Impact of a Culturally Tailored mHealth Medication Regimen Self-Management Program upon Blood Pressure among Hypertensive Hispanic Adults. Int J Environ Res Public Health. 2019 04 06;16(7):06. PMID: 30959858. doi: https://dx.doi.org/10.3390/ijerph16071226.

99. Chandraratne N, Yamaguchi M, Indrawansa S, Gunawardena N, Kuwahara K, Islam Z, et al. The effect of youths as change agents on cardiovascular disease risk factors among adult neighbours: a cluster randomised controlled trial in Sri Lanka. BMC Public Health. 2019 Jul 08;19(1):893. PMID: 31286931. doi: https://dx.doi.org/10.1186/s12889-019-7142-1.

100. Chao AM, Srinivas SK, Studt SK, Diewald LK, Sarwer DB, Allison KC. A Pilot Randomized Controlled Trial of a Technology-Based Approach for Preventing Excess Weight Gain during Pregnancy among Women with Overweight. Front. 2017;4:57. PMID: 29214155. doi: https://dx.doi.org/10.3389/fnut.2017.00057.

101. Chao J, Yang L, Xu H, Yu Q, Jiang L, Zong M. The effect of integrated health management model on the health of older adults with diabetes in a randomized controlled trial. Arch Gerontol Geriatr. 2015 Jan-Feb;60(1):82-8. PMID: 25456892. doi: https://dx.doi.org/10.1016/j.archger.2014.10.006.

102. Chao S, Roberts JS, Marteau TM, Silliman R, Cupples LA, Green RC. Health behavior changes after genetic risk assessment for Alzheimer disease: The REVEAL study. Alzheimer Disease and Associated Disorders. 2008 Jan-Mar;22(1):94-7. PMID: 18317253. doi: 10.1097/WAD.0b013e31815a9dcc.

103. Chapin RB, Williams DC, Adair RF. Diabetes control improved when inner-city patients received graphic feedback about glycosylated hemoglobin levels. Journal of General Internal Medicine. 2003 Feb;18(2):120-4. PMID: 12542586.

104. Charlson ME, Peterson JC, Boutin-Foster C, Briggs WM, Ogedegbe GG, McCulloch CE, et al. Changing health behaviors to improve health outcomes after angioplasty: a randomized trial of net present value versus future value risk communication. Health Education Research. 2008 Oct;23(5):826-39. PMID: 18025064.

105. Chau JP, Lee DT, Yu DS, Chow AY, Yu WC, Chair SY, et al. A feasibility study to investigate the acceptability and potential effectiveness of a telecare service for older people with chronic obstructive pulmonary disease. Int J Med Inf. 2012 Oct;81(10):674-82. PMID: 22789911. doi: https://dx.doi.org/10.1016/j.ijmedinf.2012.06.003.

106. Chesney MA, Black GW, Swan GE, Ward MM. Relaxation training for essential hypertension at the worksite: I. The untreated mild hypertensive. Psychosom Med. 1987 May-Jun;49(3):250-63. PMID: 3299442.

107. Chmiel C, Senn O, Rosemann T, Del Prete V, Steurer-Stey C. CoCo trial: Color-coded blood pressure Control, a randomized controlled study. Patient Prefer Adherence. 2014;8:1383-92. PMID: 25346595. doi: https://dx.doi.org/10.2147/PPA.S68213.

108. Cho JH, Kwon HS, Kim HS, Oh JA, Yoon KH. Effects on diabetes management of a health-care provider mediated, remote coaching system via a PDA-type glucometer and the Internet. J Telemed Telecare. 2011;17(7):365-70. PMID: 21933896. doi: https://dx.doi.org/10.1258/jtt.2011.100913.

109. Cho JH, Lee HC, Lim DJ, Kwon HS, Yoon KH. Mobile communication using a mobile phone with a glucometer for glucose control in Type 2 patients with diabetes: as effective as an Internet-based glucose monitoring system. J Telemed Telecare. 2009;15(2):77-82. PMID: 19246607. doi: https://dx.doi.org/10.1258/jtt.2008.080412.

110. Cho JH, Chang SA, Kwon HS, Choi YH, Ko SH, Moon SD, et al. Long-term effect of the Internet-based glucose monitoring system on HbA1c reduction and glucose stability: a 30-month follow-up study for diabetes management with a ubiquitous medical care system. Diabetes Care. 2006 Dec;29(12):2625-31. PMID: 17130195.

111. Choi JY, Kim KI, Kim CH. Effect of home blood pressure monitoring for blood pressure control in hypertensive patients taking multiple antihypertensive medications including fimasartan (the FORTE study). Clin. 2020 Dec 15;26(1):24. PMID: 33317628. doi: https://dx.doi.org/10.1186/s40885-020-00154-y.

112. Choi WY, Kim CH, Lee OG. Effects of brief smoking cessation education with expiratory carbon monoxide measurement on level of motivation to quit smoking. Korean J Fam Med. 2013 May;34(3):190-8. PMID: 23730486. doi: https://dx.doi.org/10.4082/kjfm.2013.34.3.190.

113. Christensen KD, Roberts JS, Whitehouse PJ, Royal CDM, Obisesan TO, Cupples LA, et al. Disclosing pleiotropic effects during genetic risk assessment for Alzheimer disease. Annals of Internal Medicine. 2016 Feb 2;164(3):155-63. PMID: 26810768. doi: 10.7326/M15-0187.

114. Ciampolini M, Bianchi R. Training to estimate blood glucose and to form associations with initial hunger. Nutrition & metabolism. 2006 Dec 2006;3. doi: 10.1186/1743-7075-3-42.

115. Cioe PA, Merrill JE, Gordon REF, Guthrie KM, Freiberg M, Williams DM, et al. Personalized feedback improves cardiovascular risk perception and physical activity levels in persons with HIV: results of a pilot randomized clinical trial. AIDS care - psychological and socio-medical aspects of AIDS/HIV. 2021. PMID: CN-02245664. doi: 10.1080/09540121.2021.1874271.

116. Coffin PO, Santos GM, Colfax G, Das M, Matheson T, DeMicco E, et al. Adapted personalized cognitive counseling for episodic substance-using men who have sex with men: a randomized controlled trial. AIDS and behavior. 2014;18(7):1390‐400. PMID: CN-01044040. doi: 10.1007/s10461-014-0712-4.

117. Cohen AN, Chinman MJ, Hamilton AB, Whelan F, Young AS. Using patient-facing kiosks to support quality improvement at mental health clinics. Med Care. 2013 Mar;51(3 Suppl 1):S13-20. PMID: 23407006. doi: https://dx.doi.org/10.1097/MLR.0b013e31827da859.

118. Cohen SJ, Weinberger MH, Fineberg NS, Miller JZ, Grim CE, Luft FC. The effect of a household partner and home urine monitoring on adherence to a sodium restricted diet. Soc Sci Med. 1991;32(9):1057-61. PMID: 2047898.

119. Collins SE, Carey KB, Sliwinski MJ. Mailed personalized normative feedback as a brief intervention for at-risk college drinkers. J. 2002 Sep;63(5):559-67. PMID: 12380852.

120. Conroy MB, McTigue KM, Bryce CL, Tudorascu D, Gibbs BB, Arnold J, et al. Effect of Electronic Health Record-Based Coaching on Weight Maintenance: A Randomized Trial. Annals of Internal Medicine. 2019 12 03;171(11):777-84. PMID: 31711168. doi: https://dx.doi.org/10.7326/M18-3337.

121. Cope GF, Nayyar P, Holder R. Feedback from a point-of-care test for nicotine intake to reduce smoking during pregnancy. Ann Clin Biochem. 2003 Nov;40(Pt 6):674-9. PMID: 14629807.

122. Cosson E, Hamo-Tchatchouang E, Dufaitre-Patouraux L, Attali JR, Paries J, Schaepelynck-Belicar P. Multicentre, randomised, controlled study of the impact of continuous sub-cutaneous glucose monitoring (GlucoDay) on glycaemic control in type 1 and type 2 diabetes patients. Diabetes Metab. 2009 Sep;35(4):312-8. PMID: 19560388. doi: https://dx.doi.org/10.1016/j.diabet.2009.02.006.

123. Coventry P, Bower P, Blakemore A, Baker E, Hann M, Li J, et al. Satisfaction with a digitally-enabled telephone health coaching intervention for people with non-diabetic hyperglycaemia. npj digit. 2019;2:5. PMID: 31304355. doi: https://dx.doi.org/10.1038/s41746-019-0080-6.

124. Cox DJ, Banton T, Moncrief M, Conaway M, Diamond A, Holmes V, et al. Glycemic excursion minimization in the management of type 2 diabetes: a novel intervention tested in a randomized clinical trial. BMJ open diabetes res. 2020 12;8(2):12. PMID: 33328160. doi: https://dx.doi.org/10.1136/bmjdrc-2020-001795.

125. Cox DJ, Taylor AG, Singh H, Moncrief M, Diamond A, Yancy WS, et al. Glycemic load, exercise, and monitoring blood glucose (GEM): A paradigm shift in the treatment of type 2 diabetes mellitus. Diabetes Res Clin Pract. 2016 Jan;111:28-35. PMID: 26556234. doi: https://dx.doi.org/10.1016/j.diabres.2015.10.021.

126. Cox DJ, Kovatchev B, Koev D, Koeva L, Dachev S, Tcharaktchiev D, et al. Hypoglycemia anticipation, awareness and treatment training (HAATT) reduces occurrence of severe hypoglycemia among adults with type 1 diabetes mellitus. Int J Behav Med. 2004;11(4):212-8. PMID: 15657021.

127. Cram P, Wolinsky FD, Lou Y, Edmonds SW, Hall SF, Roblin DW, et al. Patient-activation and guideline-concordant pharmacological treatment after bone density testing: the PAADRN randomized controlled trial. Osteoporos Int. 2016 12;27(12):3513-24. PMID: 27363400.

128. Cram P, Schlechte J, Christensen A. A randomized trial to assess the impact of direct reporting of DXA scan results to patients on quality of osteoporosis care. J Clin Densitom. 2006 Oct-Dec;9(4):393-8. PMID: 17097523.

129. Crane MM. Improving men's health through weight control: Randomized trials testing recruitment messaging and a novel weight loss intervention: ProQuest Information & Learning; 2016.

130. Crowther CA, Hiller JE, Moss JR, McPhee AJ, Jeffries WS, Robinson JS, et al. Effect of treatment of gestational diabetes mellitus on pregnancy outcomes. N Engl J Med. 2005 Jun 16;352(24):2477-86. PMID: 15951574.

131. Cuffee YL, Sciamanna C, Gerin W, Lehman E, Cover L, Johnson AA, et al. The Effectiveness of Home Blood Pressure on 24-Hour Blood Pressure Control: A Randomized Controlled Trial. Am J Hypertens. 2019 01 15;32(2):186-92. PMID: 30371759. doi: https://dx.doi.org/10.1093/ajh/hpy160.

132. D'Eramo GA. A comparison of intensity of educational intervention on knowledge, attitude, weight and metabolic control in obese individuals with Type II non-insulin dependent diabetes mellitus [Ed.D.]. Ann Arbor: Teachers College, Columbia University; 1987.

133. Dale KS, McAuley KA, Taylor RW, Williams SM, Farmer VL, Hansen P, et al. Determining optimal approaches for weight maintenance: a randomized controlled trial. Cmaj. 2009 May 12;180(10):E39-46. PMID: 19433812. doi: https://dx.doi.org/10.1503/cmaj.080974.

134. Daley A, Jolly K, Madigan C, Griffin R, Roalfe A, Lewis A, et al. A brief behavioural intervention to promote regular self-weighing to prevent weight regain after weight loss: a RCT. NIHR Journals Library. 2019 4;4:4. PMID: 31042335. doi: https://dx.doi.org/10.3310/phr07070.

135. Daley A, Jolly K, Jebb SA, Roalfe A, Mackilllop L, Lewis A, et al. Effectiveness of a behavioural intervention involving regular weighing and feedback by community midwives within routine antenatal care to prevent excessive gestational weight gain: POPS2 randomised controlled trial. BMJ Open. 2019 09 17;9(9):e030174. PMID: 31530608. doi: https://dx.doi.org/10.1136/bmjopen-2019-030174.

136. Dalfó i Baqué A, Capillas Peréz R, Guarch Rocarias M, Figueras Sabater M, Ylla-Català Passola A, Balañá Vilanova M, et al. [Effectiveness of self-measurement of blood pressure in patients with hypertension: the Dioampa study]. Aten Primaria. 2005 Mar 31;35(5):233-7. PMID: 15802109. doi: 10.1157/13072786 10.1157/13072786.

137. Damschroder LJ, Buis LR, McCant FA, Kim HM, Evans R, Oddone EZ, et al. Effect of Adding Telephone-Based Brief Coaching to an mHealth App (Stay Strong) for Promoting Physical Activity Among Veterans: Randomized Controlled Trial. Journal of Medical Internet Research. 2020 08 04;22(8):e19216. PMID: 32687474. doi: https://dx.doi.org/10.2196/19216.

138. Dar-Nimrod I, Zuckerman M, Duberstein PR. The effects of learning about one's own genetic susceptibility to alcoholism: a randomized experiment. Genet Med. 2013 Feb;15(2):132-8. PMID: 22935722. doi: https://dx.doi.org/10.1038/gim.2012.111.

139. Das D, Menon I, Gupta R, Sharma A, Ahsan I, Ashraf A. Comparison of Interventional Methods to Motivate and Change the Behavioural Stage of Smokers to Quit Smoking- A Hospital Based Randomised Controlled Trial. Asian Pac J Cancer Prev. 2021 Mar 01;22(3):711-7. PMID: 33773533. doi: https://dx.doi.org/10.31557/APJCP.2021.22.3.711.

140. Davidson TM, McGillicuddy J, Mueller M, Brunner-Jackson B, Favella A, Anderson A, et al. Evaluation of an mHealth Medication Regimen Self-Management Program for African American and Hispanic Uncontrolled Hypertensives. J. 2015 Nov 17;5(4):389-405. PMID: 26593951. doi: https://dx.doi.org/10.3390/jpm5040389.

141. Davison WJ, Myint PK, Clark AB, Kim LG, Wilson EC, Langley M, et al. Does self-monitoring and self-management of blood pressure after stroke or transient ischemic attack improve control? TEST-BP, a randomized controlled trial. Am Heart J. 2018 09;203:105-8. PMID: 30060882. doi: https://dx.doi.org/10.1016/j.ahj.2018.06.002.

142. Dawes MG, Kaczorowski J, Swanson G, Hickey J, Karwalajtys T. The effect of a patient education booklet and BP 'tracker' on knowledge about hypertension. A randomized controlled trial. Fam Pract. 2010 Oct;27(5):472-8. PMID: 20631056. doi: https://dx.doi.org/10.1093/fampra/cmq048.

143. Dejesus RS, Chaudhry R, Leutink DJ, Hinton MA, Cha SS, Stroebel RJ. Effects of efforts to intensify management on blood pressure control among patients with type 2 diabetes mellitus and hypertension: a pilot study. Vasc Health Risk Manag. 2009;5:705-11. PMID: 19756162.

144. Deka P, Pozehl B, Williams MA, Norman JF, Khazanchi D, Pathak D. MOVE-HF: an internet-based pilot study to improve adherence to exercise in patients with heart failure. European Journal of Cardiovascular Nursing. 2019;18(2):122-31. PMID: 30129790. doi: 10.1177/1474515118796613.

145. DeLeon E. Access to a home monitor and hypertension among Mexican American women: A randomized controlled trial: ProQuest Information & Learning; 2010.

146. Dennison L, Morrison L, Lloyd S, Phillips D, Stuart B, Williams S, et al. Does brief telephone support improve engagement with a web-based weight management intervention? Randomized controlled trial. Journal of Medical Internet Research. 2014 Mar 28;16(3):e95. PMID: 24681761. doi: https://dx.doi.org/10.2196/jmir.3199.

147. Desai MA, Okal DO, Rose CE, Ndivo R, Oyaro B, Otieno FO, et al. Effect of point-of-care CD4 cell count results on linkage to care and antiretroviral initiation during a home-based HIV testing campaign: a non-blinded, cluster-randomised trial. Lancet HIV. 2017 09;4(9):e393-e401. PMID: 28579225. doi: https://dx.doi.org/10.1016/S2352-3018(17)30091-7.

148. Di Bartolo P, Nicolucci A, Cherubini V, Iafusco D, Scardapane M, Rossi MC. Young patients with type 1 diabetes poorly controlled and poorly compliant with self-monitoring of blood glucose: can technology help? Results of the i-NewTrend randomized clinical trial. Acta Diabetol. 2017 04;54(4):393-402. PMID: 28138788. doi: https://dx.doi.org/10.1007/s00592-017-0963-4.

149. Dierk JM, Conradt M, Schlumberger P, Rauh E, Albohn C, Hinney A, et al. Genetic aspects in obesity counselling - Effects on body acceptance and subjective well-being. Verhaltenstherapie. 2006;16(3):193‐200. PMID: CN-00613205. doi: 10.1159/000094991.

150. Digby J, Carroll RE, Chambers JA, Steele RJC. The impact of hypothetical PErsonalised Risk Information on informed choice and intention to undergo Colorectal Cancer screening colonoscopy in Scotland (PERICCS)—a randomised controlled trial. BMC Medicine. 2020;18(1). PMID: 33076932. doi: 10.1186/s12916-020-01750-3.

151. Doets EL, e Hoogh IM, Holthuysen N, Wopereis S, Verain MCD, van den Puttelaar J, et al. Beneficial effect of personalized lifestyle advice compared to generic advice on wellbeing among Dutch seniors—An explorative study. Physiology & Behavior. 2019;210. PMID: 2019-53730-001. doi: 10.1016/j.physbeh.2019.112642.

152. Doheny MO, Sedlak CA, Hall RJ, Estok PJ. Structural model for osteoporosis preventing behavior in men. Am j. 2010 Dec;4(4):334-43. PMID: 20413383. doi: https://dx.doi.org/10.1177/1557988309351953.

153. Dorough AE, Winett RA, Anderson ES, Davy BM, Martin EC, Hedrick V. DASH to wellness: emphasizing self-regulation through e-health in adults with prehypertension. Health Psychology. 2014 Mar;33(3):249-54. PMID: 23181455. doi: https://dx.doi.org/10.1037/a0030483.

154. Downs DS, Savage JS, Rivera DE, Pauley AM, Leonard KS, Hohman EE, et al. Adaptive, behavioral intervention impact on weight gain, physical activity, energy intake, and motivational determinants: results of a feasibility trial in pregnant women with overweight/obesity. J Behav Med. 2021 May 05;05:05. PMID: 33954853. doi: https://dx.doi.org/10.1007/s10865-021-00227-9.

155. Drion I, Pameijer LR, van Dijk PR, Groenier KH, Kleefstra N, Bilo HJ. The Effects of a Mobile Phone Application on Quality of Life in Patients With Type 1 Diabetes Mellitus: A Randomized Controlled Trial. J Diabetes Sci Technol. 2015 May 11;9(5):1086-91. PMID: 25963412. doi: https://dx.doi.org/10.1177/1932296815585871.

156. Du Y, Dennis B, Rhodes SL, Sia M, Ko J, Jiwani R, et al. Technology-Assisted Self-Monitoring of Lifestyle Behaviors and Health Indicators in Diabetes: Qualitative Study. JMIR Diabetes. 2020 Aug 28;5(3):e21183. PMID: 32857056. doi: https://dx.doi.org/10.2196/21183.

157. Dubbert PM, Cushman WC, Meydrech EF, Rowland AK, Maury P. Effects of dietary instruction and sodium excretion feedback in hypertension clinic patients. Behavior Therapy. 1995;26(4):721-32.

158. Duncan MJ, Fenton S, Brown WJ, Collins CE, Glozier N, Kolt GS, et al. Efficacy of a Multi-component m-Health Weight-loss Intervention in Overweight and Obese Adults: A Randomised Controlled Trial. Int J Environ Res Public Health. 2020 08 26;17(17):26. PMID: 32859100. doi: https://dx.doi.org/10.3390/ijerph17176200.

159. Duncan S, Goodyear-Smith F, McPhee J, Zinn C, Grøntved A, Schofield G. Family-centered brief intervention for reducing obesity and cardiovascular disease risk: A randomized controlled trial. Obesity. 2016;24(11):2311-8. PMID: 27616217. doi: 10.1002/oby.21602.

160. Earp JA, Ory MG, Strogatz DS. The effects of family involvement and practitioner home visits on the control of hypertension. American Journal of Public Health. 1982 Oct;72(10):1146-54. PMID: 7114339.

161. Eganyan RA, Kalinina AM, Gorny BE, Izmailova OV, Komkov DS, Kushunina DV, et al. The dynamics of nutrition structure of overweight and obese people during preventive counseling and remote monitoring as part of the international russian-japanese study «tackle obesity and metabolic syndrome outcome by diet, activities and checking bw intervention (Rj-tomodachi). Profilakticheskaya Meditsina. 2020;23(3):119-30. doi: 10.17116/profmed202023031119.

162. Ehrhardt NM, Chellappa M, Walker MS, Fonda SJ, Vigersky RA. The effect of real-time continuous glucose monitoring on glycemic control in patients with type 2 diabetes mellitus. J Diabetes Sci Technol. 2011 May 1;5(3):668-75. PMID: 21722581. doi: 10.1177/193229681100500320.

163. Elixhauser A, Eisen SA, Romeis JC, Homan SM. The effects of monitoring and feedback on compliance. Med Care. 1990 Oct;28(10):882-93. PMID: 2232919.

164. Elton PJ, Ryman A, Hammer M, Page F. Randomised controlled trial in northern England of the effect of a person knowing their own serum cholesterol concentration. J Epidemiol Community Health. 1994 Feb;48(1):22-5. PMID: 8138763.

165. Emerson JF, Welch M, Rossman WE, Carek S, Ludden T, Templin M, et al. A Multidisciplinary Intervention Utilizing Virtual Communication Tools to Reduce Health Disparities: A Pilot Randomized Controlled Trial. Int J Environ Res Public Health. 2015 Dec 22;13(1):ijerph13010031. PMID: 26703661. doi: https://dx.doi.org/10.3390/ijerph13010031.

166. Emmons KM, Geller AC, Puleo E, Savadatti SS, Hu SW, Gorham S, et al. Skin cancer education and early detection at the beach: a randomized trial of dermatologist examination and biometric feedback. J Am Acad Dermatol. 2011 Feb;64(2):282-9. PMID: 21163550. doi: https://dx.doi.org/10.1016/j.jaad.2010.01.040.

167. Engberg M, Christensen B, Karlsmose B, Lous J, Lauritzen T. General health screenings to improve cardiovascular risk profiles: A randomized controlled trial in general practice with 5-year follow-up. Journal of Family Practice. 2002;51(6):546-52. PMID: 12100779.

168. Estok PJ, Sedlak CA, Doheny MO, Hall R. Structural model for osteoporosis preventing behavior in postmenopausal women. Nurs Res. 2007 May-Jun;56(3):148-58. PMID: 17495570.

169. Fanning J, Brooks AK, Ip E, Nicklas BJ, Rejeski WJ, Nesbit B, et al. A Mobile Health Behavior Intervention to Reduce Pain and Improve Health in Older Adults With Obesity and Chronic Pain: The MORPH Pilot Trial. Front. 2020 Dec;2. PMID: 33817686. doi: https://dx.doi.org/10.3389/fdgth.2020.598456.

170. Faridi Z, Liberti L, Shuval K, Northrup V, Ali A, Katz DL. Evaluating the impact of mobile telephone technology on type 2 diabetic patients' self-management: the NICHE pilot study. J Eval Clin Pract. 2008 Jun;14(3):465-9. PMID: 18373577. doi: https://dx.doi.org/10.1111/j.1365-2753.2007.00881.x.

171. Farmer A, Wade A, Goyder E, Yudkin P, French D, Craven A, et al. Impact of self monitoring of blood glucose in the management of patients with non-insulin treated diabetes: Open parallel group randomised trial. BMJ: British Medical Journal. 2007;335(7611):132-. PMID: 2007-12053-001. doi: 10.1136/bmj.39247.447431.BE.

172. Fathima FN, George N, George M, Mathew SS, Rajitha M, Agrawal T, et al. Effectiveness of Color Coded Diabetic Control Monitoring Charts among Elderly Diabetics Attending Outreach Primary Care Geriatric Clinics in Rural Karnataka: An Open Label Randomized Control Trial. Indian J. 2019 Jan-Mar;44(1):39-43. PMID: 30983712. doi: https://dx.doi.org/10.4103/ijcm.IJCM_231_18.

173. Fehring RJ, Schneider M, Raviele K, Rodriguez D, Pruszynski J. Randomized comparison of two Internet-supported fertility-awareness-based methods of family planning. Contraception. 2013 Jul;88(1):24-30. PMID: 23153900. doi: https://dx.doi.org/10.1016/j.contraception.2012.10.010.

174. Fielding JE, Knight K, Mason T, Klesges RC, Pelletier KR. Evaluation of the IMPACT blood pressure program. J Occup Med. 1994 Jul;36(7):743-6. PMID: 7931739.

175. Fischer HH, Eisert SL, Durfee MJ, Moore SL, Steele AW, McCullen K, et al. The impact of tailored diabetes registry report cards on measures of disease control: a nested randomized trial. BMC Med Inf Decis Mak. 2011 Feb 17;11:12. PMID: 21329495. doi: https://dx.doi.org/10.1186/1472-6947-11-12.

176. Fleming M, Brown R, Brown D. The efficacy of a brief alcohol intervention combined with %CDT feedback in patients being treated for type 2 diabetes and/or hypertension. J. 2004 Sep;65(5):631-7. PMID: 15536773.

177. Fontbonne A, Billault B, Acosta M, Percheron C, Varenne P, Besse A, et al. Is glucose self-monitoring beneficial in non-insulin-treated diabetic patients? Results of a randomized comparative trial. Diabete Metab. 1989 Sep-Oct;15(5):255-60. PMID: 2630378.

178. Foulds J, Veldheer S, Hrabovsky S, Yingst J, Sciamanna C, Chen G, et al. The effect of motivational lung age feedback on short-term quit rates in smokers seeking intensive group treatment: A randomized controlled pilot study. Drug Alcohol Depend. 2015 Aug 01;153:271-7. PMID: 26051163. doi: https://dx.doi.org/10.1016/j.drugalcdep.2015.05.007.

179. Fountoulakis S, Papanastasiou L, Gryparis A, Markou A, Piaditis G. Impact and duration effect of telemonitoring on EtabA1c, BMI and cost in insulin-treated Diabetes Mellitus patients with inadequate glycemic control: A randomized controlled study. Hormones. 2015 Oct-Dec;14(4):632-43. PMID: 26188234. doi: https://dx.doi.org/10.14310/horm.2002.1603.

180. Franciosi M, Lucisano G, Pellegrini F, Cantarello A, Consoli A, Cucco L, et al. ROSES: role of self-monitoring of blood glucose and intensive education in patients with Type 2 diabetes not receiving insulin. A pilot randomized clinical trial. Diabet Med. 2011 Jul;28(7):789-96. PMID: 21342243. doi: https://dx.doi.org/10.1111/j.1464-5491.2011.03268.x.

181. Frias J, Virdi N, Raja P, Kim Y, Savage G, Osterberg L. Effectiveness of Digital Medicines to Improve Clinical Outcomes in Patients with Uncontrolled Hypertension and Type 2 Diabetes: Prospective, Open-Label, Cluster-Randomized Pilot Clinical Trial. Journal of Medical Internet Research. 2017 07 11;19(7):e246. PMID: 28698169. doi: https://dx.doi.org/10.2196/jmir.7833.

182. Frie K, Hartmann-Boyce J, Jebb SA, Aveyard P. Effectiveness of a self-regulation intervention for weight loss: A randomized controlled trial. British Journal of Health Psychology. 2020 09;25(3):652-76. PMID: 32489005. doi: https://dx.doi.org/10.1111/bjhp.12436.

183. Friedman RH, Kazis LE, Jette A, Smith MB, Stollerman J, Torgerson J, et al. A telecommunications system for monitoring and counseling patients with hypertension. Impact on medication adherence and blood pressure control. Am J Hypertens. 1996 Apr;9(4 Pt 1):285-92. PMID: 8722429.

184. Fries JF, Bloch DA, Harrington H, Richardson N, Beck R. Two-year results of a randomized controlled trial of a health promotion program in a retiree population: the Bank of America Study. Am J Med. 1993 May;94(5):455-62. PMID: 8498389. doi: https://dx.doi.org/10.1016/0002-9343(93)90078-4.

185. Fu SN, Dao MC, Luk W, Lam MCH, Ho ISF, Cheung SK, et al. A cluster-randomized study on the Risk Assessment and Management Program for home blood pressure monitoring in an older population with inadequate health literacy. J Clin Hypertens (Greenwich). 2020 09;22(9):1565-76. PMID: 32810355. doi: https://dx.doi.org/10.1111/jch.13987.

186. Fucito LM, DeMartini KS, Hanrahan TH, Yaggi HK, Heffern C, Redeker NS. Using Sleep Interventions to Engage and Treat Heavy-Drinking College Students: A Randomized Pilot Study. Alcohol Clin Exp Res. 2017 04;41(4):798-809. PMID: 28118486. doi: https://dx.doi.org/10.1111/acer.13342.

187. Fukuoka Y, Gay CL, Joiner KL, Vittinghoff E. A Novel Diabetes Prevention Intervention Using a Mobile App. American Journal of Preventive Medicine. 2015;49(2):223-37. doi: 10.1016/j.amepre.2015.01.003.

188. Fung CS, Wong WC, Wong CK, Lee A, Lam CL. Home blood pressure monitoring--a trial on the effect of a structured education program. Aust Fam Physician. 2013 Apr;42(4):233-7. PMID: 23550251.

189. Furler J, O'Neal D, Speight J, Blackberry I, Manski-Nankervis JA, Thuraisingam S, et al. Use of professional-mode flash glucose monitoring, at 3-month intervals, in adults with type 2 diabetes in general practice (GP-OSMOTIC): a pragmatic, open-label, 12-month, randomised controlled trial. Lancet Diabetes Endocrinol. 2020 01;8(1):17-26. PMID: 31862147. doi: https://dx.doi.org/10.1016/S2213-8587(19)30385-7.

190. Gabriele JM, Carpenter BD, Tate DF, Fisher EB. Directive and nondirective e-coach support for weight loss in overweight adults. Ann Behav Med. 2011 Apr;41(2):252-63. PMID: 21108032. doi: https://dx.doi.org/10.1007/s12160-010-9240-2.

191. Gaillard T, Amponsah G, Osei K. Patient-Centered Community Diabetes Education Program Improves Glycemic Control in African-American Patients with Poorly Controlled Type 2 Diabetes: Importance of Point of Care Metabolic Measurements. J Natl Black Nurses Assoc. 2015 Jul;26(1):50-7. PMID: 26371360.

192. Gajecki M, Berman AH, Sinadinovic K, Rosendahl I, Andersson C. Mobile phone brief intervention applications for risky alcohol use among university students: a randomized controlled study. Addict Sci Clin Pract. 2014 Jul 02;9:11. PMID: 24985342. doi: https://dx.doi.org/10.1186/1940-0640-9-11.

193. Gallagher EM, Brunt H. Head over heels: Impact of a health promotion program to reduce falls in the elderly. Canadian Journal on Aging. 1996;15(1):84-96. doi: 10.1017/S0714980800013301.

194. Gamage DG, Riddell MA, Joshi R, Thankappan KR, Chow CK, Oldenburg B, et al. Effectiveness of a scalable group-based education and monitoring program, delivered by health workers, to improve control of hypertension in rural India: A cluster randomised controlled trial. PLoS Med. 2020 01;17(1):e1002997. PMID: 31895945. doi: https://dx.doi.org/10.1371/journal.pmed.1002997.

195. Garcia AA, Brown SA, Horner SD, Zuniga J, Arheart KL. Home-based diabetes symptom self-management education for Mexican Americans with type 2 diabetes. Health Education Research. 2015 Jun;30(3):484-96. PMID: 25953971. doi: https://dx.doi.org/10.1093/her/cyv018.

196. Garcia DO. Feasibility of a campaign intervention compared to a standard behavioral weight loss intervention in overweight and obese adults: ProQuest Information & Learning; 2014.

197. Garcia de la Torre N, Duran A, Del Valle L, Fuentes M, Barca I, Martin P, et al. Early management of type 2 diabetes based on a SMBG strategy: the way to diabetes regression--the St Carlos study : a 3-year, prospective, randomized, clinic-based, interventional study with parallel groups. Acta Diabetol. 2013 Aug;50(4):607-14. PMID: 23532298. doi: https://dx.doi.org/10.1007/s00592-013-0467-9.

198. Garcia-Hernandez P, Carranza-Lira S, Motta-Martinez E. [Monthly ibandronate attachment to Mexican and Chilean women with osteoporosis, with or without a biofeedback strategy]. Ginecol Obstet Mex. 2010 Jun;78(6):322-8. PMID: 20939245.

199. Gayman C, Anderson K, Pietras C. Saliva cotinine as a measure of smoking abstinence in contingency management—A feasibility study. The Psychological Record. 2017;67(2):261-72. PMID: 2017-19018-001. doi: 10.1007/s40732-017-0240-5.

200. Gemson DH, Sloan RP. Efficacy of computerized health risk appraisal as part of a periodic health examination at the worksite. American Journal of Health Promotion. 1995 Jul-Aug;9(6):462-6. PMID: 10150537.

201. Gidlow CJ, Ellis NJ, Riley V, Chadborn T, Bunten A, Iqbal Z, et al. Randomised controlled trial comparing uptake of NHS Health Check in response to standard letters, risk-personalised letters and telephone invitations. BMC Public Health. 2019 Feb 21;19(1):224. PMID: 30791884. doi: https://dx.doi.org/10.1186/s12889-019-6540-8.

202. Gimbel RW, Rennert LM, Crawford P, Little JR, Truong K, Williams JE, et al. Enhancing Patient Activation and Self-Management Activities in Patients With Type 2 Diabetes Using the US Department of Defense Mobile Health Care Environment: Feasibility Study. Journal of Medical Internet Research. 2020 05 26;22(5):e17968. PMID: 32329438. doi: https://dx.doi.org/10.2196/17968.

203. Glanz K, Volpicelli K, Kanetsky PA, Ming ME, Schuchter LM, Jepson C, et al. Melanoma genetic testing, counseling, and adherence to skin cancer prevention and detection behaviors. Cancer Epidemiol Biomarkers Prev. 2013 Apr;22(4):607-14. PMID: 23392000. doi: https://dx.doi.org/10.1158/1055-9965.EPI-12-1174.

204. Glasgow RE, Kurz D, King D, Dickman JM, Faber AJ, Halterman E, et al. Twelve-month outcomes of an Internet-based diabetes self-management support program. Patient Educ Couns. 2012 Apr;87(1):81-92. PMID: 21924576. doi: https://dx.doi.org/10.1016/j.pec.2011.07.024.

205. Glasgow RE, Klesges RC, Godding PR, Gegelman R. Controlled smoking, with or without carbon monoxide feedback, as an alternative for chronic smokers. Behavior Therapy. 1983;14(3):386-97.

206. Gleason-Comstock JA, Streater A, Jen KL, Artinian NT, Timmins J, Baker S, et al. Consumer health information technology in an adult public health primary care clinic: a heart health education feasibility study. Patient Educ Couns. 2013 Dec;93(3):464-71. PMID: 23948646. doi: https://dx.doi.org/10.1016/j.pec.2013.07.010.

207. Glynn SM, Gruder CL, Jegerski JA. Effects of biochemical validation of self-reported cigarette smoking on treatment success and on misreporting abstinence. Health Psychology. 1986;5(2):125-36. PMID: 1987-32516-001. doi: 10.1037/0278-6133.5.2.125.

208. Godin G, Desharnais R, Jobin J, Cook J. The impact of physical fitness and health-age appraisal upon exercise intentions and behavior. J Behav Med. 1987 Jun;10(3):241-50. PMID: 3612781.

209. Godino JG, van Sluijs EM, Marteau TM, Sutton S, Sharp SJ, Griffin SJ. Lifestyle Advice Combined with Personalized Estimates of Genetic or Phenotypic Risk of Type 2 Diabetes, and Objectively Measured Physical Activity: A Randomized Controlled Trial. PLoS Med. 2016 Nov;13(11):e1002185. PMID: 27898672. doi: https://dx.doi.org/10.1371/journal.pmed.1002185.

210. Godwin M, Lam M, Birtwhistle R, Delva D, Seguin R, Casson I, et al. A primary care pragmatic cluster randomized trial of the use of home blood pressure monitoring on blood pressure levels in hypertensive patients with above target blood pressure. Fam Pract. 2010 Apr;27(2):135-42. PMID: 20032170. doi: https://dx.doi.org/10.1093/fampra/cmp094.

211. Gokee LaRose J, Tate DF, Gorin AA, Wing RR. Preventing weight gain in young adults: a randomized controlled pilot study. American Journal of Preventive Medicine. 2010 Jul;39(1):63-8. PMID: 20537843. doi: https://dx.doi.org/10.1016/j.amepre.2010.03.011.

212. Gokee-Larose J, Gorin AA, Wing RR. Behavioral self-regulation for weight loss in young adults: a randomized controlled trial. Int. 2009 Feb 16;6:10. PMID: 19220909. doi: https://dx.doi.org/10.1186/1479-5868-6-10.

213. Goldstein A, Horns WH, Hansteen RW. Is on-site urine testing of therapeutic value in a methadone treatment program? Int J Addict. 1977 Sep;12(6):717-28. PMID: 591135.

214. Gomel M, Oldenburg B, Simpson JM, Owen N. Work-site cardiovascular risk reduction: a randomized trial of health risk assessment, education, counseling, and incentives. American Journal of Public Health. 1993 Sep;83(9):1231-8. PMID: 8362997.

215. Gopalan A, Suttner L, Troxel AB, McDonough K, Schapira MM. Testing patient-informed approaches for visually depicting the hemoglobin A1c value to patients with poorly controlled diabetes: a randomized, controlled trial. BMC Health Serv Res. 2020 Mar 06;20(1):178. PMID: 32143649. doi: https://dx.doi.org/10.1186/s12913-020-5035-8.

216. Goulis DG, Giaglis GD, Boren SA, Lekka I, Bontis E, Balas EA, et al. Effectiveness of home-centered care through telemedicine applications for overweight and obese patients: A randomized controlled trial. International Journal of Obesity. 2004;28(11):1391-8. PMID: 2004-20477-001. doi: 10.1038/sj.ijo.0802773.

217. Gow RW, Trace SE, Mazzeo SE. Preventing weight gain in first year college students: an online intervention to prevent the "freshman fifteen". Eat. 2010 Jan;11(1):33-9. PMID: 19962118. doi: https://dx.doi.org/10.1016/j.eatbeh.2009.08.005.

218. Grant RW, O'Brien KE, Waxler JL, Vassy JL, Delahanty LM, Bissett LG, et al. Personalized genetic risk counseling to motivate diabetes prevention: a randomized trial. Diabetes Care. 2013 Jan;36(1):13-9. PMID: 22933432. doi: https://dx.doi.org/10.2337/dc12-0884.

219. Green BB, Anderson ML, Cook AJ, Catz S, Fishman PA, McClure JB, et al. e-Care for heart wellness: a feasibility trial to decrease blood pressure and cardiovascular risk. American Journal of Preventive Medicine. 2014 Apr;46(4):368-77. PMID: 24650839. doi: https://dx.doi.org/10.1016/j.amepre.2013.11.009.

220. Green BB, Cook AJ, Ralston JD, Fishman PA, Catz SL, Carlson J, et al. Effectiveness of home blood pressure monitoring, Web communication, and pharmacist care on hypertension control: a randomized controlled trial. Jama. 2008 Jun 25;299(24):2857-67. PMID: 18577730. doi: https://dx.doi.org/10.1001/jama.299.24.2857.

221. Greene J, Sacks R, Piniewski B, Kil D, Hahn JS. The impact of an online social network with wireless monitoring devices on physical activity and weight loss. J. 2013 Jul 01;4(3):189-94. PMID: 23799706. doi: https://dx.doi.org/10.1177/2150131912469546.

222. Greenwood DA, Blozis SA, Young HM, Nesbitt TS, Quinn CC. Overcoming Clinical Inertia: A Randomized Clinical Trial of a Telehealth Remote Monitoring Intervention Using Paired Glucose Testing in Adults With Type 2 Diabetes. Journal of Medical Internet Research. 2015 Jul 21;17(7):e178. PMID: 26199142. doi: https://dx.doi.org/10.2196/jmir.4112.

223. Grilo SA, Shallcross AJ, Ogedegbe G, Odedosu T, Levy N, Lehrer S, et al. Food insecurity and effectiveness of behavioral interventions to reduce blood pressure, New York City, 2012–2013. Preventing Chronic Disease: Public Health Research, Practice, and Policy. 2015;12. PMID: 2015-17900-001. doi: 10.5888/pcd12.140368.

224. Grossman JA, Arigo D, Bachman JL. Meaningful weight loss in obese postmenopausal women: a pilot study of high-intensity interval training and wearable technology. Menopause. 2018 04;25(4):465-70. PMID: 29088015. doi: https://dx.doi.org/10.1097/GME.0000000000001013.

225. Grover SA, Lowensteyn I, Joseph L, Kaouache M, Marchand S, Coupal L, et al. Patient knowledge of coronary risk profile improves the effectiveness of dyslipidemia therapy: the CHECK-UP study: a randomized controlled trial. Arch Intern Med. 2007 Nov 26;167(21):2296-303. PMID: 18039987.

226. Gu Y, Bao X, Wang Y, Meng G, Wu H, Zhang Q, et al. Effects of self-monitoring devices on blood pressure in older adults with hypertension and diabetes: a randomised controlled trial. J Epidemiol Community Health. 2020 02;74(2):137-43. PMID: 31678965. doi: https://dx.doi.org/10.1136/jech-2019-212531.

227. Guerci B, Drouin P, Grange V, Bougneres P, Fontaine P, Kerlan V, et al. Self-monitoring of blood glucose significantly improves metabolic control in patients with type 2 diabetes mellitus: the Auto-Surveillance Intervention Active (ASIA) study. Diabetes Metab. 2003 Dec;29(6):587-94. PMID: 14707887.

228. Gunawardena KC, Jackson R, Robinett I, Dhaniska L, Jayamanne S, Kalpani S, et al. The Influence of the Smart Glucose Manager Mobile Application on Diabetes Management. J Diabetes Sci Technol. 2019 01;13(1):75-81. PMID: 30264583. doi: https://dx.doi.org/10.1177/1932296818804522.

229. Guo H, Tian X, Li R, Lin J, Jin N, Wu Z, et al. Reward-based, task-setting education strategy on glycemic control and self-management for low-income outpatients with type 2 diabetes. J. 2014 Jul;5(4):410-7. PMID: 25411600. doi: https://dx.doi.org/10.1111/jdi.12152.

230. Haapala I, Barengo NC, Biggs S, Surakka L, Manninen P. Weight loss by mobile phone: a 1-year effectiveness study. Public Health Nutr. 2009 Dec;12(12):2382-91. PMID: 19323865. doi: https://dx.doi.org/10.1017/S1368980009005230.

231. Haddow JE, Knight GJ, Kloza EM, Palomaki GE, Wald NJ. Cotinine-assisted intervention in pregnancy to reduce smoking and low birthweight delivery. Br J Obstet Gynaecol. 1991 Sep;98(9):859-65. PMID: 1716979.

232. Haggerty AF, Hagemann A, Barnett M, Thornquist M, Neuhouser ML, Horowitz N, et al. A randomized, controlled, multicenter study of technology‐based weight loss interventions among endometrial cancer survivors. Obesity. 2017;25(Suppl 2):S102-S8. PMID: 2017-49485-018. doi: 10.1002/oby.22021.

233. Haggerty AF, Huepenbecker S, Sarwer DB, Spitzer J, Raggio G, Chu CS, et al. The use of novel technology-based weight loss interventions for obese women with endometrial hyperplasia and cancer. Gynecol Oncol. 2016 Feb;140(2):239-44. PMID: 26644265. doi: https://dx.doi.org/10.1016/j.ygyno.2015.11.033.

234. Hagglund E, Lynga P, Frie F, Ullman B, Persson H, Melin M, et al. Patient-centred home-based management of heart failure. Findings from a randomised clinical trial evaluating a tablet computer for self-care, quality of life and effects on knowledge. Scand Cardiovasc J. 2015 Aug;49(4):193-9. PMID: 25968968. doi: https://dx.doi.org/10.3109/14017431.2015.1035319.

235. Hajek P, Taylor TZ, Mills P. Brief intervention during hospital admission to help patients to give up smoking after myocardial infarction and bypass surgery: randomised controlled trial. Bmj. 2002 Jan 12;324(7329):87-9. PMID: 11786452.

236. Hajek P, West R, Lee A, Foulds J, Owen L, Eiser JR, et al. Randomized controlled trial of a midwife-delivered brief smoking cessation intervention in pregnancy. Addiction. 2001 Mar;96(3):485-94. PMID: 11255587.

237. Hales S, Turner-McGrievy GM, Wilcox S, Fahim A, Davis RE, Huhns M, et al. Social networks for improving healthy weight loss behaviors for overweight and obese adults: A randomized clinical trial of the social pounds off digitally (Social POD) mobile app. Int J Med Inf. 2016 10;94:81-90. PMID: 27573315. doi: https://dx.doi.org/10.1016/j.ijmedinf.2016.07.003.

238. Halimi S, Charpentier G, Grimaldi A, Grenier JL, Baut F, Germain B, et al. Effect on compliance, acceptability of blood glucose self-monitoring and HbA(1c) of a self-monitoring system developed according to patient's wishes. The ACCORD study. Diabetes Metab. 2001 Dec;27(6):681-7. PMID: 11852377.

239. Hall SM, Bass A, Monroe J. Continued contact and monitoring as follow-up strategies: a long-term study of obesity treatment. Addictive Behaviors. 1978;3(2):139-47. PMID: 717093.

240. Hall SM, Hall RG, Borden BL, Hanson RW. Follow-up strategies in the behavioral treatment of overweight. Behav Res Ther. 1975 Jun;13(2-3):167-72. PMID: 1164372.

241. Hall SM. Self-control and therapist control in the behavioral treatment of overweight women. Behaviour Research and Therapy. 1972;10(1):59-68. PMID: 1973-11621-001. doi: 10.1016/0005-7967(72)90008-3.

242. Hallam SM. Enhancing self-efficacy and pelvic floor muscle exercise adherence through semg biofeedback: a randomised study [D.Prof.]. Ann Arbor: University of Salford (United Kingdom); 2012.

243. Halle M, Rohling M, Banzer W, Braumann KM, Kempf K, McCarthy D, et al. Meal replacement by formula diet reduces weight more than a lifestyle intervention alone in patients with overweight or obesity and accompanied cardiovascular risk factors-the ACOORH trial. Eur J Clin Nutr. 2021 Apr;75(4):661-9. PMID: 33128036. doi: https://dx.doi.org/10.1038/s41430-020-00783-4.

244. Hanley J, Fairbrother P, Krishan A, McCloughan L, Padfield P, Paterson M, et al. Mixed methods feasibility study for a trial of blood pressure telemonitoring for people who have had stroke/transient ischaemic attack (TIA). Trials. 2015 Mar 25;16:117. PMID: 25873155. doi: https://dx.doi.org/10.1186/s13063-015-0628-y.

245. Hanlon P, McEwen J, Carey L, Gilmour H, Tannahill C, Tannahill A, et al. Health checks and coronary risk: further evidence from a randomised controlled trial. Bmj. 1995 Dec 16;311(7020):1609-13. PMID: 8555805.

246. Hansen CR, Perrild H, Koefoed BG, Zander M. Video consultations as add-on to standard care among patients with type 2 diabetes not responding to standard regimens: a randomized controlled trial. Eur. 2017 Jun;176(6):727-36. PMID: 28325823. doi: https://dx.doi.org/10.1530/EJE-16-0811.

247. Harashima S, Fukushima T, Sasaki M, Nishi Y, Fujimoto S, Ogura M, et al. Self-monitoring of blood glucose (SMBG) improves glycaemic control in oral hypoglycaemic agent (OHA)-treated type 2 diabetes (SMBG-OHA study). Diabetes Metab Res Rev. 2013 Jan;29(1):77-84. PMID: 23008090. doi: https://dx.doi.org/10.1002/dmrr.2363.

248. Harris R, Lowers V, Laverty L, Vernazza C, Burnside G, Brown S, et al. Comparing how patients value and respond to information on risk given in three different forms during dental check-ups: The PREFER randomised controlled trial. Trials. 2020;21(1). PMID: 31907022. doi: 10.1186/s13063-019-3824-3.

249. Hassanein M, Kumwenda MJ, Hemida K, Clark K, Roberts J, Pritchard Jones C, et al. Structured hypertension education program for people with type 2 diabetes, the SHED study. Diabetes Res Clin Pract. 2021 Mar 22;175:108773. PMID: 33766695. doi: https://dx.doi.org/10.1016/j.diabres.2021.108773.

250. Hay JL, Baguer C, Li Y, Orlow I, Berwick M. Interpretation of melanoma risk feedback in first-degree relatives of melanoma patients. Journal of Cancer Epidemiology. 2012. doi: 10.1155/2012/374842.

251. Haynes RB, Sackett DL, Gibson ES, Taylor DW, Hackett BC, Roberts RS, et al. Improvement of medication compliance in uncontrolled hypertension. Lancet. 1976 Jun 12;1(7972):1265-8. PMID: 73694.

252. Hebert PL, Sisk JE, Tuzzio L, Casabianca JM, Pogue VA, Wang JJ, et al. Nurse-led disease management for hypertension control in a diverse urban community: A randomized trial. Journal of General Internal Medicine. 2012 Jun;27(6):630-9. PMID: 22143452. doi: 10.1007/s11606-011-1924-1.

253. Heckerman CL, Brownell KD, Westlake RJ. Self and external monitoring of weight. Psychological Reports. 1978;43(2):375-8. PMID: 1980-10192-001. doi: 10.2466/pr0.1978.43.2.375.

254. Hedderson MM, Brown SD, Ehrlich SF, Tsai AL, Zhu Y, Quesenberry CP, et al. A Tailored Letter Based on Electronic Health Record Data Improves Gestational Weight Gain Among Women With Gestational Diabetes Mellitus: The Gestational Diabetes' Effects on Moms (GEM) Cluster-Randomized Controlled Trial. Diabetes Care. 2018 07;41(7):1370-7. PMID: 29669736. doi: https://dx.doi.org/10.2337/dc17-1133.

255. Hellerstedt WL, Jeffery RW. The effects of a telephone-based intervention on weight loss. American Journal of Health Promotion. 1997 Jan-Feb;11(3):177-82. PMID: 10165095.

256. Hendershot CS, Otto JM, Collins SE, Liang T, Wall TL. Evaluation of a brief web-based genetic feedback intervention for reducing alcohol-related health risks associated with ALDH2. Ann Behav Med. 2010 Aug;40(1):77-88. PMID: 20652463. doi: https://dx.doi.org/10.1007/s12160-010-9207-3.

257. Henneman L, Oosterwijk JC, van Asperen CJ, Menko FH, Ockhuysen-Vermey CF, Kostense PJ, et al. The effectiveness of a graphical presentation in addition to a frequency format in the context of familial breast cancer risk communication: a multicenter controlled trial. BMC Med Inform Decis Mak. 2013 Apr 29;13:55. PMID: 23627498. doi: 10.1186/1472-6947-13-55 10.1186/1472-6947-13-55.

258. Hermanns N, Ehrmann D, Schipfer M, Kroger J, Haak T, Kulzer B. The impact of a structured education and treatment programme (FLASH) for people with diabetes using a flash sensor-based glucose monitoring system: Results of a randomized controlled trial. Diabetes Res Clin Pract. 2019 Apr;150:111-21. PMID: 30844467. doi: https://dx.doi.org/10.1016/j.diabres.2019.03.003.

259. Hernandez-Reyes A, Camara-Martos F, Vidal A, Molina-Luque R, Moreno-Rojas R. Effects of Self-Weighing During Weight Loss Treatment: A 6-Month Randomized Controlled Trial. Front Psychol. 2020;11:397. PMID: 32210897. doi: https://dx.doi.org/10.3389/fpsyg.2020.00397.

260. Hietaranta-Luoma HL, Tahvonen R, Iso-Touru T, Puolijoki H, Hopia A. An intervention study of individual, apoE genotype-based dietary and physical-activity advice: impact on health behavior. J. 2014;7(3):161-74. PMID: 25720616.

261. Hishida A, Terazawa T, Mamiya T, Ito H, Matsuo K, Tajima K, et al. Efficacy of genotype notification to Japanese smokers on smoking cessation-An intervention study at workplace. Cancer Epidemiology. 2010 Feb;34(1):96-100. PMID: 20022836. doi: 10.1016/j.canep.2009.11.008.

262. Hiss RG, Gillard ML, Armbruster BA, McClure LA. Comprehensive evaluation of community-based diabetic patients: effect of feedback to patients and their physicians: a randomized controlled trial. Diabetes Care. 2001 Apr;24(4):690-4. PMID: 11315832.

263. Holbrook A, Pullenayegum E, Thabane L, Troyan S, Foster G, Keshavjee K, et al. Shared electronic vascular risk decision support in primary care: Computerization of Medical Practices for the Enhancement of Therapeutic Effectiveness (COMPETE III) randomized trial. Arch Intern Med. 2011 Oct 24;171(19):1736-44. PMID: 22025430. doi: https://dx.doi.org/10.1001/archinternmed.2011.471.

264. Hollands GJ, Marteau TM. The impact of using visual images of the body within a personalized health risk assessment: an experimental study. British Journal of Health Psychology. 2013 May;18(2):263-78. PMID: 23279308. doi: https://dx.doi.org/10.1111/bjhp.12016.

265. Holman RR, Dornan TL, Mayon-White V, Howard-Williams J, Orde-Peckar C, Jenkins L, et al. Prevention of deterioration of renal and sensory-nerve function by more intensive management of insulin-dependent diabetic patients. A two-year randomised prospective study. Lancet. 1983 Jan 29;1(8318):204-8. PMID: 6130244.

266. Holmen H, Torbjornsen A, Wahl AK, Jenum AK, Smastuen MC, Arsand E, et al. A Mobile Health Intervention for Self-Management and Lifestyle Change for Persons With Type 2 Diabetes, Part 2: One-Year Results From the Norwegian Randomized Controlled Trial RENEWING HEALTH. JMIR Mhealth Uhealth. 2014 Dec 11;2(4):e57. PMID: 25499872. doi: https://dx.doi.org/10.2196/mhealth.3882.

267. Homko CJ, Deeb LC, Rohrbacher K, Mulla W, Mastrogiannis D, Gaughan J, et al. Impact of a telemedicine system with automated reminders on outcomes in women with gestational diabetes mellitus. Diabetes Technol Ther. 2012 Jul;14(7):624-9. PMID: 22512287. doi: https://dx.doi.org/10.1089/dia.2012.0010.

268. Homko CJ, Santamore WP, Whiteman V, Bower M, Berger P, Geifman-Holtzman O, et al. Use of an internet-based telemedicine system to manage underserved women with gestational diabetes mellitus. Diabetes Technol Ther. 2007 Jun;9(3):297-306. PMID: 17561800.

269. Homko CJ, Sivan E, Reece EA. The impact of self-monitoring of blood glucose on self-efficacy and pregnancy outcomes in women with diet-controlled gestational diabetes. Diabetes Educ. 2002 May-Jun;28(3):435-43. PMID: 12073958.

270. Homma S, Imamura H, Nakamura T, Fujimura K, Ito Y, Maeda Y, et al. A comparative study on the effectiveness of one-way printed communication versus videophone interactive interviews on health promotion. J Telemed Telecare. 2016 Jan;22(1):56-63. PMID: 26026183. doi: https://dx.doi.org/10.1177/1357633X15587436.

271. Horne JR, Gilliland JA, O'Connor CP, Seabrook JA, Madill J. Change in Weight, BMI, and Body Composition in a Population-Based Intervention Versus Genetic-Based Intervention: The NOW Trial. Obesity. 2020 08;28(8):1419-27. PMID: 32935529. doi: https://dx.doi.org/10.1002/oby.22880.

272. Hosseininasab M, Jahangard-Rafsanjani Z, Mohagheghi A, Sarayani A, Rashidian A, Javadi M, et al. Self-monitoring of blood pressure for improving adherence to antihypertensive medicines and blood pressure control: a randomized controlled trial. Am J Hypertens. 2014 Nov;27(11):1339-45. PMID: 24771706. doi: https://dx.doi.org/10.1093/ajh/hpu062.

273. Howie-Esquivel J, Bibbins-Domingo K, Clark R, Evangelista L, Dracup K. A Culturally Appropriate Educational Intervention Can Improve Self-Care in Hispanic Patients With Heart Failure: A Pilot Randomized Controlled Trial. Cardiol Res. 2014 Aug;5(3-4):91-100. PMID: 28348704. doi: https://dx.doi.org/10.14740/cr346w.

274. Huang TT, Li YT, Wang CH. Individualized programme to promote self-care among older adults with asthma: randomized controlled trial. J Adv Nurs. 2009 Feb;65(2):348-58. PMID: 19040689. doi: https://dx.doi.org/10.1111/j.1365-2648.2008.04874.x.

275. Hudson B, Toop L, Mangin D, Pearson J. Risk communication methods in hip fracture prevention: a randomised trial in primary care. Br J Gen Pract. 2011 Aug;61(589):e469-76. PMID: 21801539. doi: https://dx.doi.org/10.3399/bjgp11X588439.

276. Hughes RCE, Rowan J, Williman J. Prediabetes in pregnancy, can early intervention improve outcomes? A feasibility study for a parallel randomised clinical trial. BMJ Open. 2018 03 03;8(3):e018493. PMID: 29502087. doi: https://dx.doi.org/10.1136/bmjopen-2017-018493.

277. Hunt CW, Sanderson BK, Ellison KJ. Support for diabetes using technology: a pilot study to improve self-management. Medsurg Nurs. 2014 Jul-Aug;23(4):231-7. PMID: 25318336.

278. Hunt JS, Siemienczuk J, Touchette D, Payne N. Impact of educational mailing on the blood pressure of primary care patients with mild hypertension. Journal of General Internal Medicine. 2004 Sep;19(9):925-30. PMID: 15333056.

279. Huseinovic E, Bertz F, Leu Agelii M, Hellebo Johansson E, Winkvist A, Brekke HK. Effectiveness of a weight loss intervention in postpartum women: results from a randomized controlled trial in primary health care. Am J Clin Nutr. 2016 Aug;104(2):362-70. PMID: 27413127. doi: https://dx.doi.org/10.3945/ajcn.116.135673.

280. Hutchison B, Birch S, Evans CE, Goldsmith LJ, Markham BA, Frank J, et al. Screening for hypercholesterolaemia in primary care: Randomised controlled trial of postal questionnaire appraising risk of coronary heart disease. Br Med J. 1998 Apr 18;316(7139):1208-13. PMID: 9552998. doi: 10.1136/bmj.316.7139.1208.

281. Iljaz R, Brodnik A, Zrimec T, Cukjati I. E-healthcare for Diabetes Mellitus Type 2 Patients - A Randomised Controlled Trial in Slovenia. Zdrav. 2017 Sep;56(3):150-7. PMID: 28713443. doi: https://dx.doi.org/10.1515/sjph-2017-0020.

282. Imai S, Kozai H, Naruse Y, Watanabe K, Fukui M, Hasegawa G, et al. Randomized controlled trial of two forms of self-management group education in Japanese people with impaired glucose tolerance. J. 2008 Sep;43(2):82-7. PMID: 18818757. doi: https://dx.doi.org/10.3164/jcbn.2008050.

283. Interrante MK, Segal H, Peshkin BN, Valdimarsdottir HB, Nusbaum R, Similuk M, et al. Randomized Noninferiority Trial of Telephone vs In-Person Genetic Counseling for Hereditary Breast and Ovarian Cancer: A 12-Month Follow-Up. JNCI cancer spectr. 2017 Sep;1(1):pkx002. PMID: 31304457. doi: https://dx.doi.org/10.1093/jncics/pkx002.

284. Ionov MV, Zhukova OV, Yudina YS, Avdonina NG, Emelyanov IV, Kurapeev DI, et al. Value-based approach to blood pressure telemonitoring and remote counseling in hypertensive patients. Blood Pressure. 2021;30(1):20-30. PMID: 32954832. doi: 10.1080/08037051.2020.1813015.

285. Irwig MS, Sood P, Ni D, Amass T, Khurana PS, Jayanthi VV, et al. A diabetes scorecard does not improve HbA(1c), blood pressure, lipids, aspirin usage, exercise and diabetes knowledge over 9 months: a randomized controlled trial. Diabet Med. 2012 Sep;29(9):1206-12. PMID: 22332914. doi: https://dx.doi.org/10.1111/j.1464-5491.2012.03610.x.

286. Isdale LB. DIABETES SELF-MANAGEMENT THROUGH EDUCATIONAL INTERVENTION [Ph.D.]. Ann Arbor: University of Illinois at Urbana-Champaign; 1983.

287. Ito H, Matsuo K, Wakai K, Saito T, Kumimoto H, Okuma K, et al. An intervention study of smoking cessation with feedback on genetic cancer susceptibility in Japan. Prev Med. 2006 Feb;42(2):102-8. PMID: 16325899.

288. Iwahori T, Ueshima H, Ohgami N, Yamashita H, Miyagawa N, Kondo K, et al. Effectiveness of a Self-monitoring Device for Urinary Sodium-to-Potassium Ratio on Dietary Improvement in Free-Living Adults: a Randomized Controlled Trial. J Epidemiol. 2018 Jan 05;28(1):41-7. PMID: 29093302. doi: https://dx.doi.org/10.2188/jea.JE20160144.

289. Izawa KP, Watanabe S, Omiya K, Hirano Y, Oka K, Osada N, et al. Effect of the self-monitoring approach on exercise maintenance during cardiac rehabilitation: a randomized, controlled trial. Am J Phys Med Rehabil. 2005 May;84(5):313-21. PMID: 15829777.

290. Izquierdo R, Lagua CT, Meyer S, Ploutz-Snyder RJ, Palmas W, Eimicke JP, et al. Telemedicine intervention effects on waist circumference and body mass index in the IDEATel project. Diabetes Technol Ther. 2010 Mar;12(3):213-20. PMID: 20151772. doi: https://dx.doi.org/10.1089/dia.2009.0102.

291. Jaen-Moreno MJ, Feu N, Del Pozo GI, Gomez C, Carrion L, Chauca GM, et al. Chronic obstructive pulmonary disease in severe mental illness: A timely diagnosis to advance the process of quitting smoking. Eur Psychiatry. 2021 Feb 26;64(1):e22. PMID: 33632347. doi: https://dx.doi.org/10.1192/j.eurpsy.2021.12.

292. Jahangard-Rafsanjani Z, Sarayani A, Nosrati M, Saadat N, Rashidian A, Hadjibabaie M, et al. Effect of a community pharmacist–delivered diabetes support program for patients receiving specialty medical care: A randomized controlled trial. The Diabetes Educator. 2015;41(1):127-35. PMID: 2015-03817-013. doi: 10.1177/0145721714559132.

293. Jaime PC, Bandoni DH, Sarno F. Impact of an education intervention using email for the prevention of weight gain among adult workers. Public Health Nutr. 2014 Jul;17(7):1620-7. PMID: 23962422. doi: https://dx.doi.org/10.1017/S1368980013001936.

294. Jamrozik K, Vessey M, Fowler G, Wald N, Parker G, Van Vunakis H. Controlled trial of three different antismoking interventions in general practice. Br Med J (Clin Res Ed). 1984 May 19;288(6429):1499-503. PMID: 6426618.

295. Janson SL, McGrath KW, Covington JK, Cheng SC, Boushey HA. Individualized asthma self-management improves medication adherence and markers of asthma control. J Allergy Clin Immunol. 2009 Apr;123(4):840-6. PMID: 19348923. doi: https://dx.doi.org/10.1016/j.jaci.2009.01.053.

296. Jeong JY, Jeon JH, Bae KH, Choi YK, Park KG, Kim JG, et al. Smart Care Based on Telemonitoring and Telemedicine for Type 2 Diabetes Care: Multi-Center Randomized Controlled Trial. Telemed J E Health. 2018 08;24(8):604-13. PMID: 29341843. doi: https://dx.doi.org/10.1089/tmj.2017.0203.

297. John LK, Loewenstein G, Troxel AB, Norton L, Fassbender JE, Volpp KG. Financial incentives for extended weight loss: A randomized, controlled trial. Journal of General Internal Medicine. 2011;26(6):621-6. PMID: 2011-10240-013. doi: 10.1007/s11606-010-1628-y.

298. Johnson KE, Alencar MK, Coakley KE, Swift DL, Cole NH, Mermier CM, et al. Telemedicine-Based Health Coaching Is Effective for Inducing Weight Loss and Improving Metabolic Markers. Telemed J E Health. 2019 02;25(2):85-92. PMID: 29847222. doi: https://dx.doi.org/10.1089/tmj.2018.0002.

299. Johnson NA, Kypri K, Saunders JB, Saitz R, Attia J, Latter J, et al. Effect of electronic screening and brief intervention on hazardous or harmful drinking among adults in the hospital outpatient setting: A randomized, double-blind, controlled trial. Drug Alcohol Depend. 2018 10 01;191:78-85. PMID: 30096637. doi: https://dx.doi.org/10.1016/j.drugalcdep.2018.06.030.

300. Johnson JA, Majumdar SR, Bowker SL, Toth EL, Edwards A. Self-monitoring in Type 2 diabetes: a randomized trial of reimbursement policy. Diabet Med. 2006 Nov;23(11):1247-51. PMID: 17054603.

301. Johnson AL, Taylor DW, Sackett DL, Dunnett CW, Shimizu AG. Self-recording of blood pressure in the management of hypertension. CAN MED ASSOC J. 1978 Nov 4;119(9):1034-9. PMID: 369673.

302. Jones H, Edwards L, Vallis TM, Ruggiero L, Rossi SR, Rossi JS, et al. Changes in diabetes self-care behaviors make a difference in glycemic control: the Diabetes Stages of Change (DiSC) study. Diabetes Care. 2003 Mar;26(3):732-7. PMID: 12610030.

303. Joseph J, Gotora T, Erlwanger AS, Mushavi A, Zizhou S, Masuka N, et al. Impact of Point-of-Care CD4 Testing on Retention in Care Among HIV-Positive Pregnant and Breastfeeding Women in the Context of Option B+ in Zimbabwe: A Cluster Randomized Controlled Trial. J Acquir Immune Defic Syndr. 2017 06 01;75 Suppl 2:S190-S7. PMID: 28498189. doi: https://dx.doi.org/10.1097/QAI.0000000000001341.

304. Jospe MR, e Bruin WE, Haszard JJ, Mann JI, Brunton M, Taylor RW. Teaching people to eat according to appetite - Does the method of glucose measurement matter? Appetite. 2020 08 01;151:104691. PMID: 32246953. doi: https://dx.doi.org/10.1016/j.appet.2020.104691.

305. Jospe MR, Roy M, Brown RC, Williams SM, Osborne HR, Meredith-Jones KA, et al. The Effect of Different Types of Monitoring Strategies on Weight Loss: A Randomized Controlled Trial. Obesity. 2017 09;25(9):1490-8. PMID: 28703448. doi: https://dx.doi.org/10.1002/oby.21898.

306. Kaczorowski J, Chambers LW, Dolovich L, Paterson JM, Karwalajtys T, Gierman T, et al. Improving cardiovascular health at population level: 39 community cluster randomised trial of Cardiovascular Health Awareness Program (CHAP). Bmj. 2011 Feb 07;342:d442. PMID: 21300712. doi: https://dx.doi.org/10.1136/bmj.d442.

307. Kaihara T, Eguchi K, Kario K. Home BP monitoring using a telemonitoring system is effective for controlling BP in a remote island in Japan. J Clin Hypertens (Greenwich). 2014 Nov;16(11):814-9. PMID: 25267008. doi: https://dx.doi.org/10.1111/jch.12421.

308. Kalluru R, Petrie KJ, Grey A, Nisa Z, Horne AM, Gamble GD, et al. Randomised trial assessing the impact of framing of fracture risk and osteoporosis treatment benefits in patients undergoing bone densitometry. BMJ Open. 2017 02 10;7(2):e013703. PMID: 28188155. doi: https://dx.doi.org/10.1136/bmjopen-2016-013703.

309. Kaminsky DA, Marcy T, Dorwaldt A, Pinckney R, DeSarno M, Solomon L, et al. Motivating smokers in the hospital pulmonary function laboratory to quit smoking by use of the lung age concept. Nicotine Tob Res. 2011 Nov;13(11):1161-6. PMID: 21551248. doi: https://dx.doi.org/10.1093/ntr/ntr096.

310. Kan K, Zhu W, Lu F, Shen Y, Gao F, Mo Y, et al. Contribution of Structured Self-Monitoring of Blood Glucose to the Glycemic Control and the Quality of Life in Both Insulin- and Noninsulin-Treated Patients with Poorly Controlled Diabetes. Diabetes Technology and Therapeutics. 2017;19(12):707-14. PMID: 29099626. doi: 10.1089/dia.2017.0275.

311. Kanke S, Kawai T, Takasawa N, Mashiyama Y, Ishii A, Kassai R. Interventions for body weight reduction in obese patients during short consultations: an open-label randomized controlled trial in the Japanese primary care setting. Asia Pac Fam Med. 2015;14(1):5. PMID: 26015773. doi: https://dx.doi.org/10.1186/s12930-015-0022-7.

312. Kanstrup H, Refsgaard J, Engberg M, Lassen JF, Larsen ML, Lauritzen T. Cholesterol reduction following health screening in general practice. Scand J Prim Health Care. 2002 Dec;20(4):219-23. PMID: 12564573.

313. Karhula T, Vuorinen AL, Raapysjarvi K, Pakanen M, Itkonen P, Tepponen M, et al. Telemonitoring and Mobile Phone-Based Health Coaching Among Finnish Diabetic and Heart Disease Patients: Randomized Controlled Trial. Journal of Medical Internet Research. 2015 Jun 17;17(6):e153. PMID: 26084979. doi: https://dx.doi.org/10.2196/jmir.4059.

314. Katterman SN, Butryn ML, Hood MM, Lowe MR. Daily weight monitoring as a method of weight gain prevention in healthy weight and overweight young adult women. Journal of Health Psychology. 2016 12;21(12):2955-65. PMID: 26069272.

315. Kauric-Klein Z. Improving blood pressure control in end stage renal disease through a supportive educative nursing intervention. Nephrol Nurs J. 2012 May-Jun;39(3):217-28. PMID: 22866361.

316. Kauric-Klein Z, Artinian N. Improving blood pressure control in hypertensive hemodialysis patients. Cannt J. 2007 Oct-Dec;17(4):24-8, 31-6; quiz 29-30, 7-8. PMID: 18271430.

317. Kaviani S, vanDellen M, Cooper JA. Daily Self-Weighing to Prevent Holiday-Associated Weight Gain in Adults. Obesity. 2019 06;27(6):908-16. PMID: 31119881. doi: https://dx.doi.org/10.1002/oby.22454.

318. Kempf K, Rohling M, Martin S, Schneider M. Telemedical coaching for weight loss in overweight employees: a three-armed randomised controlled trial. BMJ Open. 2019 04 11;9(4):e022242. PMID: 30975666. doi: https://dx.doi.org/10.1136/bmjopen-2018-022242.

319. Kempf K, Rohling M, Stichert M, Fischer G, Boschem E, Konner J, et al. Telemedical Coaching Improves Long-Term Weight Loss in Overweight Persons: A Randomized Controlled Trial. Int J Telemed Appl. 2018;2018:7530602. PMID: 30271433. doi: https://dx.doi.org/10.1155/2018/7530602.

320. Kempf K, Rohling M, Niedermeier K, Gartner B, Martin S. Individualized Meal Replacement Therapy Improves Clinically Relevant Long-Term Glycemic Control in Poorly Controlled Type 2 Diabetes Patients. Nutrients. 2018 Aug 04;10(8):04. PMID: 30081574. doi: https://dx.doi.org/10.3390/nu10081022.

321. Kempf K, Altpeter B, Berger J, Reus O, Fuchs M, Schneider M, et al. Efficacy of the Telemedical Lifestyle intervention Program TeLiPro in Advanced Stages of Type 2 Diabetes: A Randomized Controlled Trial. Diabetes Care. 2017 07;40(7):863-71. PMID: 28500214. doi: https://dx.doi.org/10.2337/dc17-0303.

322. Kempf K, Tankova T, Martin S. ROSSO-in-praxi-international: long-term effects of self-monitoring of blood glucose on glucometabolic control in patients with type 2 diabetes mellitus not treated with insulin. Diabetes Technol Ther. 2013 Jan;15(1):89-96. PMID: 23194054. doi: https://dx.doi.org/10.1089/dia.2012.0213.

323. Kerry SM, Markus HS, Khong TK, Cloud GC, Tulloch J, Coster D, et al. Home blood pressure monitoring with nurse-led telephone support among patients with hypertension and a history of stroke: a community-based randomized controlled trial. Cmaj. 2013 Jan 08;185(1):23-31. PMID: 23128283. doi: https://dx.doi.org/10.1503/cmaj.120832.

324. Kilic M, Karadag A. Developing and Evaluating a Mobile Foot Care Application for Persons With Diabetes Mellitus: A Randomized Pilot Study. Wound Manag Prev. 2020 10;66(10):29-40. PMID: 33048829.

325. Kim Y, Lee H, Seo JM. Integrated Diabetes Self-Management Program Using Smartphone Application: A Randomized Controlled Trial. West J Nurs Res. 2021 Mar 03:193945921994912. PMID: 33655794. doi: https://dx.doi.org/10.1177/0193945921994912.

326. Kim JW, Ryu B, Cho S, Heo E, Kim Y, Lee J, et al. Impact of Personal Health Records and Wearables on Health Outcomes and Patient Response: Three-Arm Randomized Controlled Trial. JMIR Mhealth Uhealth. 2019 01 04;7(1):e12070. PMID: 30609978. doi: https://dx.doi.org/10.2196/12070.

327. Kim JM, Lee HJ, Kim KO, Won JC, Ko KS, Rhee BD. Clinical evaluation of OneTouch Diabetes Management Software system in patients with type 2 diabetes mellitus. Diabetes and Metabolism Journal. 2016;40(2):129-39. doi: 10.4093/dmj.2016.40.2.129.

328. Kim HS, Sun C, Yang SJ, Sun L, Li F, Choi IY, et al. Randomized, Open-Label, Parallel Group Study to Evaluate the Effect of Internet-Based Glucose Management System on Subjects with Diabetes in China. Telemed J E Health. 2016 08;22(8):666-74. PMID: 26938489. doi: https://dx.doi.org/10.1089/tmj.2015.0170.

329. Kim YN, Shin DG, Park S, Lee CH. Randomized clinical trial to assess the effectiveness of remote patient monitoring and physician care in reducing office blood pressure. Hypertens Res. 2015 Jul;38(7):491-7. PMID: 25787041. doi: https://dx.doi.org/10.1038/hr.2015.32.

330. Kim JY, Oh S, Steinhubl S, Kim S, Bae WK, Han JS, et al. Effectiveness of 6 months of tailored text message reminders for obese male participants in a worksite weight loss program: randomized controlled trial. JMIR Mhealth Uhealth. 2015 Feb 03;3(1):e14. PMID: 25648325. doi: https://dx.doi.org/10.2196/mhealth.3949.

331. Kim MT, Han HR, Song HJ, Lee JE, Kim J, Ryu JP, et al. A community-based, culturally tailored behavioral intervention for Korean Americans with type 2 diabetes. Diabetes Educ. 2009 Nov-Dec;35(6):986-94. PMID: 19934458. doi: https://dx.doi.org/10.1177/0145721709345774.

332. Kim HS, Song MS. Technological intervention for obese patients with type 2 diabetes. Appl Nurs Res. 2008 May;21(2):84-9. PMID: 18457747. doi: https://dx.doi.org/10.1016/j.apnr.2007.01.007.

333. Kim HS, Jeong HS. A nurse short message service by cellular phone in type-2 diabetic patients for six months. J Clin Nurs. 2007 Jun;16(6):1082-7. PMID: 17518883.

334. Kim HS. A randomized controlled trial of a nurse short-message service by cellular phone for people with diabetes. Int J Nurs Stud. 2007 Jul;44(5):687-92. PMID: 16618486.

335. Kim HS. [Effects of Web-based diabetic education in obese diabetic patients]. Taehan Kanho Hakhoe Chi. 2005 Aug;35(5):924-30. PMID: 16208088.

336. Kim HS, Oh JA. Adherence to diabetes control recommendations: impact of nurse telephone calls. J Adv Nurs. 2003 Nov;44(3):256-61. PMID: 14641395.

337. Kirwan M, Vandelanotte C, Fenning A, Duncan MJ. Diabetes self-management smartphone application for adults with type 1 diabetes: randomized controlled trial. Journal of Medical Internet Research. 2013 Nov 13;15(11):e235. PMID: 24225149. doi: https://dx.doi.org/10.2196/jmir.2588.

338. Klarskov P, Bang LE, Schultz-Larsen P, Gregers Petersen H, Benee Olsen D, Berg RMG, et al. Intensive versus conventional blood pressure monitoring in a general practice population. The Blood Pressure Reduction in Danish General Practice trial: a randomized controlled parallel group trial. Fam Pract. 2018 07 23;35(4):433-9. PMID: 29351658. doi: https://dx.doi.org/10.1093/fampra/cmx106.

339. Kleefstra N, Hortensius J, Logtenberg SJ, Slingerland RJ, Groenier KH, Houweling ST, et al. Self-monitoring of blood glucose in tablet-treated type 2 diabetic patients (ZODIAC). Netherlands Journal of Medicine. 2010 Aug;68(1):311-6. PMID: 20739728.

340. Kleefstra N, Hortensius J, Logtenberg SJJ, Slingerl RJ, Groenier KH, Houweling ST, et al. Self-monitoring of blood glucose in tablet-treated type 2 diabetic patients (ZODIAC-17). Netherlands Journal of Medicine. 2010;68(7-8):311-6.

341. Knapen J, Sommerijns E, Vancampfort D, Sienaert P, Pieters G, Haake P, et al. State anxiety and subjective well-being responses to acute bouts of aerobic exercise in patients with depressive and anxiety disorders. British Journal of Sports Medicine. 2009 Oct;43(10):756-9. PMID: 19019899. doi: https://dx.doi.org/10.1136/bjsm.2008.052654.

342. Knapp PE, Showers KM, Phipps JC, Speckman JL, Sternthal E, Freund KM, et al. Self-monitoring of blood glucose with finger tip versus alternative site sampling: effect on glycemic control in insulin-using patients with type 2 diabetes. Diabetes Technol Ther. 2009 Apr;11(4):219-25. PMID: 19344196. doi: https://dx.doi.org/10.1089/dia.2008.0060.

343. Knight E, Stuckey MI, Petrella RJ. Health promotion through primary care: enhancing self-management with activity prescription and mHealth. Phys Sportsmed. 2014 Sep;42(3):90-9. PMID: 25295771. doi: https://dx.doi.org/10.3810/psm.2014.09.2080.

344. Knutson D. Exposure to a 'lung age' intervention, and likelihood to enroll in a tobacco cessation program among patients at military healthcare facilities: ProQuest Information & Learning; 2014.

345. Kocher E. Evaluating the Feasibility and Effectiveness of Self-Monitoring of Health – a Pilot Study among Samoan Women [M.P.H.]. Ann Arbor: Yale University; 2019.

346. Koelewijn-van Loon MS, van der Weijden T, van Steenkiste B, Ronda G, Winkens B, Severens JL, et al. Involving patients in cardiovascular risk management with nurse-led clinics: a cluster randomized controlled trial. Cmaj. 2009 Dec 08;181(12):E267-74. PMID: 19948811. doi: https://dx.doi.org/10.1503/cmaj.081591.

347. Komiya Y, Nakao H, Kuroda Y, Arizono K, Nakahara A, Katoh T. Application of aldehyde dehydrogenase 2 (ALDH2) genetic diagnosis in support of decreasing alcohol intake. Journal of Occupational Health. 2006 May;48(3):161-5. PMID: 16788276.

348. Kortke H, Frisch S, Zittermann A, Berthold HK, El-Arousy M, Gotting C, et al. [A telemetrically-guided program for weight reduction in overweight subjects (the SMART study)]. Dtsch Med Wochenschr. 2008 Jun;133(24):1297-303. PMID: 18528796. doi: https://dx.doi.org/10.1055/s-2008-1077256.

349. Kotsani K, Antonopoulou V, Kountouri A, Grammatiki M, Rapti E, Karras S, et al. The role of telenursing in the management of Diabetes Type 1: A randomized controlled trial. Int J Nurs Stud. 2018 Apr;80:29-35. PMID: 29353709. doi: https://dx.doi.org/10.1016/j.ijnurstu.2018.01.003.

350. Kotz D, Wesseling G, Huibers MJ, van Schayck OC. Efficacy of confronting smokers with airflow limitation for smoking cessation. Eur Respir J. 2009 Apr;33(4):754-62. PMID: 19129277. doi: https://dx.doi.org/10.1183/09031936.00116308.

351. Kovatchev BP, Mendosa P, Anderson S, Hawley JS, Ritterband LM, Gonder-Frederick L. Effect of automated bio-behavioral feedback on the control of type 1 diabetes. Diabetes Care. 2011 Feb;34(2):302-7. PMID: 21216860. doi: https://dx.doi.org/10.2337/dc10-1366.

352. Krass I, Armour CL, Mitchell B, Brillant M, Dienaar R, Hughes J, et al. The Pharmacy Diabetes Care Program: assessment of a community pharmacy diabetes service model in Australia. Diabet Med. 2007 Jun;24(6):677-83. PMID: 17523968.

353. Kreuter MW. Individually-tailored behavior change feedback and health risk appraisal [Ph.D.]. Ann Arbor: The University of North Carolina at Chapel Hill; 1993.

354. Krishnan N, Elf JL, Chon S, Golub JE. COach2Quit: A Pilot Randomized Controlled Trial of a Personal Carbon Monoxide Monitor for Smoking Cessation. Nicotine Tob Res. 2019 10 26;21(11):1573-7. PMID: 30169740. doi: https://dx.doi.org/10.1093/ntr/nty182.

355. Kristenson H, Osterling A, Nilsson JA, Lindgärde F. Prevention of alcohol-related deaths in middle-aged heavy drinkers. Alcohol Clin Exp Res. 2002 Apr;26(4):478-84. PMID: 11981123. doi: 10.1111/j.1530-0277.2002.tb02564.x 10.1111/j.1530-0277.2002.tb02564.x.

356. Kristenson H, Ohlin H, Hulten-Nosslin MB, Trell E, Hood B. Identification and intervention of heavy drinking in middle-aged men: results and follow-up of 24-60 months of long-term study with randomized controls. Alcohol Clin Exp Res. 1983;7(2):203-9. PMID: 6135365.

357. Ku EJ, Park JI, Jeon HJ, Oh T, Choi HJ. Clinical efficacy and plausibility of a smartphone-based integrated online real-time diabetes care system via glucose and diet data management: a pilot study. Intern Med J. 2020 Dec;50(12):1524-32. PMID: 31904890. doi: https://dx.doi.org/10.1111/imj.14738.

358. Kullo IJ, Jouni H, Austin EE, Brown SA, Kruisselbrink TM, Isseh IN, et al. Incorporating a Genetic Risk Score Into Coronary Heart Disease Risk Estimates: Effect on Low-Density Lipoprotein Cholesterol Levels (the MI-GENES Clinical Trial). Circulation. 2016 Mar 22;133(12):1181-8. PMID: 26915630. doi: https://dx.doi.org/10.1161/CIRCULATIONAHA.115.020109.

359. Kunath J, Gunther J, Rauh K, Hoffmann J, Stecher L, Rosenfeld E, et al. Effects of a lifestyle intervention during pregnancy to prevent excessive gestational weight gain in routine care - the cluster-randomised GeliS trial. BMC Medicine. 2019 01 14;17(1):5. PMID: 30636636. doi: https://dx.doi.org/10.1186/s12916-018-1235-z.

360. Kurscheid T, Redaélli M, Heinen A, Hahmann P, Behle K, Froböse I. App-gesteuerte Feedbackgeräte unterstützen Nachhaltigkeit einer Gewichtsreduktion: Multizentrische QUANT-Studie belegt zusätzliche Gewichtsreduktion und Lebensqualitätsverbesserung durch mehrere Feedbackgeräte im OPTIFAST®52-Programm = App-controlled feedback devices can support sustainability of weight loss Multicentre QUANT-study shows additional weight loss and gain of QoL via multiple feedback-devices in OPTIFAST®52-program. Zeitschrift für Psychosomatische Medizin und Psychotherapie. 2019;65(3):224-38. PMID: 2020-24400-001. doi: 10.13109/zptm.2019.65.3.224.

361. Kurscheid T, Redaelli M, Heinen A, Hahmann P, Behle K, Frobose I. [App-controlled feedback devices can support sustainability of weight loss. Multicentre QUANT-study shows additional weight loss and gain of QoL via multiple feedback-devices in OPTIFAST R52-program]. Z Psychosom Med Psychother. 2019 Sep;65(3):224-38. PMID: 31476994. doi: https://dx.doi.org/10.13109/zptm.2019.65.3.224.

362. Kurtzman GW, Day SC, Small DS, Lynch M, Zhu J, Wang W, et al. Social Incentives and Gamification to Promote Weight Loss: The LOSE IT Randomized, Controlled Trial. Journal of General Internal Medicine. 2018 10;33(10):1669-75. PMID: 30003481. doi: https://dx.doi.org/10.1007/s11606-018-4552-1.

363. Kwon HS, Cho JH, Kim HS, Song BR, Ko SH, Lee JM, et al. Establishment of blood glucose monitoring system using the internet. Diabetes Care. 2004 Feb;27(2):478-83. PMID: 14747232.

364. Laffel LM, Hsu WC, McGill JB, Meneghini L, Volkening LK. Continued use of an integrated meter with electronic logbook maintains improvements in glycemic control beyond a randomized, controlled trial. Diabetes Technol Ther. 2007 Jun;9(3):254-64. PMID: 17561796.

365. Lalla E, Cheng B, Kunzel C, Burkett S, Ferraro A, Lamster IB. Six-month outcomes in dental patients identified with hyperglycaemia: a randomized clinical trial. J Clin Periodontol. 2015 Mar;42(3):228-35. PMID: 25581313. doi: https://dx.doi.org/10.1111/jcpe.12358.

366. Lally P, Chipperfield A, Wardle J. Healthy habits: efficacy of simple advice on weight control based on a habit-formation model. International Journal of Obesity. 2008 Apr;32(4):700-7. PMID: 18071344.

367. Lam YY. The Effectiveness of eHealth Technology on Improving Self-Management of Adult Patients with Type 2 Diabetes (T2DM): Randomized Controlled Trial [Ph.D.]. Ann Arbor: The Chinese University of Hong Kong (Hong Kong); 2018.

368. Larkey LK, Gonzalez J. Storytelling for promoting colorectal cancer prevention and early detection among Latinos. Patient Educ Couns. 2007 Aug;67(3):272-8. PMID: 17524595.

369. LaRose JG, Neiberg RH, Evans EW, Tate DF, Espeland MA, Gorin AA, et al. Dietary outcomes within the study of novel approaches to weight gain prevention (SNAP) randomized controlled trial. Int. 2019 01 31;16(1):14. PMID: 30704533. doi: https://dx.doi.org/10.1186/s12966-019-0771-z.

370. Leahey TM, Thomas JG, LaRose JG, Wing RR. A randomized trial testing a contingency-based weight loss intervention involving social reinforcennent. Obesity. 2012;20(2):324-9. PMID: 2012-02943-010. doi: 10.1038/oby.2011.124.

371. Lear SA, Singer J, Banner-Lukaris D, Horvat D, Park JE, Bates J, et al. Improving access to cardiac rehabilitation using the internet: a randomized trial. Stud Health Technol Inform. 2015;209:58-66. PMID: 25980706.

372. Leavens ELS, Meier E, Tackett AP, Miller MB, Tahirkheli NN, Brett EI, et al. The impact of a brief cessation induction intervention for waterpipe tobacco smoking: A pilot randomized clinical trial. Addictive Behaviors. 2018 03;78:94-100. PMID: 29128712. doi: https://dx.doi.org/10.1016/j.addbeh.2017.10.023.

373. Lee DY, Yoo SH, Min KP, Park CY. Effect of Voluntary Participation on Mobile Health Care in Diabetes Management: Randomized Controlled Open-Label Trial. JMIR Mhealth Uhealth. 2020 09 18;8(9):e19153. PMID: 32945775. doi: https://dx.doi.org/10.2196/19153.

374. Lee SK, Shin DH, Kim YH, Lee KS. Effect of diabetes education through pattern management on self-care and self-efficacy in patients with type 2 diabetes. International Journal of Environmental Research and Public Health. 2019;16(18). PMID: 31505892. doi: 10.3390/ijerph16183323.

375. Lein DH. A pilot study for a computer-tailored program to increase calcium and vitamin D consumption in young women: Does adding heel quantitative ultrasound feedback change outcomes? : ProQuest Information & Learning; 2010.

376. Leite SA, Anderson RL, Kendall DM, Monk AM, Bergenstal RM. A1C predicts type 2 diabetes and impaired glucose tolerance in a population at risk: the community diabetes prevention project. Diabetol Metab Syndr. 2009 Sep 16;1(1):5. PMID: 19825200. doi: https://dx.doi.org/10.1186/1758-5996-1-5.

377. Leiva A, Aguiló A, Fajó-Pascual M, Moreno L, Martín MC, Garcia EM, et al. Efficacy of a brief multifactorial adherence-based intervention in reducing blood pressure: A randomized clinical trial. Patient Preference and Adherence. 2014;8:1683-90. doi: 10.2147/PPA.S66927.

378. Leskinen HM, Tringham M, Karjalainen H, Iso-Touru TK, Hietaranta-Luoma HL, Marnila PJ, et al. APOE Genotype Disclosure and Lifestyle Advice in a Randomized Intervention Study with Finnish Participants. J Nutr. 2021 01 04;151(1):85-97. PMID: 33188400. doi: https://dx.doi.org/10.1093/jn/nxaa316.

379. Levetan CS, Dawn KR, Murray JF, Popma JJ, Ratner RE, Robbins DC. Impact of computer-generated personalized goals on cholesterol lowering. Value Health. 2005 Nov-Dec;8(6):639-46. PMID: 16283864.

380. Levine JC, Burns E, Whittle J, Fleming R, Knudson P, Flax S, et al. Randomized trial of technology-assisted self-monitoring of blood glucose by low-income seniors: improved glycemic control in type 2 diabetes mellitus. J Behav Med. 2016 Dec;39(6):1001-8. PMID: 27368257. doi: https://dx.doi.org/10.1007/s10865-016-9763-5.

381. Levitsky DA, Garay J, Nausbaum M, Neighbors L, Dellavalle DM. Monitoring weight daily blocks the freshman weight gain: a model for combating the epidemic of obesity. International Journal of Obesity. 2006 Jun;30(6):1003-10. PMID: 16446748.

382. Li X, Li T, Chen J, Xie Y, An X, Lv Y, et al. A WeChat-Based Self-Management Intervention for Community Middle-Aged and Elderly Adults with Hypertension in Guangzhou, China: A Cluster-Randomized Controlled Trial. Int J Environ Res Public Health. 2019 10 23;16(21):23. PMID: 31652688. doi: https://dx.doi.org/10.3390/ijerph16214058.

383. Li CL, Wu YC, Kornelius E, Chen YJ, Lin YT, Hsiao HW, et al. Comparison of Different Models of Structured Self-Monitoring of Blood Glucose in Type 2 Diabetes. Diabetes Technol Ther. 2016 Mar;18(3):171-7. PMID: 26950418. doi: https://dx.doi.org/10.1089/dia.2015.0082.

384. Lillis J, Niemeier HM, Thomas JG, Unick J, Ross KM, Leahey TM, et al. A randomized trial of an acceptance-based behavioral intervention for weight loss in people with high internal disinhibition. Obesity. 2016 Dec;24(12):2509-14. PMID: 27804255. doi: https://dx.doi.org/10.1002/oby.21680.

385. Lim K, Chan SY, Lim SL, Tai BC, Tsai C, Wong SR, et al. A Smartphone App to Restore Optimal Weight (SPAROW) in Women With Recent Gestational Diabetes Mellitus: Randomized Controlled Trial. JMIR Mhealth Uhealth. 2021 03 16;9(3):e22147. PMID: 33724204. doi: https://dx.doi.org/10.2196/22147.

386. Lim S, Kang SM, Shin H, Lee HJ, Won Yoon J, Yu SH, et al. Improved glycemic control without hypoglycemia in elderly diabetic patients using the ubiquitous healthcare service, a new medical information system. Diabetes Care. 2011 Feb;34(2):308-13. PMID: 21270188. doi: https://dx.doi.org/10.2337/dc10-1447.

387. Lindberg I, Torbjornsen A, Soderberg S, Ribu L. Telemonitoring and Health Counseling for Self-Management Support of Patients With Type 2 Diabetes: A Randomized Controlled Trial. JMIR Diabetes. 2017 Jun 26;2(1):e10. PMID: 30291058. doi: https://dx.doi.org/10.2196/diabetes.6884.

388. Linde JA, Jeffery RW. Testing a brief self-directed behavioral weight control program. Behav Med. 2011 Apr;37(2):47-53. PMID: 21660772. doi: https://dx.doi.org/10.1080/08964289.2011.568992.

389. Lipkus IM, Schwartz-Bloom R, Kelley MJ, Pan W. A preliminary exploration of college smokers' reactions to nicotine dependence genetic susceptibility feedback. Nicotine Tob Res. 2015 Mar;17(3):337-43. PMID: 25173776. doi: https://dx.doi.org/10.1093/ntr/ntu155.

390. Lipkus IM, Prokhorov AV. The effects of providing lung age and respiratory symptoms feedback on community college smokers' perceived smoking-related health risks, worries and desire to quit. Addictive Behaviors. 2007 Mar;32(3):516-32. PMID: 16824688.

391. Littman AJ, Haselkorn JK, Arterburn DE, Boyko EJ. Pilot randomized trial of a telephone-delivered physical activity and weight management intervention for individuals with lower extremity amputation. Disabil Health J. 2019 01;12(1):43-50. PMID: 30115584. doi: https://dx.doi.org/10.1016/j.dhjo.2018.08.002.

392. Liu J, Wilcox S, Wingard E, Turner-McGrievy G, Hutto B, Burgis J. A Behavioral Lifestyle Intervention to Limit Gestational Weight Gain in Pregnant Women with Overweight and Obesity. Obesity. 2021 Apr;29(4):672-80. PMID: 33619910. doi: https://dx.doi.org/10.1002/oby.23119.

393. Liukkonen M, Nygard CH, Laukkanen R. A Cluster Randomized Controlled Trial on the Effects of Technology-aided Testing and Feedback on Physical Activity and Biological Age Among Employees in a Medium-sized Enterprise. Saf Health Work. 2017 Dec;8(4):393-7. PMID: 29276639. doi: https://dx.doi.org/10.1016/j.shaw.2017.03.003.

394. Livingstone KM, Celis-Morales C, Navas-Carretero S, San-Cristobal R, Macready AL, Fallaize R, et al. Effect of an Internet-based, personalized nutrition randomized trial on dietary changes associated with the Mediterranean diet: the Food4Me Study. Am J Clin Nutr. 2016 Aug;104(2):288-97. PMID: 27357094. doi: https://dx.doi.org/10.3945/ajcn.115.129049.

395. Logan AG, Irvine MJ, McIsaac WJ, Tisler A, Rossos PG, Easty A, et al. Effect of home blood pressure telemonitoring with self-care support on uncontrolled systolic hypertension in diabetics. Hypertension. 2012 Jul;60(1):51-7. PMID: 22615116. doi: https://dx.doi.org/10.1161/HYPERTENSIONAHA.111.188409.

396. Lopez-Gonzalez AA, Aguilo A, Frontera M, Bennasar-Veny M, Campos I, Vicente-Herrero T, et al. Effectiveness of the Heart Age tool for improving modifiable cardiovascular risk factors in a Southern European population: a randomized trial. Eur J Prev Cardiolog. 2015 Mar;22(3):389-96. PMID: 24491403. doi: https://dx.doi.org/10.1177/2047487313518479.

397. Lu J, Bu RF, Sun ZL, Lu QS, Jin H, Wang Y, et al. Comparable efficacy of self-monitoring of quantitative urine glucose with self-monitoring of blood glucose on glycaemic control in non-insulin-treated type 2 diabetes. Diabetes Res Clin Pract. 2011 Aug;93(2):179-86. PMID: 21570146. doi: https://dx.doi.org/10.1016/j.diabres.2011.04.012.

398. Luley C, Blaik A, Gotz A, Kicherer F, Kropf S, Isermann B, et al. Weight loss by telemonitoring of nutrition and physical activity in patients with metabolic syndrome for 1 year. J Am Coll Nutr. 2014;33(5):363-74. PMID: 25105874. doi: https://dx.doi.org/10.1080/07315724.2013.875437.

399. Ma J, Strub P, Xiao L, Lavori PW, Camargo CA, Wilson SR, et al. Behavioral weight loss and physical activity intervention in obese adults with asthma: A randomized trial. Annals of the American Thoracic Society. 2015;12(1):1-11. PMID: 25496399. doi: 10.1513/AnnalsATS.201406-271OC.

400. Mackenzie G, Ireland S, Moore S, Heinz I, Johnson R, Oczkowski W, et al. Tailored interventions to improve hypertension management after stroke or TIA--phase II (TIMS II). Can J Neurosci Nurs. 2013;35(1):27-34. PMID: 23687780.

401. Mackillop L, Hirst JE, Bartlett KJ, Birks JS, Clifton L, Farmer AJ, et al. Comparing the Efficacy of a Mobile Phone-Based Blood Glucose Management System With Standard Clinic Care in Women With Gestational Diabetes: Randomized Controlled Trial. JMIR Mhealth Uhealth. 2018 Mar 20;6(3):e71. PMID: 29559428. doi: https://dx.doi.org/10.2196/mhealth.9512.

402. Maddison R, Rawstorn JC, Stewart RAH, Benatar J, Whittaker R, Rolleston A, et al. Effects and costs of real-time cardiac telerehabilitation: randomised controlled non-inferiority trial. Heart. 2019 01;105(2):122-9. PMID: 30150328. doi: https://dx.doi.org/10.1136/heartjnl-2018-313189.

403. Madigan CD, Jolly K, Lewis AL, Aveyard P, Daley AJ. A randomised controlled trial of the effectiveness of self-weighing as a weight loss intervention. Int. 2014 Oct 10;11:125. PMID: 25301251. doi: https://dx.doi.org/10.1186/s12966-014-0125-9.

404. Mahler HIM. Effects of multiple viewings of an ultraviolet photo on sun protection behaviors. Public Health. 2018;160:33-40. PMID: 29729530. doi: 10.1016/j.puhe.2018.03.023.

405. Mahler HI, Kulik JA, Butler HA, Gerrard M, Gibbons FX. Social norms information enhances the efficacy of an appearance-based sun protection intervention. Soc Sci Med. 2008 Jul;67(2):321-9. PMID: 18448221. doi: https://dx.doi.org/10.1016/j.socscimed.2008.03.037.

406. Mahler HI, Kulik JA, Harrell J, Correa A, Gibbons FX, Gerrard M. Effects of UV photographs, photoaging information, and use of sunless tanning lotion on sun protection behaviors. Arch Dermatol. 2005 Mar;141(3):373-80. PMID: 15781679.

407. Mahoney MJ. Self reward and self monitoring techniques for weight control. Behavior Therapy. 1974;5(1):48-57.

408. Mahoney MJ, Moura NG, Wade TC. Relative efficacy of self-reward, self-punishment, and self-monitoring techniques for weight loss. J Consult Clin Psychol. 1973 Jun;40(3):404-7. PMID: 4708112.

409. Manfield DC. COMPUTER-ASSISTED WEIGHT-LOSS: A SUBLIMINAL AND BEHAVIORAL METHODOLOGY FOR MOTIVATED FEMALES [Ph.D.]. Ann Arbor: The University of Wisconsin - Madison; 1986.

410. Margolius D, Bodenheimer T, Bennett H, Wong J, Ngo V, Padilla G, et al. Health coaching to improve hypertension treatment in a low-income, minority population. Ann Fam Med. 2012 May-Jun;10(3):199-205. PMID: 22585883. doi: https://dx.doi.org/10.1370/afm.1369.

411. Marios T, N AS, Dalton S. The Effect of Tele-Monitoring on Exercise Training Adherence, Functional Capacity, Quality of Life and Glycemic Control in Patients With Type II Diabetes. J Sports Sci Med. 2012 Mar 01;11(1):51-6. PMID: 24137063.

412. Márquez Contreras E, Martel Claros N, Gil Guillén V, Martín De Pablos JL, De La Figuera Von Wichman M, Casado Martínez JJ, et al. Control of therapeutic inertia in the treatment of arterial hypertension by using different strategies. Atencion Primaria. 2009 Jun;41(6):315-23. PMID: 19482378. doi: 10.1016/j.aprim.2008.09.017.

413. Marquez-Contreras E, Martell-Claros N, Gil-Guillen V, e la Figuera-Von Wichmann M, Casado-Martinez JJ, Martin-de Pablos JL, et al. Efficacy of a home blood pressure monitoring programme on therapeutic compliance in hypertension: the EAPACUM-HTA study. J Hypertens. 2006 Jan;24(1):169-75. PMID: 16331115.

414. Marteau T, Senior V, Humphries SE, Bobrow M, Cranston T, Crook MA, et al. Psychological impact of genetic testing for familial hyperecholesterolemia within a previously aware population: A randomized controlled trial. American Journal of Medical Genetics. 2004;128 A(3):285-93.

415. Martin CK, Miller AC, Thomas DM, Champagne CM, Han H, Church T. Efficacy of SmartLoss, a smartphone-based weight loss intervention: results from a randomized controlled trial. Obesity. 2015 May;23(5):935-42. PMID: 25919921. doi: https://dx.doi.org/10.1002/oby.21063.

416. Martin-Lujan F, Basora-Gallisa J, Villalobos F, Martin-Vergara N, Aparicio-Llopis E, Pascual-Palacios I, et al. Effectiveness of a motivational intervention based on spirometry results to achieve smoking cessation in primary healthcare patients: randomised, parallel, controlled multicentre study. J Epidemiol Community Health. 2021 Apr 21;21:21. PMID: 33883199. doi: https://dx.doi.org/10.1136/jech-2020-216219.

417. Masaki K, Tateno H, Nomura A, Muto T, Suzuki S, Satake K, et al. A randomized controlled trial of a smoking cessation smartphone application with a carbon monoxide checker. npj digit. 2020;3:35. PMID: 32195370. doi: https://dx.doi.org/10.1038/s41746-020-0243-5.

418. Mason F, Farley A, Pallan M, Sitch A, Easter C, Daley AJ. Effectiveness of a brief behavioural intervention to prevent weight gain over the Christmas holiday period: randomised controlled trial. Bmj. 2018 Dec 10;363:k4867. PMID: 30530821. doi: https://dx.doi.org/10.1136/bmj.k4867.

419. Maurer SN. Choosing health: An online intervention to change eating/exercise (project choice): ProQuest Information & Learning; 2017.

420. McBride CM, Bepler G, Lipkus IM, Lyna P, Samsa G, Albright J, et al. Incorporating genetic susceptibility feedback into a smoking cessation program for African-American smokers with low income. Cancer Epidemiol Biomarkers Prev. 2002 Jun;11(6):521-8. PMID: 12050092.

421. McBride CM, Halabi S, Bepler G, Lyna P, McIntyre L, Lipkus I, et al. Maximizing the motivational impact of feedback of lung cancer susceptibility on smokers' desire to quit. J Health Commun. 2000 Jul-Sep;5(3):229-41. PMID: 11185023.

422. McCarthy EA, Walker SP, Ugoni A, Lappas M, Leong O, Shub A. Self-weighing and simple dietary advice for overweight and obese pregnant women to reduce obstetric complications without impact on quality of life: a randomised controlled trial. Bjog. 2016 May;123(6):965-73. PMID: 26875586. doi: https://dx.doi.org/10.1111/1471-0528.13919.

423. McClure JB, Swan GE, St. John J, Fauver R, Javitz HS, Bergen AW, et al. Pharmacogenetic smoking cessation intervention in a health care setting: A pilot feasibility study. Nicotine and Tobacco Research. 2013;15(2):518-26. PMID: 22949583. doi: 10.1093/ntr/nts173.

424. McClure JB, Ludman EJ, Grothaus L, Pabiniak C, Richards J. Impact of a brief motivational smoking cessation intervention the Get PHIT randomized controlled trial. American Journal of Preventive Medicine. 2009 Aug;37(2):116-23. PMID: 19524389. doi: https://dx.doi.org/10.1016/j.amepre.2009.03.018.

425. McConaha JL, Berdine HJ, Skomo ML, Laux RV, Higginbotham SK, O'Neil CK. Impact of the fracture risk assessment on patient and physician behavior in osteoporosis prevention. J Pharm Pract. 2014 Feb;27(1):25-30. PMID: 24108433. doi: https://dx.doi.org/10.1177/0897190013503970.

426. McDoniel SO, Wolskee P, Shen J. Treating obesity with a novel hand-held device, computer software program, and Internet technology in primary care: the SMART motivational trial. Patient Educ Couns. 2010 May;79(2):185-91. PMID: 19699049. doi: https://dx.doi.org/10.1016/j.pec.2009.07.034.

427. McKinstry B, Hanley J, Wild S, Pagliari C, Paterson M, Lewis S, et al. Telemonitoring based service redesign for the management of uncontrolled hypertension: multicentre randomised controlled trial. Bmj. 2013 May 24;346:f3030. PMID: 23709583. doi: https://dx.doi.org/10.1136/bmj.f3030.

428. McLean DL, McAlister FA, Johnson JA, King KM, Makowsky MJ, Jones CA, et al. A randomized trial of the effect of community pharmacist and nurse care on improving blood pressure management in patients with diabetes mellitus: study of cardiovascular risk intervention by pharmacists-hypertension (SCRIP-HTN). Arch Intern Med. 2008 Nov 24;168(21):2355-61. PMID: 19029501. doi: https://dx.doi.org/10.1001/archinte.168.21.2355.

429. McLeod KM. Osteoporosis care gap: ProQuest Information & Learning; 2021.

430. McMahon JM, Pouget ER, Tortu S, Volpe EM, Torres L, Rodriguez W. Couple-based HIV counseling and testing: a risk reduction intervention for US drug-involved women and their primary male partners. Prevention science. 2015;16(2):341‐51. PMID: CN-01131200. doi: 10.1007/s11121-014-0540-9.

431. McMahon GT, Gomes HE, Hohne SH, Hu TMJ, Levine BA, Conlin PR. Web-based care management in patients with poorly controlled diabetes. Diabetes Care. 2005 Jul;28(7):1624-9. PMID: 15983311. doi: 10.2337/diacare.28.7.1624.

432. McManus RJ, Little P, Stuart B, Morton K, Raftery J, Kelly J, et al. Home and Online Management and Evaluation of Blood Pressure (HOME BP) using a digital intervention in poorly controlled hypertension: randomised controlled trial. Bmj. 2021 01 19;372:m4858. PMID: 33468518. doi: https://dx.doi.org/10.1136/bmj.m4858.

433. McManus RJ, Mant J, Roalfe A, Oakes RA, Bryan S, Pattison HM, et al. Targets and self monitoring in hypertension: randomised controlled trial and cost effectiveness analysis. Bmj. 2005 Sep 03;331(7515):493. PMID: 16115830.

434. McNairy ML, Lamb MR, Gachuhi AB, Nuwagaba-Biribonwoha H, Burke S, Mazibuko S, et al. Effectiveness of a combination strategy for linkage and retention in adult HIV care in Swaziland: The Link4Health cluster randomized trial. PLoS Med. 2017 Nov;14(11):e1002420. PMID: 29112963. doi: https://dx.doi.org/10.1371/journal.pmed.1002420.

435. McRobbie H, Hajek P, Peerbux S, Kahan BC, Eldridge S, Trepel D, et al. Tackling obesity in areas of high social deprivation: clinical effectiveness and cost-effectiveness of a task-based weight management group programme - a randomised controlled trial and economic evaluation. Health Technol Assess. 2016 10;20(79):1-150. PMID: 27802843.

436. Medina KE. A behavioral lifestyle intervention for weight gain prevention in college students: ProQuest Information & Learning; 2018.

437. Mehos BM, Saseen JJ, MacLaughlin EJ. Effect of pharmacist intervention and initiation of home blood pressure monitoring in patients with uncontrolled hypertension. Pharmacotherapy. 2000 Nov;20(11):1384-9. PMID: 11079287.

438. Meisel SF, Beeken RJ, van Jaarsveld CH, Wardle J. Genetic susceptibility testing and readiness to control weight: Results from a randomized controlled trial. Obesity. 2015 Feb;23(2):305-12. PMID: 25522302. doi: https://dx.doi.org/10.1002/oby.20958.

439. Meisenhelder-Smith J. The effects of American Diabetes Association (ADA) diabetes self -management education and continuous glucose monitoring on diabetes health beliefs, behaviors and metabolic control [Ph.D.]. Ann Arbor: University of South Florida; 2006.

440. Meland E, Laerum E, Ulvik RJ. Salt restriction in hypertension--the effect of dietary advice and self monitoring of chloride concentration in urine. Scand J Clin Lab Invest. 1994 Aug;54(5):399-404. PMID: 7997845.

441. Mendelson M, Vivodtzev I, Tamisier R, Laplaud D, Dias-Domingos S, Baguet JP, et al. CPAP treatment supported by telemedicine does not improve blood pressure in high cardiovascular risk OSA patients: a randomized, controlled trial. Sleep. 2014 Nov 01;37(11):1863-70. PMID: 25364081. doi: https://dx.doi.org/10.5665/sleep.4186.

442. Meuleman Y, Hoekstra T, Dekker FW, Navis G, Vogt L, van der Boog PJM, et al. Sodium Restriction in Patients With CKD: A Randomized Controlled Trial of Self-management Support. Am J Kidney Dis. 2017 May;69(5):576-86. PMID: 27993433. doi: https://dx.doi.org/10.1053/j.ajkd.2016.08.042.

443. Miremberg H, Ben-Ari T, Betzer T, Raphaeli H, Gasnier R, Barda G, et al. The impact of a daily smartphone-based feedback system among women with gestational diabetes on compliance, glycemic control, satisfaction, and pregnancy outcome: a randomized controlled trial. Am J Obstet Gynecol. 2018 Apr;218(4):453.e1-.e7. PMID: 29425836. doi: https://dx.doi.org/10.1016/j.ajog.2018.01.044.

444. Mitchell NS, Manning BK, Staton EW, Emsermann CD, Dickinson LM, Pace WD. Outcomes of biomarker feedback on physical activity, eating habits, and emotional health: from the Americans in Motion-Healthy Intervention (AIM-HI) study. J Am Board Fam Med. 2014 Jan-Feb;27(1):61-9. PMID: 24390887. doi: https://dx.doi.org/10.3122/jabfm.2014.01.120347.

445. Mols RE, Jensen JM, Sand NP, Fuglesang C, Bagdat D, Vedsted P, et al. Visualization of Coronary Artery Calcification: Influence on Risk Modification. Am J Med. 2015 Sep;128(9):1023.e23-31. PMID: 25910787. doi: https://dx.doi.org/10.1016/j.amjmed.2015.03.033.

446. Monroe CM, Geraci M, Larsen CA, West DS. Feasibility and efficacy of a novel technology-based approach to harness social networks for weight loss: the NETworks pilot randomized controlled trial. Obes. 2019 Aug;5(4):354-65. PMID: 31452920. doi: https://dx.doi.org/10.1002/osp4.352.

447. Monroe VD. Testing a Smartphone Application Intervention to Improve Medication Adherence in African American Female Clinic Patients with Unstable High Blood Pressure: A Two-group Randomized Control Trial [M.S.]. Ann Arbor: Texas Woman's University; 2018.

448. Moore JO, Marshall MA, Judge DC, Moss FH, Gilroy SJ, Crocker JB, et al. Technology-supported apprenticeship in the management of hypertension: A randomized controlled trial. Journal of Clinical Outcomes Management. 2014;21(3):110-2.

449. Moreland EC, Volkening LK, Lawlor MT, Chalmers KA, Anderson BJ, Laffel LM. Use of a blood glucose monitoring manual to enhance monitoring adherence in adults with diabetes: a randomized controlled trial. Arch Intern Med. 2006 Mar 27;166(6):689-95. PMID: 16567610.

450. Morgan PJ, Callister R, Collins CE, Plotnikoff RC, Young MD, Berry N, et al. The SHED-IT community trial: a randomized controlled trial of internet- and paper-based weight loss programs tailored for overweight and obese men. Ann Behav Med. 2013 Apr;45(2):139-52. PMID: 23129021. doi: https://dx.doi.org/10.1007/s12160-012-9424-z.

451. Moriyasu T, Hosoda K, Tanaka-Mizuno S, Konda M, Ueshima K, Ida M, et al. Effects of monthly feedback of VFA measured by dual BIA method in Japanese patients with obesity: a randomized controlled study. Obes. 2017 12;3(4):407-16. PMID: 29259799. doi: https://dx.doi.org/10.1002/osp4.126.

452. Morrish NJ, Cohen DL, Hicks B, Keen H. A controlled study of the effect of computer-aided analysis of home blood glucose monitoring on blood glucose control. Diabet Med. 1989 Sep-Oct;6(7):591-4. PMID: 2527699.

453. Mosca L, Mochari H, Liao M, Christian AH, Edelman DJ, Aggarwal B, et al. A novel family-based intervention trial to improve heart health: FIT Heart: results of a randomized controlled trial. Circ Cardiovasc Qual Outcomes. 2008 Nov;1(2):98-106. PMID: 20031796. doi: https://dx.doi.org/10.1161/CIRCOUTCOMES.108.825786.

454. Muchmore DB, Springer J, Miller M. Self-monitoring of blood glucose in overweight type 2 diabetic patients. Acta Diabetol. 1994 Dec;31(4):215-9. PMID: 7888692.

455. Muhammad J, Jamial MM, Ishak A. Home Blood Pressure Monitoring Has Similar Effects on Office Blood Pressure and Medication Compliance as Usual Care. Korean J Fam Med. 2019 Sep;40(5):335-43. PMID: 30636386. doi: https://dx.doi.org/10.4082/kjfm.18.0026.

456. Muralidharan S, Ranjani H, Mohan Anjana R, Jena S, Tandon N, Gupta Y, et al. Engagement and Weight Loss: Results from the Mobile Health and Diabetes Trial. Diabetes Technol Ther. 2019 09;21(9):507-13. PMID: 31184922. doi: https://dx.doi.org/10.1089/dia.2019.0134.

457. Murphy HR, Rayman G, Lewis K, Kelly S, Johal B, Duffield K, et al. Effectiveness of continuous glucose monitoring in pregnant women with diabetes: randomised clinical trial. Bmj. 2008 Sep 25;337:a1680. PMID: 18818254. doi: https://dx.doi.org/10.1136/bmj.a1680.

458. Murray DM, Luepker RV, Pirie PL, Grimm RH, Jr B, E D, et al. Systematic risk factor screening and education: a community-wide approach to prevention of coronary heart disease. Prev Med. 1986 Nov;15(6):661-72. PMID: 3797397.

459. Nah EH, Chu J, Kim S, Cho S, Kwon E. Efficacy of lifestyle interventions in the reversion to normoglycemia in Korean prediabetics: One-year results from a randomised controlled trial. Prim Care Diabetes. 2019 06;13(3):212-20. PMID: 30583933. doi: https://dx.doi.org/10.1016/j.pcd.2018.11.017.

460. Napolitano MA, Whiteley JA, Mavredes M, Tjaden AH, Simmens S, Hayman LL, et al. Effect of tailoring on weight loss among young adults receiving digital interventions: an 18 month randomized controlled trial. Transl Behav Med. 2021 Apr 26;11(4):970-80. PMID: 33739422. doi: https://dx.doi.org/10.1093/tbm/ibab017.

461. Napolitano MA, Hayes S, Bennett GG, Ives AK, Foster GD. Using Facebook and text messaging to deliver a weight loss program to college students. Obesity. 2013 Jan;21(1):25-31. PMID: 23505165. doi: https://dx.doi.org/10.1002/oby.20232.

462. Neighbors C, Lee CM, Lewis MA, Fossos N, Walter T. Internet-based personalized feedback to reduce 21st-birthday drinking: a randomized controlled trial of an event-specific prevention intervention. J Consult Clin Psychol. 2009 Feb;77(1):51-63. PMID: 19170453. doi: https://dx.doi.org/10.1037/a0014386.

463. Nemanic T, Sarc I, Skrgat S, Flezar M, Cukjati I, Marc Malovrh M. Telemonitoring in asthma control: a randomized controlled trial. J Asthma. 2019 Jul;56(7):782-90. PMID: 30063840. doi: https://dx.doi.org/10.1080/02770903.2018.1493599.

464. Neupane D, McLachlan CS, Mishra SR, Olsen MH, Perry HB, Karki A, et al. Effectiveness of a lifestyle intervention led by female community health volunteers versus usual care in blood pressure reduction (COBIN): an open-label, cluster-randomised trial. Lancet Glob Health. 2018 01;6(1):e66-e73. PMID: 29241617. doi: https://dx.doi.org/10.1016/S2214-109X(17)30411-4.

465. Nezami BT, Ward DS, Lytle LA, Ennett ST, Tate DF. A mHealth randomized controlled trial to reduce sugar-sweetened beverage intake in preschool-aged children. Pediatr Obes. 2018 11;13(11):668-76. PMID: 29119719. doi: https://dx.doi.org/10.1111/ijpo.12258.

466. Nichols JAA, Grob P, Kite W, Williams P, e Lusignan S. Using a genetic/clinical risk score to stop smoking (GeTSS): randomised controlled trial. BMC Res Notes. 2017 Oct 23;10(1):507. PMID: 29061161. doi: https://dx.doi.org/10.1186/s13104-017-2831-2.

467. Nicolucci A, Cercone S, Chiriatti A, Muscas F, Gensini G. A Randomized Trial on Home Telemonitoring for the Management of Metabolic and Cardiovascular Risk in Patients with Type 2 Diabetes. Diabetes Technol Ther. 2015 Aug;17(8):563-70. PMID: 26154338. doi: https://dx.doi.org/10.1089/dia.2014.0355.

468. Nielsen DE, El-Sohemy A. Disclosure of genetic information and change in dietary intake: a randomized controlled trial. PLoS ONE. 2014;9(11):e112665. PMID: 25398084. doi: https://dx.doi.org/10.1371/journal.pone.0112665.

469. Nieuwkerk PT, Nierman MC, Vissers MN, Locadia M, Greggers-Peusch P, Knape LP, et al. Intervention to improve adherence to lipid-lowering medication and lipid-levels in patients with an increased cardiovascular risk. Am J Cardiol. 2012 Sep 01;110(5):666-72. PMID: 22621795. doi: https://dx.doi.org/10.1016/j.amjcard.2012.04.045.

470. Nilssen O. The Tromso Study: identification of and a controlled intervention on a population of early-stage risk drinkers. Prev Med. 1991 Jul;20(4):518-28. PMID: 1678518.

471. Nishi M, Kelleher V, Cronin M, Allen F. The effect of mobile personalised texting versus non-personalised texting on the caries risk of underprivileged adults: a randomised control trial. BMC Oral Health. 2019 03 12;19(1):44. PMID: 30866895. doi: https://dx.doi.org/10.1186/s12903-019-0729-1.

472. Nishimura A, Harashima SI, Fujita Y, Tanaka D, Wang Y, Liu Y, et al. Effects of structured testing versus routine testing of blood glucose in diabetes self-management: A randomized controlled trial. J Diabetes Complications. 2017 01;31(1):228-33. PMID: 27653670. doi: https://dx.doi.org/10.1016/j.jdiacomp.2016.08.019.

473. Nishimura A, Harashima S, Honda I, Shimizu Y, Harada N, Nagashima K, et al. Color record in self-monitoring of blood glucose improves glycemic control by better self-management. Diabetes Technol Ther. 2014 Jul;16(7):447-53. PMID: 24506479. doi: https://dx.doi.org/10.1089/dia.2013.0301.

474. Nolan RP, Upshur RE, Lynn H, Crichton T, Rukholm E, Stewart DE, et al. Therapeutic benefit of preventive telehealth counseling in the Community Outreach Heart Health and Risk Reduction Trial. Am J Cardiol. 2011 Mar 01;107(5):690-6. PMID: 21215382. doi: https://dx.doi.org/10.1016/j.amjcard.2010.10.050.

475. O'Donnell M, Alvarez-Iglesias A, McGuire BE, Dinneen SF. The impact of sharing personalised clinical information with people with type 2 diabetes prior to their consultation: A pilot randomised controlled trial. Patient Educ Couns. 2016 Apr;99(4):591-9. PMID: 26654869. doi: https://dx.doi.org/10.1016/j.pec.2015.11.014.

476. O'Kane MJ, Bunting B, Copeland M, Coates VE, group Es. Efficacy of self monitoring of blood glucose in patients with newly diagnosed type 2 diabetes (ESMON study): randomised controlled trial. Bmj. 2008 May 24;336(7654):1174-7. PMID: 18420662. doi: https://dx.doi.org/10.1136/bmj.39534.571644.BE.

477. O'Malley PG, Feuerstein IM, Taylor AJ. Impact of electron beam tomography, with or without case management, on motivation, behavioral change, and cardiovascular risk profile: a randomized controlled trial. Jama. 2003 May 07;289(17):2215-23. PMID: 12734132.

478. Ogbuokiri JE. Self-monitoring of blood pressures in hypertensive subjects and its effects on patient compliance. Drug Intell Clin Pharm. 1980 Jun;14(6):424-7. PMID: 10247885. doi: 10.1177/106002808001400606.

479. Ogedegbe G, Tobin JN, Fernandez S, Cassells A, Diaz-Gloster M, Khalida C, et al. Counseling African Americans to Control Hypertension: cluster-randomized clinical trial main effects. Circulation. 2014 May 20;129(20):2044-51. PMID: 24657991. doi: https://dx.doi.org/10.1161/CIRCULATIONAHA.113.006650.

480. Oh B, Cho B, Han MK, Choi H, Lee MN, Kang HC, et al. The Effectiveness of Mobile Phone-Based Care for Weight Control in Metabolic Syndrome Patients: Randomized Controlled Trial. JMIR Mhealth Uhealth. 2015 Aug 20;3(3):e83. PMID: 26293568. doi: https://dx.doi.org/10.2196/mhealth.4222.

481. Oh EG, Bang SY, Hyun SS, Kim SH, Chu SH, Jeon JY, et al. Effects of a 6-month lifestyle modification intervention on the cardiometabolic risk factors and health-related qualities of life in women with metabolic syndrome. Metabolism. 2010 Jul;59(7):1035-43. PMID: 20045151. doi: https://dx.doi.org/10.1016/j.metabol.2009.10.027.

482. Oh JA, Kim HS, Yoon KH, Choi ES. A telephone-delivered intervention to improve glycemic control in type 2 diabetic patients. Yonsei Med J. 2003 Feb;44(1):1-8. PMID: 12619168.

483. Ojedokun J, Keane S, O'Connor K. Lung age bio-feedback using a portable lung age meter with brief advice during routine consultations promote smoking cessation? Know2quit multicenter randomized control trial. J Gen Pract. 2013;1(123).

484. Ojji DB, Baldridge AS, Orji AI, Shedul LG, Ojji OI, Egenti NB, et al. Feasibility and effect of community health worker support and home monitoring for blood pressure control in Nigeria: a randomised pilot trial. Cardiovasc. 2020 May/Jun;31(3):213-5. PMID: 32627803. doi: https://dx.doi.org/10.5830/CVJA-2019-066.

485. Oliveria SA, Dusza SW, Phelan DL, Ostroff JS, Berwick M, Halpern AC. Patient adherence to skin self-examination. effect of nurse intervention with photographs. American Journal of Preventive Medicine. 2004 Feb;26(2):152-5. PMID: 14751328.

486. Olry de Labry Lima A, Bermudez Tamayo C, Pastor Moreno G, Bolivar Munoz J, Ruiz Perez I, Johri M, et al. Effectiveness of an intervention to improve diabetes self-management on clinical outcomes in patients with low educational level. Gac Sanit. 2017 Jan - Feb;31(1):40-7. PMID: 27477476. doi: https://dx.doi.org/10.1016/j.gaceta.2016.05.017.

487. Olson CM, Groth SW, Graham ML, Reschke JE, Strawderman MS, Fernandez ID. The effectiveness of an online intervention in preventing excessive gestational weight gain: the e-moms roc randomized controlled trial. BMC Pregnancy Childbirth. 2018 May 09;18(1):148. PMID: 29743026. doi: https://dx.doi.org/10.1186/s12884-018-1767-4.

488. Olson R, Wipfli B, Thompson SV, Elliot DL, Anger WK, Bodner T, et al. Weight Control Intervention for Truck Drivers: The SHIFT Randomized Controlled Trial, United States. American Journal of Public Health. 2016 Sep;106(9):1698-706. PMID: 27463067. doi: https://dx.doi.org/10.2105/AJPH.2016.303262.

489. Olson R, Thompson SV, Elliot DL, Hess JA, Rhoten KL, Parker KN, et al. Safety and Health Support for Home Care Workers: The COMPASS Randomized Controlled Trial. American Journal of Public Health. 2016 Oct;106(10):1823-32. PMID: 27552270. doi: https://dx.doi.org/10.2105/AJPH.2016.303327.

490. Olson ACF. Perimenopausal women's intended and actual behavioral response to bone health interventions [Ph.D.]. Ann Arbor: The University of Arizona; 2008.

491. Omar Y. A cognitive and behavioral intervention for smoking craving: Efficacy of heart rate variability biofeedback and cognitive reappraisal on craving, negative affect, cognitive performance, and psychophysiological measures in women who smoke cigarettes: ProQuest Information & Learning; 2019.

492. Or C, Tao D. A 3-Month Randomized Controlled Pilot Trial of a Patient-Centered, Computer-Based Self-Monitoring System for the Care of Type 2 Diabetes Mellitus and Hypertension. J Med Syst. 2016 Apr;40(4):81. PMID: 26802011. doi: https://dx.doi.org/10.1007/s10916-016-0437-1.

493. Orsama AL, Lahteenmaki J, Harno K, Kulju M, Wintergerst E, Schachner H, et al. Active assistance technology reduces glycosylated hemoglobin and weight in individuals with type 2 diabetes: results of a theory-based randomized trial. Diabetes Technol Ther. 2013 Aug;15(8):662-9. PMID: 23844570. doi: https://dx.doi.org/10.1089/dia.2013.0056.

494. Oshima Y, Matsuoka Y, Sakane N. Effect of weight-loss program using self-weighing twice a day and feedback in overweight and obese subject: a randomized controlled trial. Obes Res Clin Pract. 2013 Sep-Oct;7(5):e361-6. PMID: 24304478. doi: https://dx.doi.org/10.1016/j.orcp.2012.01.003.

495. Osmundson SS, Norton ME, El-Sayed YY, Carter S, Faig JC, Kitzmiller JL. Early Screening and Treatment of Women with Prediabetes: A Randomized Controlled Trial. American Journal of Perinatology. 2015;33(2):172-9. doi: 10.1055/s-0035-1563715.

496. Ostwald SK. Changing employees' dietary and exercise practices: an experimental study in a small company. J Occup Med. 1989 Feb;31(2):90-7. PMID: 2709166.

497. Pacanowski CR, Levitsky DA. Frequent Self-Weighing and Visual Feedback for Weight Loss in Overweight Adults. J Obes. 2015;2015:763680. PMID: 26064677. doi: https://dx.doi.org/10.1155/2015/763680.

498. Pacaud D, Kelley H, Downey AM, Chiasson M. Successful delivery of diabetes self-care education and follow-up through ehealth media. Canadian Journal of Diabetes. 2012;36(5):257-62. doi: 10.1016/j.jcjd.2012.08.006.

499. Park JJ, Kelly P, Carter BL, Burgess PP. Comprehensive pharmaceutical care in the chain setting. J Am Pharm Assoc (Wash). 1996 Jul;NS36(7):443-51. PMID: 8840744.

500. Parkes G, Greenhalgh T, Griffin M, Dent R. Effect on smoking quit rate of telling patients their lung age: the Step2quit randomised controlled trial. Bmj. 2008 Mar 15;336(7644):598-600. PMID: 18326503. doi: https://dx.doi.org/10.1136/bmj.39503.582396.25.

501. Parsons SN, Luzio SD, Harvey JN, Bain SC, Cheung WY, Watkins A, et al. Effect of structured self-monitoring of blood glucose, with and without additional TeleCare support, on overall glycaemic control in non-insulin treated Type 2 diabetes: the SMBG Study, a 12-month randomized controlled trial. Diabet Med. 2019 05;36(5):578-90. PMID: 30653704. doi: https://dx.doi.org/10.1111/dme.13899.

502. Patel MS, Small DS, Harrison JD, Hilbert V, Fortunato MP, Oon AL, et al. Effect of Behaviorally Designed Gamification With Social Incentives on Lifestyle Modification Among Adults With Uncontrolled Diabetes: A Randomized Clinical Trial. JAMA netw. 2021 May 03;4(5):e2110255. PMID: 34028550. doi: https://dx.doi.org/10.1001/jamanetworkopen.2021.10255.

503. Patel ML, Hopkins CM, Brooks TL, Bennett GG. Comparing Self-Monitoring Strategies for Weight Loss in a Smartphone App: Randomized Controlled Trial. JMIR Mhealth Uhealth. 2019 02 28;7(2):e12209. PMID: 30816851. doi: https://dx.doi.org/10.2196/12209.

504. Patten CA, Koller KR, Flanagan CA, Hiratsuka VY, Hughes CA, Wolfe AW, et al. Biomarker feedback intervention for smoking cessation among Alaska Native pregnant women: Randomized pilot study. Patient Educ Couns. 2019 03;102(3):528-35. PMID: 30391300. doi: https://dx.doi.org/10.1016/j.pec.2018.10.009.

505. Patten CA, Koller KR, Flanagan CA, Hiratsuka VY, Hughes CA, Wolfe AW, et al. Biomarker feedback intervention for smoking cessation among Alaska Native pregnant women: randomized pilot study. Patient education and counseling. 2018;(no pagination). PMID: CN-01668896. doi: 10.1016/j.pec.2018.10.009.

506. Pellegrini CA, Verba SD, Otto AD, Helsel DL, Davis KK, Jakicic JM. The comparison of a technology-based system and an in-person behavioral weight loss intervention. Obesity. 2012 Feb;20(2):356-63. PMID: 21311506. doi: https://dx.doi.org/10.1038/oby.2011.13.

507. Pelletier KR, Rodenburg A, Chikamoto Y, Vinther A, King AC, Farquhar JW. Managing job strain: A randomized controlled trial of an intervention conducted by mail and telephone. American Journal of Health Promotion. 1998;12(3):166-9. PMID: 10176090. doi: 10.4278/0890-1171-12.3.166.

508. Perera AI, Thomas MG, Moore JO, Faasse K, Petrie KJ. Effect of a Smartphone Application Incorporating Personalized Health-Related Imagery on Adherence to Antiretroviral Therapy: A Randomized Clinical Trial. AIDS Patient Care and STDs. 2014;28(11):579-86. PMID: 25290556. doi: 10.1089/apc.2014.0156.

509. Perez-Escamilla R, Damio G, Chhabra J, Fernandez ML, Segura-Perez S, Vega-Lopez S, et al. Impact of a community health workers-led structured program on blood glucose control among latinos with type 2 diabetes: the DIALBEST trial. Diabetes Care. 2015 Feb;38(2):197-205. PMID: 25125508. doi: https://dx.doi.org/10.2337/dc14-0327.

510. Persell SD, Peprah YA, Lipiszko D, Lee JY, Li JJ, Ciolino JD, et al. Effect of Home Blood Pressure Monitoring via a Smartphone Hypertension Coaching Application or Tracking Application on Adults With Uncontrolled Hypertension: A Randomized Clinical Trial. JAMA netw. 2020 03 02;3(3):e200255. PMID: 32119093. doi: https://dx.doi.org/10.1001/jamanetworkopen.2020.0255.

511. Petrella RJ, Stuckey MI, Shapiro S, Gill DP. Mobile health, exercise and metabolic risk: a randomized controlled trial. BMC Public Health. 2014 Oct 18;14:1082. PMID: 25326074. doi: https://dx.doi.org/10.1186/1471-2458-14-1082.

512. Pezzin LE, Feldman PH, Mongoven JM, McDonald MV, Gerber LM, Peng TR. Improving blood pressure control: results of home-based post-acute care interventions. Journal of General Internal Medicine. 2011 Mar;26(3):280-6. PMID: 20945114. doi: https://dx.doi.org/10.1007/s11606-010-1525-4.

513. Phelan S, Phipps MG, Abrams B, Darroch F, Schaffner A, Wing RR. Randomized trial of a behavioral intervention to prevent excessive gestational weight gain: the Fit for Delivery Study. Am J Clin Nutr. 2011 Apr;93(4):772-9. PMID: 21310836. doi: https://dx.doi.org/10.3945/ajcn.110.005306.

514. Piatt GA, Rodgers EA, Xue L, Zgibor JC. Integration and Utilization of Peer Leaders for Diabetes Self-Management Support: Results From Project SEED (Support, Education, and Evaluation in Diabetes). Diabetes Educ. 2018 08;44(4):373-82. PMID: 29806788. doi: https://dx.doi.org/10.1177/0145721718777855.

515. Piette JD, Datwani H, Gaudioso S, Foster SM, Westphal J, Perry W, et al. Hypertension management using mobile technology and home blood pressure monitoring: results of a randomized trial in two low/middle-income countries. Telemed J E Health. 2012 Oct;18(8):613-20. PMID: 23061642. doi: https://dx.doi.org/10.1089/tmj.2011.0271.

516. Piette JD, Weinberger M, Kraemer FB, McPhee SJ. Impact of automated calls with nurse follow-up on diabetes treatment outcomes in a Department of Veterans Affairs Health Care System: a randomized controlled trial. Diabetes Care. 2001 Feb;24(2):202-8. PMID: 11213866.

517. Piette JD, Weinberger M, McPhee SJ, Mah CA, Kraemer FB, Crapo LM. Do automated calls with nurse follow-up improve self-care and glycemic control among vulnerable patients with diabetes? Am J Med. 2000 Jan;108(1):20-7. PMID: 11059437.

518. Pollak KI, Alexander SC, Bennett G, Lyna P, Coffman CJ, Bilheimer A, et al. Weight-related SMS texts promoting appropriate pregnancy weight gain: a pilot study. Patient Educ Couns. 2014 Nov;97(2):256-60. PMID: 25153313. doi: https://dx.doi.org/10.1016/j.pec.2014.07.030.

519. Polley BA, Wing RR, Sims CJ. Randomized controlled trial to prevent excessive weight gain in pregnant women. Int J Obes Relat Metab Disord. 2002 Nov;26(11):1494-502. PMID: 12439652.

520. Polonsky WH, Fisher L, Schikman CH, Hinnen DA, Parkin CG, Jelsovsky Z, et al. Structured self-monitoring of blood glucose significantly reduces A1C levels in poorly controlled, noninsulin-treated type 2 diabetes: results from the Structured Testing Program study. Diabetes Care. 2011 Feb;34(2):262-7. PMID: 21270183. doi: https://dx.doi.org/10.2337/dc10-1732.

521. Polzien KM. The effectiveness of a computer and Internet-based system in a short-term behavioral weight loss intervention [Ph.D.]. Ann Arbor: University of Pittsburgh; 2005.

522. Ponzo S, Morelli D, Kawadler JM, Hemmings NR, Bird G, Plans D. Efficacy of the Digital Therapeutic Mobile App BioBase to Reduce Stress and Improve Mental Well-Being Among University Students: Randomized Controlled Trial. JMIR Mhealth Uhealth. 2020 04 06;8(4):e17767. PMID: 31926063. doi: https://dx.doi.org/10.2196/17767.

523. Powers BJ, Danus S, Grubber JM, Olsen MK, Oddone EZ, Bosworth HB. The effectiveness of personalized coronary heart disease and stroke risk communication. Am Heart J. 2011 Apr;161(4):673-80. PMID: 21473965. doi: https://dx.doi.org/10.1016/j.ahj.2010.12.021.

524. Price HC. Personalised cardiovascular disease risk information as a motivator of behaviour change in individuals at high cardiovascular disease risk [Ph.D.]. Ann Arbor: University of Oxford (United Kingdom); 2010.

525. Prokhorov AV, Yost T, Mullin-Jones M, e Moor C, Ford KH, Marani S, et al. "Look at your health": outcomes associated with a computer-assisted smoking cessation counseling intervention for community college students. Addictive Behaviors. 2008 Jun;33(6):757-71. PMID: 18280668. doi: https://dx.doi.org/10.1016/j.addbeh.2007.12.005.

526. Proper KI, van der Beek AJ, Hildebrandt VH, Twisk JW, van Mechelen W. Short term effect of feedback on fitness and health measurements on self reported appraisal of the stage of change. British Journal of Sports Medicine. 2003 Dec;37(6):529-34. PMID: 14665593.

527. Quinlivan JA, Lam LT, Fisher J. A randomised trial of a four-step multidisciplinary approach to the antenatal care of obese pregnant women. Aust N Z J Obstet Gynaecol. 2011 Apr;51(2):141-6. PMID: 21466516. doi: https://dx.doi.org/10.1111/j.1479-828X.2010.01268.x.

528. Quinn CC, Shardell MD, Terrin ML, Barr EA, Ballew SH, Gruber-Baldini AL. Cluster-randomized trial of a mobile phone personalized behavioral intervention for blood glucose control. Diabetes Care. 2011 Sep;34(9):1934-42. PMID: 21788632. doi: https://dx.doi.org/10.2337/dc11-0366.

529. Quinn CC, Clough SS, Minor JM, Lender D, Okafor MC, Gruber-Baldini A. WellDoc mobile diabetes management randomized controlled trial: change in clinical and behavioral outcomes and patient and physician satisfaction. Diabetes Technol Ther. 2008 Jun;10(3):160-8. PMID: 18473689. doi: https://dx.doi.org/10.1089/dia.2008.0283.

530. Rand CS, Stitzer ML, Bigelow GE, Mead AM. The effects of contingent payment and frequent workplace monitoring on smoking abstinence. Addictive Behaviors. 1989;14(2):121-8. PMID: 2728951.

531. Rash CJ, Petry NM, Alessi SM. A randomized trial of contingency management for smoking cessation in the homeless. Psychol Addict Behav. 2018 03;32(2):141-8. PMID: 29461070. doi: https://dx.doi.org/10.1037/adb0000350.

532. Rasjo Wraak G, Tornkvist L, Hasselstrom J, Wandell PE, Josefsson K. Nurse-led empowerment strategies for patients with hypertension: a questionnaire survey. Int Nurs Rev. 2015 Jun;62(2):187-95. PMID: 25664779. doi: https://dx.doi.org/10.1111/inr.12174.

533. Rat C, Quereux G, Riviere C, Clouet S, Senand R, Volteau C, et al. Targeted melanoma prevention intervention: a cluster randomized controlled trial. Ann Fam Med. 2014 Jan-Feb;12(1):21-8. PMID: 24445100. doi: https://dx.doi.org/10.1370/afm.1600.

534. Rauh K, Gabriel E, Kerschbaum E, Schuster T, von Kries R, Amann-Gassner U, et al. Safety and efficacy of a lifestyle intervention for pregnant women to prevent excessive maternal weight gain: a cluster-randomized controlled trial. BMC Pregnancy Childbirth. 2013 Jul 16;13:151. PMID: 23865624. doi: https://dx.doi.org/10.1186/1471-2393-13-151.

535. Reading AE, Campbell S, Cox DN, Sledmere CM. Health beliefs and health care behaviour in pregnancy. Psychological Medicine. 1982;12(2):379-83. PMID: 1983-01684-001. doi: 10.1017/S0033291700046717.

536. Redfern J, Coorey G, Mulley J, Scaria A, Neubeck L, Hafiz N, et al. A digital health intervention for cardiovascular disease management in primary care (CONNECT) randomized controlled trial. npj digit. 2020;3:117. PMID: 32964140. doi: https://dx.doi.org/10.1038/s41746-020-00325-z.

537. Redman LM, Gilmore LA, Breaux J, Thomas DM, Elkind-Hirsch K, Stewart T, et al. Effectiveness of SmartMoms, a Novel eHealth Intervention for Management of Gestational Weight Gain: Randomized Controlled Pilot Trial. JMIR Mhealth Uhealth. 2017 Sep 13;5(9):e133. PMID: 28903892. doi: https://dx.doi.org/10.2196/mhealth.8228.

538. Redman S, Sanson-Fisher R, Kreft S, Fleming J, Dickinson J. Is the Australian National Heart Foundation programme effective in reducing cholesterol levels among general practice patients? Health Promotion International. 1995;10(4):293-303.

539. Rees G, Lamoureux EL, Nicolaou TE, Hodgson LA, Weinman J, Speight J. Feedback of personal retinal images appears to have a motivational impact in people with non-proliferative diabetic retinopathy and suboptimal HbA1c: findings of a pilot study. Diabet Med. 2013 Sep;30(9):1122-5. PMID: 23601012. doi: https://dx.doi.org/10.1111/dme.12192.

540. Reid RD, McDonnell LA, Riley DL, Mark AE, Mosca L, Beaton L, et al. Effect of an intervention to improve the cardiovascular health of family members of patients with coronary artery disease: a randomized trial. Cmaj. 2014 Jan 07;186(1):23-30. PMID: 24246588. doi: https://dx.doi.org/10.1503/cmaj.130550.

541. Reynolds KA. Impact of visual feedback on exercise intensity and motivation [M.S.]. Ann Arbor: Illinois State University; 2016.

542. Reynolds KD, Gillum JL, Hyman DJ, Byers T, Moore SA, Paradis G, et al. Comparing two strategies to modify dietary behavior and serum cholesterol. J Cardiovasc Risk. 1997 Feb;4(1):1-5. PMID: 9215513.

543. Richard E, Moll van Charante EP, Hoevenaar-Blom MP, Coley N, Barbera M, van der Groep A, et al. Healthy ageing through internet counselling in the elderly (HATICE): a multinational, randomised controlled trial. Lancet Digit Health. 2019 12;1(8):e424-e34. PMID: 33323224. doi: https://dx.doi.org/10.1016/S2589-7500(19)30153-0.

544. Richmond RL. EVALUATION OF THE USE BY GENERAL PRACTITIONERS OF AN INTERVENTION PROGRAMME FOR SMOKING CESSATION [Ph.D.]. Ann Arbor: University of New South Wales (Australia); 1984.

545. Ridner SH, Shih YC, Doersam JK, Rhoten BA, Schultze BS, Dietrich MS. A pilot randomized trial evaluating lymphedema self-measurement with bioelectrical impedance, self-care adherence, and health outcomes. Lymphat. 2014 Dec;12(4):258-66. PMID: 25412401. doi: https://dx.doi.org/10.1089/lrb.2014.0017.

546. Rief W, Conradt M, Dierk JM, Rauh E, Schlumberger P, Hinney A, et al. Is information on genetic determinants of obesity helpful or harmful for obese people?--A randomized clinical trial. Journal of General Internal Medicine. 2007 Nov;22(11):1553-9. PMID: 17879121.

547. Rinfret S, Lussier MT, Peirce A, Duhamel F, Cossette S, Lalonde L, et al. The impact of a multidisciplinary information technology-supported program on blood pressure control in primary care. Circ Cardiovasc Qual Outcomes. 2009 May;2(3):170-7. PMID: 20031834. doi: https://dx.doi.org/10.1161/CIRCOUTCOMES.108.823765.

548. Ring-Dimitriou S, von Duvillard SP, Stadlmann M, Kinnunen H, Drachta O, Muller E, et al. Changes in physical fitness in moderately fit adults with and without the use of exercise telemetry monitors. Eur J Appl Physiol. 2008 Mar;102(5):505-13. PMID: 18026864.

549. Risser NL, Belcher DW. Adding spirometry, carbon monoxide, and pulmonary symptom results to smoking cessation counseling: a randomized trial. Journal of General Internal Medicine. 1990 Jan-Feb;5(1):16-22. PMID: 2405112.

550. Robertson I, Phillips A, Mant D, Thorogood M, Fowler G, Fuller A, et al. Motivational effect of cholesterol measurement in general practice health checks. Br J Gen Pract. 1992 Nov;42(364):469-72. PMID: 1472394.

551. Roblin DW, Zelman D, Plummer S, Robinson BE, Lou Y, Edmonds SW, et al. Evaluation of a "Just-in-Time" Nurse Consultation on Bone Health: A Pilot Randomized Controlled Trial. Perm. 2017;21:16-112. PMID: 28746019. doi: https://dx.doi.org/10.7812/TPP/16-112.

552. Rodondi N, Collet TH, Nanchen D, Locatelli I, Depairon M, Aujesky D, et al. Impact of carotid plaque screening on smoking cessation and other cardiovascular risk factors: a randomized controlled trial. Arch Intern Med. 2012 Feb 27;172(4):344-52. PMID: 22269590. doi: https://dx.doi.org/10.1001/archinternmed.2011.1326.

553. Rodríguez-Idígoras MI, Sepúlveda-Muñoz J, Sánchez-Garrido-Escudero R, Martínez-González JL, Escolar-Castelló JL, Paniagua-Gómez IM, et al. Telemedicine influence on the follow-up of type 2 diabetes patients. Diabetes Technology and Therapeutics. 2009 Jul;11(7):431-7. PMID: 19580356. doi: 10.1089/dia.2008.0114.

554. Rogers RJ. The comparison of a technology-based system and in-person behavioral weight loss intervention in the severely obese: ProQuest Information & Learning; 2014.

555. Rogers MAM, Small D, Buchan DA, Butch CA, Stewart CM, Krenzer BE, et al. Home monitoring service improves mean arterial pressure in patients with essential hypertension: A randomized, controlled trial. Annals of Internal Medicine. 2001 Jun 5;134(11):1024-32. PMID: 11388815. doi: 10.7326/0003-4819-134-11-200106050-00008.

556. Roke K, Walton K, Klingel SL, Harnett A, Subedi S, Haines J, et al. Evaluating changes in omega-3 fatty acid intake after receiving personal FADS1 genetic information: A randomized nutrigenetic intervention. Nutrients. 2017 Mar 6;9(3). PMID: 28272299. doi: 10.3390/nu9030240.

557. Roke K. Exploration of the perceived and actual benefits of omega-3 fatty acids and the impact of FADS1 and FADS2 genetic information on dietary intake and blood levels of EPA and DHA. Appl Physiol Nutr Metab. 2017 Mar;42(3):333. PMID: 28186827. doi: https://dx.doi.org/10.1139/apnm-2016-0700.

558. Rolnick SJ, Kopher R, Jackson J, Fischer LR, Compo R. What is the impact of osteoporosis education and bone mineral density testing for postmenopausal women in a managed care setting? Menopause. 2001;8(2):141-8. PMID: 11256875.

559. Ronnberg AK, Ostlund I, Fadl H, Gottvall T, Nilsson K. Intervention during pregnancy to reduce excessive gestational weight gain-a randomised controlled trial. Bjog. 2015 Mar;122(4):537-44. PMID: 25367823. doi: https://dx.doi.org/10.1111/1471-0528.13131.

560. Rosal MC, Ockene IS, Restrepo A, White MJ, Borg A, Olendzki B, et al. Randomized trial of a literacy-sensitive, culturally tailored diabetes self-management intervention for low-income latinos: latinos en control. Diabetes Care. 2011 Apr;34(4):838-44. PMID: 21378213. doi: https://dx.doi.org/10.2337/dc10-1981.

561. Rosario MBD, Lovell NH, Fildes J, Holgate K, Yu J, Ferry C, et al. Evaluation of an mHealth-Based Adjunct to Outpatient Cardiac Rehabilitation. IEEE j. 2018 11;22(6):1938-48. PMID: 29990228. doi: https://dx.doi.org/10.1109/JBHI.2017.2782209.

562. Rosenberg SD, Goldberg RW, Dixon LB, Wolford GL, Slade EP, Himelhoch S, et al. Assessing the STIRR model of best practices for blood-borne infections of clients with severe mental illness. Psychiatr Serv. 2010 Sep;61(9):885-91. PMID: 20810586. doi: https://dx.doi.org/10.1176/ps.2010.61.9.885.

563. Ross KM, Wing RR. Impact of newer self-monitoring technology and brief phone-based intervention on weight loss: A randomized pilot study. Obesity. 2016 08;24(8):1653-9. PMID: 27367614. doi: https://dx.doi.org/10.1002/oby.21536.

564. Roux C, Giraudeau B, Rouanet S, Dubourg G, Perrodeau E, Ravaud P. Monitoring of bone turnover markers does not improve persistence with ibandronate treatment. Joint Bone Spine. 2012 Jul;79(4):389-92. PMID: 21703900. doi: https://dx.doi.org/10.1016/j.jbspin.2011.05.001.

565. Rouyard T, Leal J, Baskerville R, Velardo C, Salvi D, Gray A. Nudging people with Type 2 diabetes towards better self-management through personalized risk communication: A pilot randomized controlled trial in primary care. Endocrinol. 2018 Jul;1(3):e00022. PMID: 30815556. doi: https://dx.doi.org/10.1002/edm2.22.

566. Rozanski A, Gransar H, Shaw LJ, Kim J, Miranda-Peats L, Wong ND, et al. Impact of coronary artery calcium scanning on coronary risk factors and downstream testing the EISNER (Early Identification of Subclinical Atherosclerosis by Noninvasive Imaging Research) prospective randomized trial. J Am Coll Cardiol. 2011 Apr 12;57(15):1622-32. PMID: 21439754. doi: https://dx.doi.org/10.1016/j.jacc.2011.01.019.

567. Rumchev K, Brown H, Wheeler A, Pereira G, Spickett J. Behavioral interventions to reduce nickel exposure in a nickel processing plant. J Occup Environ Hyg. 2017 10;14(10):823-30. PMID: 28641054. doi: https://dx.doi.org/10.1080/15459624.2017.1335402.

568. Ruppar TM. Randomized pilot study of a behavioral feedback intervention to improve medication adherence in older adults with hypertension. J Cardiovasc Nurs. 2010 Nov-Dec;25(6):470-9. PMID: 20856132. doi: https://dx.doi.org/10.1097/JCN.0b013e3181d5f9c5.

569. Sakakibara BM, Lear SA, Barr SI, Goldsmith CH, Schneeberg A, Silverberg ND, et al. Telehealth coaching to improve self-management for secondary prevention after stroke: A randomized controlled trial of Stroke Coach. Int j. 2021 May 27:17474930211017699. PMID: 33949270. doi: https://dx.doi.org/10.1177/17474930211017699.

570. Sakane N, Kotani K, Takahashi K, Sano Y, Tsuzaki K, Okazaki K, et al. Effects of telephone-delivered lifestyle support on the development of diabetes in participants at high risk of type 2 diabetes: J-DOIT1, a pragmatic cluster randomised trial. BMJ Open. 2015 Aug 19;5(8):e007316. PMID: 26289448. doi: https://dx.doi.org/10.1136/bmjopen-2014-007316.

571. Samaan Z, Schulze KM, Middleton C, Irvine J, Joseph P, Mente A, et al. South Asian Heart Risk Assessment (SAHARA): Randomized Controlled Trial Design and Pilot Study. JMIR Res Protoc. 2013 Aug 20;2(2):e33. PMID: 23965279. doi: https://dx.doi.org/10.2196/resprot.2621.

572. Sandborg J, Soderstrom E, Henriksson P, Bendtsen M, Henstrom M, Leppanen MH, et al. Effectiveness of a Smartphone App to Promote Healthy Weight Gain, Diet, and Physical Activity During Pregnancy (HealthyMoms): Randomized Controlled Trial. JMIR Mhealth Uhealth. 2021 03 11;9(3):e26091. PMID: 33704075. doi: https://dx.doi.org/10.2196/26091.

573. Sanderson SC, Humphries SE, Hubbart C, Hughes E, Jarvis MJ, Wardle J. Psychological and behavioural impact of genetic testing smokers for lung cancer risk: A phase II exploratory trial. Journal of Health Psychology. 2008 May;13(4):481-94. PMID: 18420756. doi: 10.1177/1359105308088519.

574. Sandquist E. Use of contingent monetary reinforcement and feedback to reduce smoking for adults diagnosed with schizophrenia [M.A.]. Ann Arbor: University of the Pacific; 2005.

575. Sarfo FS, Treiber F, Gebregziabher M, Adamu S, Nichols M, Singh A, et al. Phone-based intervention for blood pressure control among Ghanaian stroke survivors: A pilot randomized controlled trial. Int j. 2019 08;14(6):630-8. PMID: 30465630. doi: https://dx.doi.org/10.1177/1747493018816423.

576. Saslow LR, Moskowitz JT, Mason AE, Daubenmier J, Liestenfeltz B, Missel AL, et al. Intervention Enhancement Strategies Among Adults With Type 2 Diabetes in a Very Low-Carbohydrate Web-Based Program: Evaluating the Impact With a Randomized Trial. JMIR Diabetes. 2020 Sep 09;5(3):e15835. PMID: 32902391. doi: https://dx.doi.org/10.2196/15835.

577. Sason A, Adelson M, Herzman-Harari S, Peles E. Knowledge about nutrition, eating habits and weight reduction intervention among methadone maintenance treatment patients. Journal of Substance Abuse Treatment. 2018;86:52-9. PMID: 29415851. doi: 10.1016/j.jsat.2017.12.008.

578. Schmid-Mohler G, Zala P, Graf N, Witschi P, Mueller TF, Peter Wuthrich R, et al. Comparison of a Behavioral Versus an Educational Weight Management Intervention After Renal Transplantation: A Randomized Controlled Trial. Transplant Direct. 2019 Dec;5(12):e507. PMID: 32095502. doi: https://dx.doi.org/10.1097/TXD.0000000000000936.

579. Schopp LH, Clark MJ, Lamberson WR, Uhr DJ, Minor MA. A randomized controlled trial to evaluate outcomes of a workplace self-management intervention and an intensive monitoring intervention. Health education research. 2017;32(3):219‐32. PMID: CN-01628017. doi: 10.1093/her/cyx042.

580. Schroy PC, Iii E, Karen P, Ellen G, Julie T R, Patricia A L, et al. The impact of a novel computer-based decision aid on shared decision making for colorectal cancer screening: A randomized trial. Medical Decision Making. 2011;31(1):93-107. PMID: 2011-09417-009. doi: 10.1177/0272989X10369007.

581. Schuz N, Schuz B, Eid M. When risk communication backfires: randomized controlled trial on self-affirmation and reactance to personalized risk feedback in high-risk individuals. Health Psychology. 2013 May;32(5):561-70. PMID: 23646839. doi: https://dx.doi.org/10.1037/a0029887.

582. Schwartz MD, Peshkin BN, Isaacs C, Willey S, Valdimarsdottir HB, Nusbaum R, et al. Randomized trial of proactive rapid genetic counseling versus usual care for newly diagnosed breast cancer patients. Breast Cancer Res Treat. 2018 Aug;170(3):517-24. PMID: 29611029. doi: https://dx.doi.org/10.1007/s10549-018-4773-3.

583. Schwedes U, Siebolds M, Mertes G, Group SS. Meal-related structured self-monitoring of blood glucose: effect on diabetes control in non-insulin-treated type 2 diabetic patients. Diabetes Care. 2002 Nov;25(11):1928-32. PMID: 12401734.

584. Segnan N, Ponti A, Battista RN, Senore C, Rosso S, Shapiro SH, et al. A randomized trial of smoking cessation interventions in general practice in Italy. Cancer Causes Control. 1991 Jul;2(4):239-46. PMID: 1873454.

585. Seijo-Bestilleiro R, Seoane-Pillado T, Pertega-Diaz S, Gonzalez-Martin C, Valdes-Canedo F, Balboa-Barreiro V, et al. Randomized clinical trial to determine the effectiveness of CO-oximetry and anti-smoking brief advice in a cohort of kidney transplant patients who smoke. Int J Med Sci. 2020;17(17):2673-84. PMID: 33162795. doi: https://dx.doi.org/10.7150/ijms.49401.

586. Sejourne C, Parot-Schinckel E, Rouquette A, Pare F, Delcroix M, Fanello S. [Impact of exhaled CO measurement. A randomised study among 578 smoking patients in general practice]. Rev Mal Respir. 2010 Mar;27(3):213-8. PMID: 20359612. doi: https://dx.doi.org/10.1016/j.rmr.2010.01.006.

587. Seligman HK, Smith M, Rosenmoss S, Marshall MB, Waxman E. Comprehensive Diabetes Self-Management Support From Food Banks: A Randomized Controlled Trial. American Journal of Public Health. 2018 09;108(9):1227-34. PMID: 30024798. doi: https://dx.doi.org/10.2105/AJPH.2018.304528.

588. Sen AP, Sewell TB, Riley EB, Stearman B, Bellamy SL, Hu MF, et al. Financial incentives for home-based health monitoring: a randomized controlled trial. Journal of General Internal Medicine. 2014 May;29(5):770-7. PMID: 24522623. doi: https://dx.doi.org/10.1007/s11606-014-2778-0.

589. Sequeira PA, Montoya L, Ruelas V, Xing D, Chen V, Beck R, et al. Continuous glucose monitoring pilot in low-income type 1 diabetes patients. Diabetes Technol Ther. 2013 Oct;15(10):855-8. PMID: 23865840. doi: https://dx.doi.org/10.1089/dia.2013.0072.

590. Sevick MA, Korytkowski M, Stone RA, Piraino B, Ren D, Sereika S, et al. Biophysiologic outcomes of the Enhancing Adherence in Type 2 Diabetes (ENHANCE) trial. J Acad Nutr Diet. 2012 Aug;112(8):1147-57. PMID: 22818724. doi: https://dx.doi.org/10.1016/j.jand.2012.05.008.

591. Shahab L, West R, McNeill A. A randomized, controlled trial of adding expired carbon monoxide feedback to brief stop smoking advice: evaluation of cognitive and behavioral effects. Health Psychology. 2011 Jan;30(1):49-57. PMID: 21299294. doi: https://dx.doi.org/10.1037/a0021821.

592. Shakudo M, Takegami M, Shibata A, Kuzumaki M, Higashi T, Hayashino Y, et al. Effect of feedback in promoting adherence to an exercise programme: a randomized controlled trial. J Eval Clin Pract. 2011 Feb;17(1):7-11. PMID: 20738470. doi: https://dx.doi.org/10.1111/j.1365-2753.2009.01342.x.

593. Sherwood NE, Crain AL, Martinson BC, Anderson CP, Hayes MG, Anderson JD, et al. Enhancing long-term weight loss maintenance: 2 year results from the Keep It Off randomized controlled trial. Prev Med. 2013 Mar;56(3-4):171-7. PMID: 23276775. doi: https://dx.doi.org/10.1016/j.ypmed.2012.12.014.

594. Shuger SL, Barry VW, Sui X, McClain A, Hand GA, Wilcox S, et al. Electronic feedback in a diet- and physical activity-based lifestyle intervention for weight loss: a randomized controlled trial. Int. 2011 May 18;8:41. PMID: 21592351. doi: https://dx.doi.org/10.1186/1479-5868-8-41.

595. Sidhu MS, Daley A, Jolly K. Evaluation of a text supported weight maintenance programme 'Lighten Up Plus' following a weight reduction programme: randomised controlled trial. Int. 2016 Feb 12;13:19. PMID: 26867588. doi: https://dx.doi.org/10.1186/s12966-016-0346-1.

596. Silarova B, Sharp S, Usher-Smith JA, Lucas J, Payne RA, Shefer G, et al. Effect of communicating phenotypic and genetic risk of coronary heart disease alongside web-based lifestyle advice: the INFORM Randomised Controlled Trial. Heart. 2019 07;105(13):982-9. PMID: 30928969. doi: https://dx.doi.org/10.1136/heartjnl-2018-314211.

597. Silverman SL, Nasser K, Nattrass S, Drinkwater B. Impact of bone turnover markers and/or educational information on persistence to oral bisphosphonate therapy: a community setting-based trial. Osteoporos Int. 2012 Mar;23(3):1069-74. PMID: 21755401. doi: https://dx.doi.org/10.1007/s00198-011-1721-z.

598. Silverman SL, Greenwald M, Klein RA, Drinkwater BL. Effect of bone density information on decisions about hormone replacement therapy: a randomized trial. Obstet Gynecol. 1997 Mar;89(3):321-5. PMID: 9052578.

599. Sippel JM, Osborne ML, Bjornson W, Goldberg B, Buist AS. Smoking cessation in primary care clinics. Journal of General Internal Medicine. 1999 Nov;14(11):670-6. PMID: 10571715.

600. Skaaby T, Jorgensen T, Linneberg A. A randomized general population study of the effects of repeated health checks on incident diabetes. Endocrine. 2018 04;60(1):122-8. PMID: 29417371. doi: https://dx.doi.org/10.1007/s12020-018-1542-3.

601. Skobel E, Knackstedt C, Martinez-Romero A, Salvi D, Vera-Munoz C, Napp A, et al. Internet-based training of coronary artery patients: the Heart Cycle Trial. Heart Vessels. 2017 Apr;32(4):408-18. PMID: 27730298. doi: https://dx.doi.org/10.1007/s00380-016-0897-8.

602. Skrovseth SO, Arsand E, Godtliebsen F, Joakimsen RM. Data-Driven Personalized Feedback to Patients with Type 1 Diabetes: A Randomized Trial. Diabetes Technol Ther. 2015 Jul;17(7):482-9. PMID: 25751133. doi: https://dx.doi.org/10.1089/dia.2014.0276.

603. Smit AK, Espinoza D, Newson AJ, Morton RL, Fenton G, Freeman L, et al. A Pilot Randomized Controlled Trial of the Feasibility, Acceptability, and Impact of Giving Information on Personalized Genomic Risk of Melanoma to the Public. Cancer Epidemiol Biomarkers Prev. 2017 02;26(2):212-21. PMID: 27702805. doi: https://dx.doi.org/10.1158/1055-9965.EPI-16-0395.

604. Smith EN, Santoro E, Moraveji N, Susi M, Crum AJ. Integrating wearables in stress management interventions: Promising evidence from a randomized trial. International Journal of Stress Management. 2020;27(2):172-82. PMID: 2019-36704-001. doi: 10.1037/str0000137 10.1037/str0000137.supp (Supplemental).

605. Smith WA, Ekdahl SS, Henley CE. Use of Health Hazard Appraisal in counseling for reduction of risk factors. J Am Osteopath Assoc. 1985 Dec;85(12):809-14. PMID: 3908417.

606. Sniehotta FF, Evans EH, Sainsbury K, Adamson A, Batterham A, Becker F, et al. Behavioural intervention for weight loss maintenance versus standard weight advice in adults with obesity: A randomised controlled trial in the UK (NULevel Trial). PLoS Med. 2019 05;16(5):e1002793. PMID: 31063507. doi: https://dx.doi.org/10.1371/journal.pmed.1002793.

607. Snoek FJ, van der Ven NC, Twisk JW, Hogenelst MH, Tromp-Wever AM, van der Ploeg HM, et al. Cognitive behavioural therapy (CBT) compared with blood glucose awareness training (BGAT) in poorly controlled Type 1 diabetic patients: long-term effects on HbA moderated by depression. A randomized controlled trial. Diabet Med. 2008 Nov;25(11):1337-42. PMID: 19046225. doi: https://dx.doi.org/10.1111/j.1464-5491.2008.02595.x.

608. Soureti A, Murray P, Cobain M, van Mechelen W, Hurling R. Web-based risk communication and planning in an obese population: exploratory study. Journal of Medical Internet Research. 2011 Nov 24;13(4):e100. PMID: 22126827. doi: https://dx.doi.org/10.2196/jmir.1579.

609. Souza WK, Jardim PC, Brito LP, Araujo FA, Sousa AL. Self measurement of blood pressure for control of blood pressure levels and adherence to treatment. Arq Bras Cardiol. 2012 Feb;98(2):167-74. PMID: 22249412.

610. Sparks JA, Iversen MD, Yu Z, Triedman NA, Prado MG, Miller Kroouze R, et al. Disclosure of Personalized Rheumatoid Arthritis Risk Using Genetics, Biomarkers, and Lifestyle Factors to Motivate Health Behavior Improvements: A Randomized Controlled Trial. Arthritis Care Res (Hoboken). 2018 06;70(6):823-33. PMID: 29024454. doi: https://dx.doi.org/10.1002/acr.23411.

611. Spring B, Pellegrini CA, Pfammatter A, Duncan JM, Pictor A, McFadden HG, et al. Effects of an abbreviated obesity intervention supported by mobile technology: The ENGAGED randomized clinical trial. Obesity. 2017 07;25(7):1191-8. PMID: 28494136. doi: https://dx.doi.org/10.1002/oby.21842.

612. Spruill TM, Feltheimer SD, Harlapur M, Schwartz JE, Ogedegbe G, Park Y, et al. Are there consequences of labeling patients with prehypertension? An experimental study of effects on blood pressure and quality of life. J Psychosom Res. 2013 May;74(5):433-8. PMID: 23597332. doi: https://dx.doi.org/10.1016/j.jpsychores.2013.01.009.

613. Stahl SM, Kelley CR, Neill PJ, Grim CE, Mamlin J. Effects of home blood pressure measurement on long-term BP control. American Journal of Public Health. 1984 Jul;74(7):704-9. PMID: 6742256.

614. Steinberg G, Scott A, Honcz J, Spettell C, Pradhan S. Reducing Metabolic Syndrome Risk Using a Personalized Wellness Program. J Occup Environ Med. 2015 Dec;57(12):1269-74. PMID: 26474447. doi: https://dx.doi.org/10.1097/JOM.0000000000000582.

615. Steinberg DM, Tate DF, Bennett GG, Ennett S, Samuel-Hodge C, Ward DS. The efficacy of a daily self-weighing weight loss intervention using smart scales and e-mail. Obesity. 2013 Sep;21(9):1789-97. PMID: 23512320. doi: https://dx.doi.org/10.1002/oby.20396.

616. Stewart K, George J, Mc Namara KP, Jackson SL, Peterson GM, Bereznicki LR, et al. A multifaceted pharmacist intervention to improve antihypertensive adherence: a cluster-randomized, controlled trial (HAPPy trial). J Clin Pharm Ther. 2014 Oct;39(5):527-34. PMID: 24943987. doi: https://dx.doi.org/10.1111/jcpt.12185.

617. Still CH, Margevicius S, Harwell C, Huang MC, Martin L, Dang PB, et al. A Community and Technology-Based Approach for Hypertension Self-Management (COACHMAN) to Improve Blood Pressure Control in African Americans: Results from a Pilot Study. Patient Prefer Adherence. 2020;14:2301-13. PMID: 33262580. doi: https://dx.doi.org/10.2147/PPA.S283086.

618. Stock ML, Gerrard M, Gibbons FX, Dykstra JL, Weng CY, Mahler HI, et al. Sun protection intervention for highway workers: long-term efficacy of UV photography and skin cancer information on men's protective cognitions and behavior. Ann Behav Med. 2009 Dec;38(3):225-36. PMID: 20049658. doi: https://dx.doi.org/10.1007/s12160-009-9151-2.

619. Stotts AL, Groff JY, Velasquez MM, Benjamin-Garner R, Green C, Carbonari JP, et al. Ultrasound feedback and motivational interviewing targeting smoking cessation in the second and third trimesters of pregnancy. Nicotine Tob Res. 2009 Aug;11(8):961-8. PMID: 19553282. doi: https://dx.doi.org/10.1093/ntr/ntp095.

620. Stotts AL, Potts GF, Ingersoll G, George MR, Martin LE. Preliminary feasibility and efficacy of a brief motivational intervention with psychophysiological feedback for cocaine abuse. Subst Abus. 2006 Dec;27(4):9-20. PMID: 17347121.

621. Strimas R, Dionne MM. Differential effects of self-weighing in restrained and unrestrained eaters. Personality and Individual Differences. 2010;49(8):1011-4. PMID: 2010-19620-030. doi: 10.1016/j.paid.2010.07.035.

622. Strychar IM, Champagne F, Ghadirian P, Bonin A, Jenicek M, Lasater TM. Impact of receiving blood cholesterol test results on dietary change. American Journal of Preventive Medicine. 1998 Feb;14(2):103-10. PMID: 9631161.

623. Sun YQ, Jia YP, Lv JY, Ma GJ. The clinical effects of a new management mode for hypertensive patients: a randomized controlled trial. Cardiovasc. 2020 Dec;10(6):1805-15. PMID: 33381425. doi: https://dx.doi.org/10.21037/cdt-20-589.

624. Sun M, Tan J, Liu S. Correlation between blood glucose level and self-management level in patients with type II diabetes mellitus. International Journal of Clinical and Experimental Medicine. 2020;13(2):664-72.

625. Sun J, Wang Y, Chen X, Chen Y, Feng Y, Zhang X, et al. An integrated intervention program to control diabetes in overweight Chinese women and men with type 2 diabetes. Asia Pac J Clin Nutr. 2008;17(3):514-24. PMID: 18818173.

626. Suokas AE. Brief intervention of heavy drinking in primary health care: Hameenlinna study [M.D.]. Ann Arbor: Helsingin Yliopisto (Finland); 1992.

627. Suriyawongpaisal P, Tansirisithikul R, Sakulpipat T, Charoensuk P, Aekplakorn W. A Participatory Randomized Controlled Trial in Knowledge Translation (KT) to Promote the Adoption of Self-Monitoring of Blood Glucose for Type 2 Diabetes Mellitus Patients in An Urban District of Thailand. J Med Assoc Thai. 2016 Feb;99(2):125-32. PMID: 27249891.

628. Suvorova LA, Petrov AV, Strongin LG. The effects of structured self-monitoring of blood glucose on therapeutic effectiveness and adherence in patients with type 2 diabetes mellitus initiating insulin treatment. Diabetes Mellitus. 2013 (4):52-7. doi: 10.14341/DM2013452-57.

629. Svendsen K, Jacobs DR, Morch-Reiersen LT, Garstad KW, Henriksen HB, Telle-Hansen VH, et al. Evaluating the use of the heart age tool in community pharmacies: a 4-week cluster-randomized controlled trial. Eur J Public Health. 2020 12 11;30(6):1139-45. PMID: 32206810. doi: https://dx.doi.org/10.1093/eurpub/ckaa048.

630. Svetkey LP, Batch BC, Lin PH, Intille SS, Corsino L, Tyson CC, et al. Cell phone intervention for you (CITY): A randomized, controlled trial of behavioral weight loss intervention for young adults using mobile technology. Obesity. 2015 Nov;23(11):2133-41. PMID: 26530929. doi: https://dx.doi.org/10.1002/oby.21226.

631. Szpiler JA. AN EXAMINATION OF THE SUCCORANCE VARIABLE AS A PREDICTOR OF DIETARY SUCCESS IN A SELF-MANAGED PROGRAM OF WEIGHT REDUCTION [Educat.D.]. Ann Arbor: University of Massachusetts Amherst; 1980.

632. Takagi H, Morio Y, Ishiwata T, Shimada K, Kume A, Miura K, et al. Effect of telling patients their "spirometric-lung-age" on smoking cessation in Japanese smokers. Journal of Thoracic Disease. 2017 Dec;9(12):5052-60. PMID: 29312710. doi: 10.21037/jtd.2017.11.06.

633. Takata Y, Ou O, Nishida H, Sakagami K. Impact of self-monitoring of blood glucose on the lifestyles of subjects with fasting hyperglycemia: A randomized controlled trial. Journal of Occupational Health. 2002;44(1):28-33. doi: 10.1539/joh.44.28.

634. Tan MY, Magarey JM, Chee SS, Lee LF, Tan MH. A brief structured education programme enhances self-care practices and improves glycaemic control in Malaysians with poorly controlled diabetes. Health Education Research. 2011 Oct;26(5):896-907. PMID: 21715653. doi: https://dx.doi.org/10.1093/her/cyr047.

635. Tanaka M, Adachi Y, Adachi K, Sato C. Effects of a non-face-to-face behavioral weight-control program among Japanese overweight males: a randomized controlled trial. Int J Behav Med. 2010 Mar;17(1):17-24. PMID: 19685190. doi: https://dx.doi.org/10.1007/s12529-009-9057-1.

636. Tanenbaum RL. PSYCHOLOGICAL AND PHYSICAL EFFECTS OF OCCUPATIONAL HEALTH AND STRESS MANAGEMENT INTERVENTIONS [Ph.D.]. Ann Arbor: Virginia Commonwealth University; 1984.

637. Tang TS, Funnell MM, Sinco B, Spencer MS, Heisler M. Peer-Led, Empowerment-Based Approach to Self-Management Efforts in Diabetes (PLEASED): A Randomized Controlled Trial in an African American Community. Ann Fam Med. 2015 Aug;13 Suppl 1:S27-35. PMID: 26304969. doi: https://dx.doi.org/10.1370/afm.1819.

638. Tang PC, Overhage JM, Chan AS, Brown NL, Aghighi B, Entwistle MP, et al. Online disease management of diabetes: engaging and motivating patients online with enhanced resources-diabetes (EMPOWER-D), a randomized controlled trial. J Am Med Inform Assoc. 2013 May 01;20(3):526-34. PMID: 23171659. doi: https://dx.doi.org/10.1136/amiajnl-2012-001263.

639. Tanner JL, Craig CB, Bartolucci AA, Allon M, Fox LM, Geiger BF, et al. The effect of a self-monitoring tool on self-efficacy, health beliefs, and adherence in patients receiving hemodialysis. J Ren Nutr. 1998 Oct;8(4):203-11. PMID: 9776797.

640. Tate DF, Jackvony EH, Wing RR. Effects of Internet behavioral counseling on weight loss in adults at risk for type 2 diabetes: a randomized trial. Jama. 2003 Apr 09;289(14):1833-6. PMID: 12684363.

641. Taylor PJ, Thompson CH, Luscombe-Marsh ND, Wycherley TP, Wittert G, Brinkworth GD. Efficacy of Real-Time Continuous Glucose Monitoring to Improve Effects of a Prescriptive Lifestyle Intervention in Type 2 Diabetes: A Pilot Study. Diabetes Ther. 2019 Apr;10(2):509-22. PMID: 30706365. doi: https://dx.doi.org/10.1007/s13300-019-0572-z.

642. Taylor CB, Agras WS, Losch M, Plante TG, Burnett K. Improving the effectiveness of computer-assisted weight loss. Behavior Therapy. 1991 Spr 1991;22(2):229-36. PMID: 1991-28298-001. doi: 10.1016/S0005-7894(05)80178-9.

643. Terazawa T, Mamiya T, Masui S, Nakamura M. [The effect of smoking cessation counseling at health checkup]. Sangyo Eiseigaku Zasshi. 2001 Nov;43(6):207-13. PMID: 11802453.

644. Thabit H, Prabhu JN, Mubita W, Fullwood C, Azmi S, Urwin A, et al. Use of factory-calibrated real-time continuous glucose monitoring improves time in target and HbA1c in a multiethnic cohort of adolescents and young adults with type 1 diabetes: The MILLENNIALS study. Diabetes Care. 2020;43(10):2537-43. PMID: 32723843. doi: 10.2337/dc20-0736.

645. Thielen V, Scheen A, Bringer J, Renard E. Attempt to improve glucose control in type 2 diabetic patients by education about real-time glucose monitoring. Diabetes Metab. 2010 Jun;36(3):240-3. PMID: 20452799. doi: https://dx.doi.org/10.1016/j.diabet.2010.03.002.

646. Thomas JG, Bond DS, Raynor HA, Papandonatos GD, Wing RR. Comparison of Smartphone-Based Behavioral Obesity Treatment With Gold Standard Group Treatment and Control: A Randomized Trial. Obesity. 2019 04;27(4):572-80. PMID: 30779333. doi: https://dx.doi.org/10.1002/oby.22410.

647. Thomas JG, Raynor HA, Bond DS, Luke AK, Cardoso CC, Wojtanowski AC, et al. Weight loss and frequency of body-weight self-monitoring in an online commercial weight management program with and without a cellular-connected 'smart' scale: a randomized pilot study. Obes. 2017 12;3(4):365-72. PMID: 29259794. doi: https://dx.doi.org/10.1002/osp4.132.

648. Thomas JG, Leahey TM, Wing RR. An automated internet behavioral weight-loss program by physician referral: a randomized controlled trial. Diabetes Care. 2015 Jan;38(1):9-15. PMID: 25404659. doi: https://dx.doi.org/10.2337/dc14-1474.

649. Thomas D, Vydelingum V, Lawrence J. E-mail contact as an effective strategy in the maintenance of weight loss in adults. J Hum Nutr Diet. 2011 Feb;24(1):32-8. PMID: 21114552. doi: https://dx.doi.org/10.1111/j.1365-277X.2010.01123.x.

650. Thomsen JL, Parner ET, Karlsmose B, Thulstrup AM, Lauritzen T, Engberg M. Effect of preventive health screening on long-term primary health care utilization. A randomized controlled trial. Fam Pract. 2005 Jun;22(3):242-8. PMID: 15814585.

651. Thorndike AN, Sonnenberg L, Healey E, Myint UK, Kvedar JC, Regan S. Prevention of weight gain following a worksite nutrition and exercise program: a randomized controlled trial. American Journal of Preventive Medicine. 2012 Jul;43(1):27-33. PMID: 22704742. doi: https://dx.doi.org/10.1016/j.amepre.2012.02.029.

652. Tiessen AH, Smit AJ, Broer J, Groenier KH, van der Meer K. Randomized controlled trial on cardiovascular risk management by practice nurses supported by self-monitoring in primary care. BMC Fam Pract. 2012 Sep 04;13:90. PMID: 22947269. doi: https://dx.doi.org/10.1186/1471-2296-13-90.

653. Tildesley HD, Wright AM, Chan JHM, Mazanderani AB, Ross SA, Tildesley HG, et al. A comparison of internet monitoring with continuous glucose monitoring in insulin-requiring type 2 diabetes mellitus. Canadian Journal of Diabetes. 2013;37(5):305-8. PMID: 24500556. doi: 10.1016/j.jcjd.2013.05.006.

654. Tomita MR, Tsai BM, Fisher NM, Kumar NA, Wilding G, Stanton K, et al. Effects of multidisciplinary Internet-based program on management of heart failure. J Multidiscip Healthc. 2008 Dec 01;2009(2):13-21. PMID: 20505786.

655. Towfighi A, Cheng EM, Ayala-Rivera M, Barry F, McCreath H, Ganz DA, et al. Effect of a Coordinated Community and Chronic Care Model Team Intervention vs Usual Care on Systolic Blood Pressure in Patients With Stroke or Transient Ischemic Attack: The SUCCEED Randomized Clinical Trial. JAMA netw. 2021 02 01;4(2):e2036227. PMID: 33587132. doi: https://dx.doi.org/10.1001/jamanetworkopen.2020.36227.

656. Tremblay A, Taghizadeh N, Huang J, Kasowski D, MacEachern P, Burrowes P, et al. A Randomized Controlled Study of Integrated Smoking Cessation in a Lung Cancer Screening Program. J Thorac Oncol. 2019 09;14(9):1528-37. PMID: 31077790. doi: https://dx.doi.org/10.1016/j.jtho.2019.04.024.

657. Treskes RW, van Winden LAM, van Keulen N, van der Velde ET, Beeres S, Atsma DE, et al. Effect of Smartphone-Enabled Health Monitoring Devices vs Regular Follow-up on Blood Pressure Control Among Patients After Myocardial Infarction: A Randomized Clinical Trial. JAMA netw. 2020 04 01;3(4):e202165. PMID: 32297946. doi: https://dx.doi.org/10.1001/jamanetworkopen.2020.2165.

658. True MW, Strickland LE, Lewi JE, Sterling LM, Dai H, Haas RW, et al. Impact of a Diabetes Risk Score on Lifestyle Education and Patient Adherence (IDEA) in Prediabetes: A Multisite Randomized Controlled Trial. Mil Med. 2015 Oct;180(10):1091-7. PMID: 26444473. doi: https://dx.doi.org/10.7205/MILMED-D-14-00656.

659. Tsang MW, Mok M, Kam G, Jung M, Tang A, Chan U, et al. Improvement in diabetes control with a monitoring system based on a hand-held, touch-screen electronic diary. J Telemed Telecare. 2001;7(1):47-50. PMID: 11265938.

660. Tumminia A, Milluzzo A, Festa C, Fresa R, Pintaudi B, Scavini M, et al. Efficacy of flash glucose monitoring in pregnant women with poorly controlled pregestational diabetes (FlashMom): A randomized pilot study. Nutr Metab Cardiovasc Dis. 2021 Mar 24;24:24. PMID: 33975741. doi: https://dx.doi.org/10.1016/j.numecd.2021.03.013.

661. Turner BJ, Hollenbeak CS, Liang Y, Pandit K, Joseph S, Weiner MG. A randomized trial of peer coach and office staff support to reduce coronary heart disease risk in African-Americans with uncontrolled hypertension. Journal of General Internal Medicine. 2012 Oct;27(10):1258-64. PMID: 22570108.

662. Tzourio C, Hanon O, Godin O, Soumare A, Dufouil C. Impact of home blood pressure monitoring on blood pressure control in older individuals: a French randomized study. J Hypertens. 2017 03;35(3):612-20. PMID: 27984412. doi: https://dx.doi.org/10.1097/HJH.0000000000001191.

663. Ulm K, Huntgeburth U, Gnahn H, Briesenick C, Purner K, Middeke M. Effect of an intensive nurse-managed medical care programme on ambulatory blood pressure in hypertensive patients. Arch Cardiovasc Dis. 2010 Mar;103(3):142-9. PMID: 20417445. doi: https://dx.doi.org/10.1016/j.acvd.2010.01.006.

664. Unick JL, O'Leary KC, Bond DS, Wing RR. Physical activity enhancement to a behavioral weight loss program for severely obese individuals: A preliminary investigation. ISRN Obes. 2012 Sep 05;05:05. PMID: 24379985. doi: https://dx.doi.org/10.5402/2012/465158.

665. Valle CG, Deal AM, Tate DF. Preventing weight gain in African American breast cancer survivors using smart scales and activity trackers: a randomized controlled pilot study. J. 2017 02;11(1):133-48. PMID: 27631874. doi: https://dx.doi.org/10.1007/s11764-016-0571-2.

666. van Limpt PM, Harting J, van Assema P, Ruland E, Kester A, Gorgels T, et al. Effects of a brief cardiovascular prevention program by a health advisor in primary care; the 'Hartslag Limburg' project, a cluster randomized trial. Prev Med. 2011 Dec;53(6):395-401. PMID: 21925203. doi: https://dx.doi.org/10.1016/j.ypmed.2011.08.031.

667. VanWormer JJ, Martinez AM, Benson GA, Crain AL, Martinson BC, Cosentino DL, et al. Telephone counseling and home telemonitoring: the Weigh by Day Trial. Am J Health Behav. 2009 Jul-Aug;33(4):445-54. PMID: 19182989.

668. Vassy JL, Christensen KD, Schonman EF, Blout CL, Robinson JO, Krier JB, et al. The Impact of Whole-Genome Sequencing on the Primary Care and Outcomes of Healthy Adult Patients: A Pilot Randomized Trial. Annals of Internal Medicine. 2017 08 01;167(3):159-69. PMID: 28654958. doi: https://dx.doi.org/10.7326/M17-0188.

669. Vaz CL, Carnes N, Pousti B, Zhao H, Williams KJ. A randomized controlled trial of an innovative, user-friendly, interactive smartphone app-based lifestyle intervention for weight loss. Obesity Science and Practice. 2021. doi: 10.1002/osp4.503.

670. Vernooij JW, Kaasjager HA, van der Graaf Y, Wierdsma J, Grandjean HM, Hovens MM, et al. Internet based vascular risk factor management for patients with clinically manifest vascular disease: randomised controlled trial. Bmj. 2012 Jun 12;344:e3750. PMID: 22692651. doi: https://dx.doi.org/10.1136/bmj.e3750.

671. Vetter W, Hess L, Brignoli R. Influence of self-measurement of blood pressure on the responder rate in hypertensive patients treated with losartan: results of the SVATCH Study. Standard vs Automatic Treatment Control of COSAAR in Hypertension. J Hum Hypertens. 2000 Apr;14(4):235-41. PMID: 10805048. doi: 10.1038/sj.jhh.1000977 10.1038/sj.jhh.1000977.

672. Victor RG, Ravenell JE, Freeman A, Leonard D, Bhat DG, Shafiq M, et al. Effectiveness of a barber-based intervention for improving hypertension control in black men: the BARBER-1 study: a cluster randomized trial. Arch Intern Med. 2011 Feb 28;171(4):342-50. PMID: 20975012. doi: https://dx.doi.org/10.1001/archinternmed.2010.390.

673. Voils CI, Coffman CJ, Grubber JM, Edelman D, Sadeghpour A, Maciejewski ML, et al. Does Type 2 Diabetes Genetic Testing and Counseling Reduce Modifiable Risk Factors? A Randomized Controlled Trial of Veterans. Journal of General Internal Medicine. 2015 Nov;30(11):1591-8. PMID: 25876740. doi: https://dx.doi.org/10.1007/s11606-015-3315-5.

674. Von Korff M, Katon WJ, Lin EH, Ciechanowski P, Peterson D, Ludman EJ, et al. Functional outcomes of multi-condition collaborative care and successful ageing: results of randomised trial. Bmj. 2011 Nov 10;343:d6612. PMID: 22074851. doi: https://dx.doi.org/10.1136/bmj.d6612.

675. von Storch K, Graaf E, Wunderlich M, Rietz C, Polidori MC, Woopen C. Telemedicine-Assisted Self-Management Program for Type 2 Diabetes Patients. Diabetes Technol Ther. 2019 09;21(9):514-21. PMID: 31287736. doi: https://dx.doi.org/10.1089/dia.2019.0056.

676. Voormolen DN, DeVries JH, Sanson RME, Heringa MP, e Valk HW, Kok M, et al. Continuous glucose monitoring during diabetic pregnancy (GlucoMOMS): A multicentre randomized controlled trial. Diabetes Obes Metab. 2018 08;20(8):1894-902. PMID: 29603547. doi: https://dx.doi.org/10.1111/dom.13310.

677. Vuong K, Armstrong BK, McGeechan K, Cust AE. Personalized melanoma risk assessments and tailored prevention advice: a pragmatic randomized controlled trial in Australian general practice. Fam Pract. 2019 03 20;36(2):237-46. PMID: 29800131. doi: https://dx.doi.org/10.1093/fampra/cmy040.

678. Wade AN. The effect of blood glucose self-monitoring in patients with non-insulin treated type 2 diabetes [D.Phil.]. Ann Arbor: University of Oxford (United Kingdom); 2005.

679. Wagener TL. Genuine vs DrAFT-CS: A randomized clinical trial comparing computer and live personalized feedback interventions for high-risk drinking among college students: ProQuest Information & Learning; 2013.

680. Wagner PJ, Dias J, Howard S, Kintziger KW, Hudson MF, Seol YH, et al. Personal health records and hypertension control: a randomized trial. Journal of the American Medical Informatics Association : JAMIA. 2012;19(4):626‐34. PMID: CN-00854369. doi: 10.1136/amiajnl-2011-000349.

681. Wakefield BJ, Holman JE, Ray A, Scherubel M, Adams MR, Hillis SL, et al. Effectiveness of home telehealth in comorbid diabetes and hypertension: a randomized, controlled trial. Telemed J E Health. 2011 May;17(4):254-61. PMID: 21476945. doi: https://dx.doi.org/10.1089/tmj.2010.0176.

682. Waki K, Fujita H, Uchimura Y, Omae K, Aramaki E, Kato S, et al. DialBetics: A Novel Smartphone-based Self-management Support System for Type 2 Diabetes Patients. J Diabetes Sci Technol. 2014 Mar;8(2):209-15. PMID: 24876569.

683. Walker TC, Yucha CB. Continuous glucose monitors: use of waveform versus glycemic values in the improvements of glucose control, quality of life, and fear of hypoglycemia. J Diabetes Sci Technol. 2014 May;8(3):488-93. PMID: 24876611. doi: https://dx.doi.org/10.1177/1932296814528434.

684. Walker WB, Franzini LR. Low-risk aversive group treatments, physiological feedback, and booster sessions for smoking cessation. Behavior Therapy. 1985;16(3):263-74.

685. Wang J, Cai C, Padhye N, Orlander P, Zare M. A Behavioral Lifestyle Intervention Enhanced With Multiple-Behavior Self-Monitoring Using Mobile and Connected Tools for Underserved Individuals With Type 2 Diabetes and Comorbid Overweight or Obesity: Pilot Comparative Effectiveness Trial. JMIR Mhealth Uhealth. 2018 Apr 10;6(4):e92. PMID: 29636320. doi: https://dx.doi.org/10.2196/mhealth.4478.

686. Wang G, Zhang Z, Feng Y, Sun L, Xiao X, Wang G, et al. Telemedicine in the Management of Type 2 Diabetes Mellitus. Am J Med Sci. 2017 Jan;353(1):1-5. PMID: 28104096. doi: https://dx.doi.org/10.1016/j.amjms.2016.10.008.

687. Wang C, Gordon ES, Norkunas T, Wawak L, Liu CT, Winter M, et al. A randomized trial Examining The Impact Of Communicating Genetic And Lifestyle Risks For Obesity. Obesity. 2016 Dec;24(12):2481-90. PMID: 27891830. doi: https://dx.doi.org/10.1002/oby.21661.

688. Wangberg SC. An Internet-based diabetes self-care intervention tailored to self-efficacy. Health Education Research. 2008 Feb;23(1):170-9. PMID: 17412717.

689. Wanyenze RK, Kamya MR, Fatch R, Mayanja-Kizza H, Baveewo S, Szekeres G, et al. Abbreviated HIV counselling and testing and enhanced referral to care in Uganda: a factorial randomised controlled trial. Lancet Glob Health. 2013 Sep;1(3):e137-45. PMID: 25104262. doi: https://dx.doi.org/10.1016/S2214-109X(13)70067-6.

690. Watanabe M, Okayama A, Shimamoto K, Ueshima H. Short-term effectiveness of an individual counseling program for impaired fasting glucose and mild type 2 diabetes in Japan: a multi-center randomized control trial. Asia Pac J Clin Nutr. 2007;16(3):489-97. PMID: 17704031.

691. Watson AJ, Singh K, Myint UK, Grant RW, Jethwani K, Murachver E, et al. Evaluating a web-based self-management program for employees with hypertension and prehypertension: a randomized clinical trial. Am Heart J. 2012 Oct;164(4):625-31. PMID: 23067923. doi: https://dx.doi.org/10.1016/j.ahj.2012.06.013.

692. Watson M, Duvivier V, Wade Walsh M, Ashley S, Davidson J, Papaikonomou M, et al. Family history of breast cancer: what do women understand and recall about their genetic risk? J Med Genet. 1998 Sep;35(9):731-8. PMID: 9733031.

693. Wei KS, Ibrahim NE, Kumar AA, Jena S, Chew V, Depa M, et al. Habits Heart App for Patient Engagement in Heart Failure Management: Pilot Feasibility Randomized Trial. JMIR Mhealth Uhealth. 2021 01 20;9(1):e19465. PMID: 33470941. doi: https://dx.doi.org/10.2196/19465.

694. Weinberg DS, Myers RE, Keenan E, Ruth K, Sifri R, Ziring B, et al. Genetic and environmental risk assessment and colorectal cancer screening in an average-risk population: a randomized trial. Annals of Internal Medicine. 2014 Oct 21;161(8):537-45. PMID: 25329201. doi: https://dx.doi.org/10.7326/M14-0765.

695. Weinberger M, Murray MD, Marrero DG, Brewer N, Lykens M, Harris LE, et al. Effectiveness of pharmacist care for patients with reactive airways disease: a randomized controlled trial. Jama. 2002 Oct 02;288(13):1594-602. PMID: 12350190.

696. Welschen LMC, Bot SDM, Kostense PJ, Dekker JM, Timmermans DRM, Van Der Weijden T, et al. Effects of cardiovascular disease risk communication for patients with type 2 diabetes on risk perception in a randomized controlled trial: The @RISK study. Diabetes Care. 2012;35(12):2485-92. PMID: 22923669. doi: 10.2337/dc11-2130.

697. Wenger NS, Linn LS, Epstein M, Shapiro MF. Reduction of high-risk sexual behavior among heterosexuals undergoing HIV antibody testing: A randomized clinical trial. American Journal of Public Health. 1991;81(12):1580-5. PMID: 1993-14908-001. doi: 10.2105/AJPH.81.12.1580.

698. Wengreen HJ, Nix E, Madden GJ. The effect of social norms messaging regarding skin carotenoid concentrations among college students. Appetite. 2017 09 01;116:39-44. PMID: 28455259. doi: https://dx.doi.org/10.1016/j.appet.2017.04.027.

699. Wessel SE, van der Hoeven NV, Cammenga M, van Montfrans GA, van den Born BJ. 'Diagnostic mode' improves adherence to the home blood pressure measurement schedule. Blood Press Monit. 2012 Oct;17(5):214-9. PMID: 22850440. doi: https://dx.doi.org/10.1097/MBP.0b013e328357352a.

700. West DS, Krukowski RA, Finkelstein EA, Stansbury ML, Ogden DE, Monroe CM, et al. Adding Financial Incentives to Online Group-Based Behavioral Weight Control: An RCT. American Journal of Preventive Medicine. 2020 08;59(2):237-46. PMID: 32446752. doi: https://dx.doi.org/10.1016/j.amepre.2020.03.015.

701. West DS, Stansbury M, Krukowski RA, Harvey J. Enhancing group-based internet obesity treatment: A pilot RCT comparing video and text-based chat. Obes. 2019 Dec;5(6):513-20. PMID: 31890241. doi: https://dx.doi.org/10.1002/osp4.371.

702. Wevers MR, Aaronson NK, Verhoef S, Bleiker EM, Hahn DE, Kuenen MA, et al. Impact of rapid genetic counselling and testing on the decision to undergo immediate or delayed prophylactic mastectomy in newly diagnosed breast cancer patients: findings from a randomised controlled trial. Br J Cancer. 2014 Feb 18;110(4):1081-7. PMID: 24423928. doi: https://dx.doi.org/10.1038/bjc.2013.805.

703. Weymiller AJ, Montori VM, Jones LA, Gafni A, Guyatt GH, Bryant SC, et al. Helping patients with type 2 diabetes mellitus make treatment decisions: statin choice randomized trial. Arch Intern Med. 2007 May 28;167(10):1076-82. PMID: 17533211.

704. White N, Carnahan J, Nugent CA, Iwaoka T, Dodson MA. Management of obese patients with diabetes mellitus: comparison of advice education with group management. Diabetes Care. 1986 Sep-Oct;9(5):490-6. PMID: 3769719.

705. Whittemore R, Vilar-Compte M, De La Cerda S, Delvy R, Jeon S, Burrola-Mendez S, et al. Si, Yo Puedo Vivir Sano con Diabetes! A Self-Management Randomized Controlled Pilot Trial for Low-Income Adults with Type 2 Diabetes in Mexico City. Curr. 2020 May;4(5):nzaa074. PMID: 32368713. doi: https://dx.doi.org/10.1093/cdn/nzaa074.

706. Whittle J, Schapira MM, Fletcher KE, Hayes A, Morzinski J, Laud P, et al. A randomized trial of peer-delivered self-management support for hypertension. Am J Hypertens. 2014 Nov;27(11):1416-23. PMID: 24755206. doi: https://dx.doi.org/10.1093/ajh/hpu058.

707. Wickramasinghe N, John B, George J, Vogel D. Achieving Value-Based Care in Chronic Disease Management: Intervention Study. JMIR Diabetes. 2019 May 03;4(2):e10368. PMID: 31066699. doi: https://dx.doi.org/10.2196/10368.

708. Wild SH, Hanley J, Lewis SC, McKnight JA, McCloughan LB, Padfield PL, et al. Supported Telemonitoring and Glycemic Control in People with Type 2 Diabetes: The Telescot Diabetes Pragmatic Multicenter Randomized Controlled Trial. PLoS Med. 2016 Jul;13(7):e1002098. PMID: 27458809. doi: https://dx.doi.org/10.1371/journal.pmed.1002098.

709. Wilkins T, Gillies RA, Panchal P, Patel M, Warren P, Schade RR. Colorectal cancer risk information presented by a nonphysician assistant does not increase screening rates. Can Fam Physician. 2014 Aug;60(8):731-8. PMID: 25122819.

710. Williams DM, Dunsiger S, Miranda R, Jr G, C. J E, J. A M, et al. Recommending self-paced exercise among overweight and obese adults: a randomized pilot study. Ann Behav Med. 2015 Apr;49(2):280-5. PMID: 25223963. doi: https://dx.doi.org/10.1007/s12160-014-9642-7.

711. Williams A, Manias E, Walker R, Gorelik A. A multifactorial intervention to improve blood pressure control in co-existing diabetes and kidney disease: a feasibility randomized controlled trial. J Adv Nurs. 2012 Nov;68(11):2515-25. PMID: 22335395. doi: https://dx.doi.org/10.1111/j.1365-2648.2012.05950.x.

712. Williams DR, Lewis NM. Effectiveness of nutrition counseling in young adult males. Nutrition Research. 2002;22(8):911-7. doi: 10.1016/S0271-5317(02)00399-8.

713. Wills AM, Garry J, Hubbard J, Mezoian T, Breen CT, Ortiz-Miller C, et al. Nutritional counseling with or without mobile health technology: a randomized open-label standard-of-care-controlled trial in ALS. BMC Neurol. 2019 May 29;19(1):104. PMID: 31142272. doi: https://dx.doi.org/10.1186/s12883-019-1330-6.

714. Winett RA, Anderson ES, Wojcik JR, Winett SG, Moore S, Blake C. Guide to Health: A Randomized Controlled Trial of the Effects of a Completely WEB-Based Intervention on Physical Activity, Fruit and Vegetable Consumption, and Body Weight. Transl Behav Med. 2011 Mar;1(1):165-74. PMID: 23503089.

715. Wing RR, Tate DF, Espeland MA, Lewis CE, LaRose JG, Gorin AA, et al. Innovative Self-Regulation Strategies to Reduce Weight Gain in Young Adults: The Study of Novel Approaches to Weight Gain Prevention (SNAP) Randomized Clinical Trial. JAMA Internal Medicine. 2016 06 01;176(6):755-62. PMID: 27136493. doi: https://dx.doi.org/10.1001/jamainternmed.2016.1236.

716. Wing RR, Crane MM, Thomas JG, Kumar R, Weinberg B. Improving weight loss outcomes of community interventions by incorporating behavioral strategies. American Journal of Public Health. 2010 Dec;100(12):2513-9. PMID: 20966375. doi: https://dx.doi.org/10.2105/AJPH.2009.183616.

717. Wing RR, Tate DF, Gorin AA, Raynor HA, Fava JL. A self-regulation program for maintenance of weight loss. N Engl J Med. 2006 Oct 12;355(15):1563-71. PMID: 17035649.

718. Wing RR, Jeffery RW. Prescribed "breaks" as a means to disrupt weight control efforts. Obes Res. 2003 Feb;11(2):287-91. PMID: 12582226.

719. Wing RR, Jeffery RW, Hellerstedt WL, Burton LR. Effect of frequent phone contacts and Optional Food Provision on maintenance of weight loss. Ann Behav Med. 1996 Sep;18(3):172-6. PMID: 24203769. doi: https://dx.doi.org/10.1007/BF02883394.

720. Wing RR, Epstein LH, Nowalk MP, Scott N. Self-regulation in the treatment of Type II diabetes. Behavior Therapy. 1988;19(1):11-23.

721. Wing RR, Epstein LH, Nowalk MP, Scott N, Koeske R, Hagg S. Does self-monitoring of blood glucose levels improve dietary compliance for obese patients with type II diabetes? Am J Med. 1986 Nov;81(5):830-6. PMID: 3535493.

722. Wing RR, Epstein LH, Shapira B, Koeske R. Contingent therapist contact in a behavioral weight control program. J Consult Clin Psychol. 1984 Aug;52(4):710-1. PMID: 6470299.

723. Winzenberg T, Oldenburg B, Frendin S, De Wit L, Riley M, Jones G. The effect on behavior and bone mineral density of individualized bone mineral density feedback and educational interventions in premenopausal women: a randomized controlled trial [NCT00273260]. BMC Public Health. 2006 Jan 23;6:12. PMID: 16430773.

724. Wister A, Loewen N, Kennedy-Symonds H, McGowan B, McCoy B, Singer J. One-year follow-up of a therapeutic lifestyle intervention targeting cardiovascular disease risk. Cmaj. 2007 Oct 09;177(8):859-65. PMID: 17923653.

725. Witteman HO, Fuhrel-Forbis A, Wijeysundera HC, Exe N, Dickson M, Holtzman L, et al. Animated randomness, avatars, movement, and personalization in risk graphics. Journal of Medical Internet Research. 2014 Mar 18;16(3):e80. PMID: 24642037. doi: https://dx.doi.org/10.2196/jmir.2895.

726. Woe Sook K, Sakano Y. Cognitive-behavioral intervention to chronic disease patients. Japanese Journal of Psychosomatic Medicine. 1996;36(1):27-32.

727. Wolinsky FD, Lou Y, Edmonds SW, Hall SF, Jones MP, Wright NC, et al. Activating Patients With a Tailored Bone Density Test Results Letter and Educational Brochure: the PAADRN Randomized Controlled Trial. J Clin Densitom. 2017 Oct - Dec;20(4):464-71. PMID: 27647261. doi: https://dx.doi.org/10.1016/j.jocd.2016.08.012.

728. Wong EM, Leung DYP, Chair SY, Sit JWH. Effects of a Web-Based Educational Support Intervention on Total Exercise and Cardiovascular Risk Markers in Adults With Coronary Heart Disease. Worldviews Evid Based Nurs. 2020 Aug;17(4):283-92. PMID: 32772509. doi: https://dx.doi.org/10.1111/wvn.12456.

729. Wong SS, Ng CJ, Liew SM, Hussein N. Effectiveness of a colour coded HbA1c graphical record in improving diabetes control in people with type 2 diabetes: a randomized control trial. Diabetes Res Clin Pract. 2012 Feb;95(2):e41-4. PMID: 22119614. doi: https://dx.doi.org/10.1016/j.diabres.2011.11.001.

730. Worth R, Home PD, Johnston DG, Anderson J, Ashworth L, Burrin JM, et al. Intensive attention improves glycaemic control in insulin-dependent diabetes without further advantage from home blood glucose monitoring: results of a controlled trial. Br Med J (Clin Res Ed). 1982 Oct 30;285(6350):1233-40. PMID: 6812820.

731. Wyke S, Hunt K, Gray CM, Fenwick E, Bunn C, Donnan PT, et al. Football Fans in Training (FFIT): a randomised controlled trial of a gender-sensitised weight loss and healthy living programme for men. NIHR Journals Library. 2015 01;01:01. PMID: 25654156. doi: https://dx.doi.org/10.3310/phr03020.

732. Xu R, Xing M, Javaherian K, Peters R, Ross W, Bernal-Mizrachi C. Improving HbA<sub>1c</sub> with Glucose Self-Monitoring in Diabetic Patients with EpxDiabetes, a Phone Call and Text Message-Based Telemedicine Platform: A Randomized Controlled Trial. Telemed J E Health. 2020 06;26(6):784-93. PMID: 31621523. doi: https://dx.doi.org/10.1089/tmj.2019.0035.

733. Xue F, Yao W, Lewin RJ. A randomised trial of a 5 week, manual based, self-management programme for hypertension delivered in a cardiac patient club in Shanghai. BMC Cardiovasc Disord. 2008 May 06;8:10. PMID: 18460201. doi: https://dx.doi.org/10.1186/1471-2261-8-10.

734. Yancy WS, Jr S, P. A R, C H, V Y, J Z, et al. Effect of Escalating Financial Incentive Rewards on Maintenance of Weight Loss: A Randomized Clinical Trial. JAMA netw. 2019 11 01;2(11):e1914393. PMID: 31675083. doi: https://dx.doi.org/10.1001/jamanetworkopen.2019.14393.

735. Yancy WS, Jr S, P. A W, L H, V Y, L Z, et al. Financial incentive strategies for maintenance of weight loss: results from an internet-based randomized controlled trial. Nutr Diabetes. 2018 05 25;8(1):33. PMID: 29795365. doi: https://dx.doi.org/10.1038/s41387-018-0036-y.

736. Yang Y, Lee EY, Kim HS, Lee SH, Yoon KH, Cho JH. Effect of a Mobile Phone-Based Glucose-Monitoring and Feedback System for Type 2 Diabetes Management in Multiple Primary Care Clinic Settings: Cluster Randomized Controlled Trial. JMIR Mhealth Uhealth. 2020 02 26;8(2):e16266. PMID: 32130172. doi: https://dx.doi.org/10.2196/16266.

737. Yang Y, Tian CH, Cao J, Huang XJ. Research on the application of health management model based on the perspective of mobile health. Medicine (Baltimore). 2019 Aug;98(33):e16847. PMID: 31415411. doi: https://dx.doi.org/10.1097/MD.0000000000016847.

738. Yang X, Tian H, Zhang F, Zhang C, Li Y, Leng J, et al. A randomised translational trial of lifestyle intervention using a 3-tier shared care approach on pregnancy outcomes in Chinese women with gestational diabetes mellitus but without diabetes. J. 2014 Oct 28;12:290. PMID: 25349017. doi: https://dx.doi.org/10.1186/s12967-014-0290-2.

739. Yarkova NA, Borovkov NN. [Adherence to the Treatment in Patients with Type 2 Diabetes Mellitus and Ways to Its Optimization]. Klin Med (Mosk). 2016;94(9):688-92. PMID: 30296367.

740. Yaron M, Roitman E, Aharon-Hananel G, Landau Z, Ganz T, Yanuv I, et al. Effect of Flash Glucose Monitoring Technology on Glycemic Control and Treatment Satisfaction in Patients With Type 2 Diabetes. Diabetes Care. 2019 07;42(7):1178-84. PMID: 31036546. doi: https://dx.doi.org/10.2337/dc18-0166.

741. Yasmin M-T, C. G B, M. J H. The effect of dietary intervention on changes in total cholesterol, blood pressure and weight in a Cambridge study. Int J Clin Pract. 1998 Jun;52(4):241-5. PMID: 9744148.

742. Yasutake K, Umeki Y, Horita N, Morita R, Murata Y, Ohe K, et al. A self-monitoring urinary salt excretion level measurement device for educating young women about salt reduction: A parallel randomized trial involving two groups. J Clin Hypertens (Greenwich). 2019 06;21(6):730-8. PMID: 31058457. doi: https://dx.doi.org/10.1111/jch.13545.

743. Yasutake K, Miyoshi E, Misumi Y, Kajiyama T, Fukuda T, Ishii T, et al. Self-monitoring of urinary salt excretion as a method of salt-reduction education: a parallel, randomized trial involving two groups. Public Health Nutr. 2018 08;21(12):2164-73. PMID: 29458447. doi: https://dx.doi.org/10.1017/S1368980018000095.

744. Yen T, Qin F, Sundaram V, Asiimwe E, Storage T, Ladabaum U. Randomized Controlled Trial of Personalized Colorectal Cancer Risk Assessment vs Education to Promote Screening Uptake. Am J Gastroenterol. 2021 02 01;116(2):391-400. PMID: 33009045. doi: https://dx.doi.org/10.14309/ajg.0000000000000963.

745. Yen LL, Patrick WK, Chie WC. Comparison of relaxation techniques, routine blood pressure measurements, and self-learning packages in hypertension control. Prev Med. 1996 May-Jun;25(3):339-45. PMID: 8781012.

746. Yeoh E, Lim BK, Fun S, Tong J, Yeoh LY, Sum CF, et al. Efficacy of self-monitoring of blood glucose versus retrospective continuous glucose monitoring in improving glycaemic control in diabetic kidney disease patients. Nephrology. 2018 Mar;23(3):264-8. PMID: 27933715. doi: https://dx.doi.org/10.1111/nep.12978.

747. Yew TW, Chi C, Chan SY, van Dam RM, Whitton C, Lim CS, et al. A randomized controlled trial to evaluate the effects of a smartphone application–based lifestyle coaching program on gestational weight gain, glycemic control, and maternal and neonatal outcomes in women with gestational diabetes mellitus: The smart-gdm study. Diabetes Care. 2021;44(2):456-63. PMID: 33184151. doi: 10.2337/dc20-1216.

748. Yi SS, Tabaei BP, Angell SY, Rapin A, Buck MD, Pagano WG, et al. Self-blood pressure monitoring in an urban, ethnically diverse population: a randomized clinical trial utilizing the electronic health record. Circ Cardiovasc Qual Outcomes. 2015 Mar;8(2):138-45. PMID: 25737487. doi: https://dx.doi.org/10.1161/CIRCOUTCOMES.114.000950.

749. Yoo HJ, An HG, Park SY, Ryu OH, Kim HY, Seo JA, et al. Use of a real time continuous glucose monitoring system as a motivational device for poorly controlled type 2 diabetes. Diabetes Res Clin Pract. 2008 Oct;82(1):73-9. PMID: 18701183. doi: https://dx.doi.org/10.1016/j.diabres.2008.06.015.

750. Yoo JS, Kim EJ, Lee SJ. [The effects of a comprehensive life style modification program on glycemic control and stress response in type 2 diabetes]. Taehan Kanho Hakhoe Chi. 2006 Aug;36(5):751-60. PMID: 16953132.

751. Yoon KH, Kim HS. A short message service by cellular phone in type 2 diabetic patients for 12 months. Diabetes Res Clin Pract. 2008 Feb;79(2):256-61. PMID: 17988756.

752. Young LA, Buse JB, Weaver MA, Vu MB, Mitchell CM, Blakeney T, et al. Glucose self-monitoring in non-insulin-treated patients with type 2 diabetes in primary care settings: A randomized trial. JAMA Internal Medicine. 2017;177(7):920-9. PMID: 28600913. doi: 10.1001/jamainternmed.2017.1233.

753. Young L, Hertzog M, Barnason S. Effects of a home-based activation intervention on self-management adherence and readmission in rural heart failure patients: the PATCH randomized controlled trial. BMC Cardiovasc Disord. 2016 09 08;16(1):176. PMID: 27608624. doi: https://dx.doi.org/10.1186/s12872-016-0339-7.

754. Yu Y, Yan Q, Li H, Li H, Wang L, Wang H, et al. Effects of mobile phone application combined with or without self-monitoring of blood glucose on glycemic control in patients with diabetes: A randomized controlled trial. J. 2019 Sep;10(5):1365-71. PMID: 30815973. doi: https://dx.doi.org/10.1111/jdi.13031.

755. Yun YH, Kang E, Cho YM, Park SM, Kim YJ, Lee HY, et al. Efficacy of an Electronic Health Management Program for Patients With Cardiovascular Risk: Randomized Controlled Trial. Journal of Medical Internet Research. 2020 01 22;22(1):e15057. PMID: 32012053. doi: https://dx.doi.org/10.2196/15057.

756. Zairina E, Abramson MJ, McDonald CF, Li J, Dharmasiri T, Stewart K, et al. Telehealth to improve asthma control in pregnancy: A randomized controlled trial. Respirology. 2016 07;21(5):867-74. PMID: 27037722. doi: https://dx.doi.org/10.1111/resp.12773.

757. Zaleski AL, Taylor BA, Park CL, Santos LP, Panza G, Kramarz M, et al. Using the immediate blood pressure benefits of exercise to improve exercise adherence among adults with hypertension: a randomized clinical trial. J Hypertens. 2019 09;37(9):1877-88. PMID: 31058797. doi: https://dx.doi.org/10.1097/HJH.0000000000002115.

758. Zha P, Qureshi R, Porter S, Chao YY, Pacquiao D, Chase S, et al. Utilizing a Mobile Health Intervention to Manage Hypertension in an Underserved Community. West J Nurs Res. 2020 03;42(3):201-9. PMID: 31057081. doi: https://dx.doi.org/10.1177/0193945919847937.

759. Zhai Y, Yu W. A Mobile App for Diabetes Management: Impact on Self-Efficacy Among Patients with Type 2 Diabetes at a Community Hospital. Med Sci Monit. 2020 Nov 16;26:e926719. PMID: 33196634. doi: https://dx.doi.org/10.12659/MSM.926719.

760. Zhang X, Jiang D, Wang X. The effects of the instantaneous scanning glucose monitoring system on hypoglycemia, weight gain, and health behaviors in patients with gestational diabetes: a randomised trial. Ann. 2021 05 07;05:07. PMID: 33977739. doi: https://dx.doi.org/10.21037/apm-21-439.

761. Zhang D, Huang QF, Li Y, Wang JG. A randomized controlled trial on home blood pressure monitoring and quality of care in stage 2 and 3 hypertension. Hypertens Res. 2021 May;44(5):533-40. PMID: 33442031. doi: https://dx.doi.org/10.1038/s41440-020-00602-0.

762. Zhang L, He X, Shen Y, Yu H, Pan J, Zhu W, et al. Effectiveness of Smartphone App-Based Interactive Management on Glycemic Control in Chinese Patients With Poorly Controlled Diabetes: Randomized Controlled Trial. Journal of Medical Internet Research. 2019 12 09;21(12):e15401. PMID: 31815677. doi: https://dx.doi.org/10.2196/15401.

763. Zhang Y, Chu L. Effectiveness of Systematic Health Education Model for Type 2 Diabetes Patients. Int. 2018;2018:6530607. PMID: 30147724. doi: https://dx.doi.org/10.1155/2018/6530607.

764. Zhang DA, Katznelson L, Li M. Postprandial glucose monitoring further improved glycemia, lipids, and weight in persons with type 2 diabetes mellitus who had already reached hemoglobin A1c goal. J Diabetes Sci Technol. 2012 Mar 01;6(2):289-93. PMID: 22538137.

765. Zullig LL, Sanders LL, Shaw RJ, McCant F, Danus S, Bosworth HB. A randomised controlled trial of providing personalised cardiovascular risk information to modify health behaviour. J Telemed Telecare. 2014 Apr;20(3):147-52. PMID: 24647384. doi: https://dx.doi.org/10.1177/1357633X14528446.

766. Zutz A, Ignaszewski A, Bates J, Lear SA. Utilization of the internet to deliver cardiac rehabilitation at a distance: a pilot study. Telemed J E Health. 2007 Jun;13(3):323-30. PMID: 17603835.

767. Zylinska E, Kosior DA. Education, cardiovascular risk factors and blood pressure control in hypertensive outpatients. Kardiol Pol. 2018;76(11):1551-61. PMID: 30091135. doi: https://dx.doi.org/10.5603/KP.a2018.0157.
