# Supplementary material for: Use of Biological Feedback as a Health Behavior Change Technique in Adults: Scoping Review
Source: J Med Internet Res. 2023 Sep 25;25:e44359. doi: 10.2196/44359 (PMC10562972; doi:10.2196/44359)
Supplement: Multimedia Appendix 5 [file jmir_v25i1e44359_app5.docx]

**Multimedia Appendix 5: Domains of research that have used biological feedback to promote health behavior change in adults (N=767)**

| **Domains** | **Frequency, n (%)^a^** | **Year of First Publication** |
| --- | --- | --- |
| Diabetes | 233 (30.4%) | 1982 |
| CVD | 175 (22.8%) | 1975 |
| Overweight/Obesity | 115 (15.0%) | 1972 |
| Substance Use | 66 (8.6%) | 1977 |
| Health Promotion | 63 (8.2%) | 1984 |
| Cancer | 34 (4.4%) | 1998 |
| Pregnancy/Postpartum | 31 (4.0%) | 1982 |
| Osteoporosis | 19 (2.5%) | 1997 |
| Infectious Disease | 10 (1.3%) | 1991 |
| Mental Health | 9 (1.2%) | 1990 |
| Kidney Disease | 8 (1.0%) | 1998 |
| Metabolic Syndrome | 7 (0.9%) | 2010 |
| Respiratory Disease | 7 (0.9%) | 2002 |
| Neurodegenerative disease | 3 (0.4%) | 2008 |
| Oral health | 2 (0.3%) | 2019 |
| Arthritis | 1 (0.1%) | 2018 |
| Urinary Incontinence | 1 (0.1%) | 2012 |

^a^The percentage of total studies adds up to be more than 100% because some studies fell under multiple domains.
