# Supplementary material for: Use of Biological Feedback as a Health Behavior Change Technique in Adults: Scoping Review
Source: J Med Internet Res. 2023 Sep 25;25:e44359. doi: 10.2196/44359 (PMC10562972; doi:10.2196/44359)
Supplement: Multimedia Appendix 6 [file jmir_v25i1e44359_app6.docx]

**Multimedia Appendix 6:** **Outcomes targeted by biological feedback interventions in adults (N=767).**

| **Outcomes** | **Frequency, n (%)^a^** |
| --- | --- |
| Glycemic control | 194 (25.3%) |
| CVD management | 162 (21.1%) |
| Weight management | 161 (21.0%) |
| Behavior change | 102 (13.3%) |
| Substance use reduction | 77 (10.0%) |
| Bone health | 17 (2.2%) |
| Health risk improvement | 10 (1.3%) |
| Infectious disease management | 10 (1.3%) |
| UV exposure | 10 (1.3%) |
| Cancer risk | 9 (1.2%) |
| Physical ability | 9 (1.2%) |
| Pregnancy/infant health | 7 (0.9%) |
| Respiratory disease management | 7 (0.9%) |
| Mental health improvement | 3 (0.4%) |
| Oral health | 1 (0.1%) |

^a^The percentage of total studies adds up to be more than 100% because some studies had multiple outcomes.
