# Supplementary material for: Use of Biological Feedback as a Health Behavior Change Technique in Adults: Scoping Review
Source: J Med Internet Res. 2023 Sep 25;25:e44359. doi: 10.2196/44359 (PMC10562972; doi:10.2196/44359)
Supplement: Multimedia Appendix 7 [file jmir_v25i1e44359_app7.docx]

**Multimedia Appendix 7**: **Biomarkers on which biological feedback has been provided (N=767).**

| **Biomarker** | **Frequency, n (%)^a^** |
| --- | --- |
| Weight/BMI | 282 (36.8%) |
| Blood pressure | 238 (31.0%) |
| Glucose | 227 (29.6%) |
| Lipids | 100 (13.0%) |
| HbA1c | 51 (6.6%) |
| Genetics | 50 (6.5%) |
| Heart rate | 40 (5.2%) |
| Carbon monoxide | 35 (4.6%) |
| Pulmonary function | 32 (4.2%) |
| Bone mass/density | 18 (2.3%) |
| Body composition | 15 (2.0%) |
| Nutrient biomarkers | 14 (1.8%) |
| Alcohol intake | 13 (1.7%) |
| Infectious disease result | 12 (1.6%) |
| ECG | 10 (1.3%) |
| Kidney function | 9 (1.2%) |
| Skin damage | 9 (1.2%) |
| Cotinine/Nicotine | 8 (1.0%) |
| Artery plaque | 7 (0.9%) |
| Liver function | 5 (0.7%) |
| Cancer screening biomarkers | 4 (0.5%) |
| Medication concentration | 4 (0.5%) |
| Vision | 4 (0.5% |
| Insulin function | 3 (0.4%) |
| Dental health | 2 (0.3%) |
| Drug intake | 2 (0.3%) |
| Fetal ultrasound | 2 (0.3%) |
| Hormone level | 2 (0.3%) |
| Immune function | 2 (0.3%) |
| Haemoglobin | 1 (0.1%) |
| Blood clotting | 1 (0.1%) |
| EEG | 1 (0.1%) |
| Electromyography (EMG) | 1 (0.1%) |
| Environmental exposure | 1 (0.1%) |
| Hearing | 1 (0.1%) |
| Inflammatory marker | 1 (0.1%) |

^a^The percentage of total studies adds up to be more than 100% because some studies provided feedback on multiple biomarkers.
