# Supplementary material for: Use of Biological Feedback as a Health Behavior Change Technique in Adults: Scoping Review
Source: J Med Internet Res. 2023 Sep 25;25:e44359. doi: 10.2196/44359 (PMC10562972; doi:10.2196/44359)
Supplement: Multimedia Appendix 8 [file jmir_v25i1e44359_app8.docx]

**Multimedia Appendix 8**: **Behaviors targeted by biological feedback interventions (N=767).**

| **Behavior** | **Frequency, n (%)**^a^ |
| --- | --- |
| Diet | 472 (61.5%) |
| Physical activity | 417 (54.4%) |
| Smoking reduction | 154 (20.1%) |
| Treatment adherence | 143 (18.6%) |
| None | 90 (11.7%) |
| Alcohol reduction | 75 (9.8%) |
| Stress management | 55 (7.2%) |
| Medical consult | 44 (5.7%) |
| Sleep | 14 (1.8%) |
| Sun protection | 11 (1.4%) |
| Drug reduction | 7 (0.9%) |
| Safe sex | 7 (0.9%) |
| Safety behaviors | 5 (0.7%) |
| Skin self-exam | 5 (0.7%) |
| Dental care | 3 (0.4%) |

^a^The percentage of total studies adds up to be more than 100% because some studies targeted multiple behaviors.
